# Population genomics reveal distinct and diverging populations of *An. minimus* in Cambodia

**DOI:** 10.1038/s42003-022-04259-y

**Published:** 2022-11-28

**Authors:** Brandyce St. Laurent, Nick Harding, Nick Deason, Kolthida Oy, Chea Sok Loeun, Men Sary, Rous Sunly, Sen Nhep, Eleanor Drury, Kirk Rockett, Siv Sovannaroth, Sonia Goncalves, Dominic Kwiatkowski, Alistair Miles

**Affiliations:** 1 Wellcome Sanger Institute, Parasites and Microbes, Natural Genetic Variation, Cambridge, UK; 2 previously NIH NIAID Laboratory of Malaria and Vector Research, Bethesda, MD USA; 3 National Center for Parasitology, Entomology and Malaria Control (CNM), Phnom Penh, Cambodia

**Keywords:** Genetic variation, Malaria, Biodiversity

## Abstract

*Anopheles minimus* is an important malaria vector throughout its wide geographic range across Southeast Asia. Genome sequencing could provide important insights into the unique malaria transmission dynamics in this region, where many vector species feed and rest outdoors. We describe results from a study using Illumina deep whole-genome sequencing of 302 wild-caught *An. minimus* collected from three Cambodian provinces over several years (2010, 2014, 2016) and seasons to examine the level of population structure and genetic diversity within this species. These specimens cluster into four distinct populations of *An. minimus s.s*., with two populations overlapping geographically. We describe the underlying genetic diversity and divergence of these populations and investigated the genetic variation in genes known to be involved in insecticide resistance. We found strong signals of selection within these *An. minimus* populations, most of which were present in the two Northeastern Cambodian populations and differ from those previously described in African malaria vectors. Cambodia is the focus of the emergence and spread of drug-resistant malaria parasites, so understanding the underlying genetic diversity and resilience of the vectors of these parasites is key to implementing effective malaria control and elimination strategies. These data are publicly available as part of the MalariaGEN Vector Observatory, an open access resource of genome sequence data.

## Abstract (web summary)

Using whole genome sequencing and broad sampling across the nation, the genetic diversity of the malaria mosquito *An. minimus* across Cambodia is described.

## Introduction

*An. minimus* is a small mosquito that plays a major role in sustaining malaria transmission across the Greater Mekong Subregion (GMS). This species is commonly associated with human-altered habitats, able to breed in rice paddies and forest-fringe habitats. The GMS is becoming increasingly fragmented as deforestation and agriculture create patchwork landscapes^1–3^. Historically, malaria transmission in this region has been dominated by the major vector species *An. dirus*, a forest-dwelling, highly competent vector, and human-attracted mosquito. More recently, land use change has resulted in the decline of *An. dirus* populations and has shed light on the role of other vectors in malaria transmission, species like *An. minimus*, that are able to breed in human-associated habitats and quickly adapt to changing environments^4–6^. Vectors that occupy different seasonal and ecological niches can also sustain malaria transmission and change the dynamics of seasonal transmission patterns. While *An. dirus* is considered the major vector in the GMS, there are many other species that have been shown to be susceptible to malaria parasites that play a role in local transmission^2,7–9^.

To further complicate malaria transmission in this region, successful lineages of multi-drug resistant malaria parasites have continued to emerge from the area along the Thai-Cambodian border^10–13^ and spread through the GMS. Lab infection experiments have shown that *Anopheles minimus* is able to be infected by and transmit the parasites in Cambodia that have been shown to have slow clearance in response to the Artemisinin combined therapy drug treatment^14^, and these parasites are co-circulating in areas where *An. minimus* is active and abundant. All five human malarias are transmitted in Cambodia^15^, with *Plasmodium vivax* infections about as prevalent as those from *Plasmodium falciparum*^16^. *An. minimus* is known to transmit *P. falciparum* and *P. vivax* throughout its wide geographic range, which spans from China down into the Malay archipelago, and from western India to Vietnam^17^.

Many *Anopheles* species in Southeast Asia are members of cryptic species complexes, groups of isomorphic species that are molecularly and often behaviorally distinct. In other *Anopheles* cryptic species groups like the Gambiae complex, some closely related cryptic species have been shown to exchange genetic material from almost every region of the genome, described as having “porous species boundaries”^18^. The level of gene flow between what is typically thought of as isolated species has important implications for the spread of genes relevant to malaria transmission, such as insecticide resistance alleles, or genes that confer susceptibility or resistance to malaria parasites. Population genomic studies of *An. gambiae s.l*. across the African continent have shown that geographically distinct populations have similar signatures of selection in response to decades of insecticide-based vector control^19^, sharing common haplotypes that confer insecticide resistance over thousands of kilometers. *Anopheles minimus* is a member of such a species complex with at least three recognized cryptic species: *An. minimus* s.s. (*An. minimus* A), *An. harrisoni* (*An. minimus C*), and *An. yaeyamaensis* (*An. minimus E*)^20,21^ and is also in the Funestus group, somewhat closely related to *An. funestus*, an important African malaria vector. Only *An. minimus s.s*. has been reported from collections in Cambodia^5^, and is considered to have greater indoor and human biting preferences than other members of this species complex. We currently know very little about the population structure of *An. minimus* and other important vectors occurring across Southeast Asia.

Cambodia is transitioning from focusing on malaria control to malaria pre-elimination, with a goal of eradicating malaria from the country within the next five years. Drug-resistant parasites, prevalent *P. vivax* infections, and outdoor malaria transmission are all factors that complicate the goal of malaria elimination in this country. Despite outdoor biting behaviors of many local vector species, insecticide-treated nets are still the primary vector control method implemented in the GMS and do have some protective efficacy against *P. falciparum* infection^22^. Investigations into baseline rates of insecticide resistance have shown that *An. minimus* from different regions has varying levels of susceptibility to DDT and pyrethroid insecticides, with some evidence of resistance in populations in Cambodia, Thailand, Laos, India, and Vietnam^23–25^. Additionally, there is very limited regulation of the use of insecticides for public health and agriculture in Cambodia^26^.

In the context of the spread of parasites that threaten our best and last-line defense against malaria, we need to know what the population history of the vectors of these parasites looks like across this same space. In this study, we seek to describe the population structure, diversity, and divergence of populations of *An. minimus* that were active during the rise and spread of drug-resistant parasites in Cambodia. We hope to contribute to a better understanding of malaria transmission in Southeast Asia.

## Results

### Population sampling and sequencing

We generated whole genome sequence data from 302 wild-caught individual *An. minimus* female mosquitoes collected from five different field sites in Cambodia using the Illumina HiSeq 2000 platform with 150 bp paired-end reads with a target coverage of 30X for each. Mosquito collections in Thmar Da, in Eastern Cambodia, were done in 2010. Longitudinal monthly collections were performed from February 2014 to January 2015 in two sites in each of the Preah Vihear, and Ratanakiri provinces. Quarterly collections were also done in 2016 in one site in Preah Vihear province, Cambodia.

### Variant discovery

The methods for sequencing and variant calling closely follow those of the Anopheles gambiae 1000 Genomes project phase 2 (Ag1000G)^27^. Sequence reads were aligned to the *An. minimus* reference genome AminM1^28^. We restricted our analysis to the largest 40 contigs, which cover 96.6% of the AminM1 reference genome, as many smaller-sized contigs can confound diversity and divergence calculations. We found that 138,161,075 (75.4%) of sites within these 40 largest contigs pass our site filters and thus were accessible to SNP calling. Of these, we discovered 38,000,285 segregating single nucleotide polymorphisms (SNPs) that passed all of our quality control filters of 55,307,039 total segregating SNPs. 13.4% of these SNPs were multiallelic, with 32,906,471 biallelic SNPs. There were 4,807,355 triallelic and 286,459 quadriallelic SNPs. A total of 100,160,790 sites were invariant. The median genome-wide coverage was 35X.

### Population structure

**Fig. 1:**
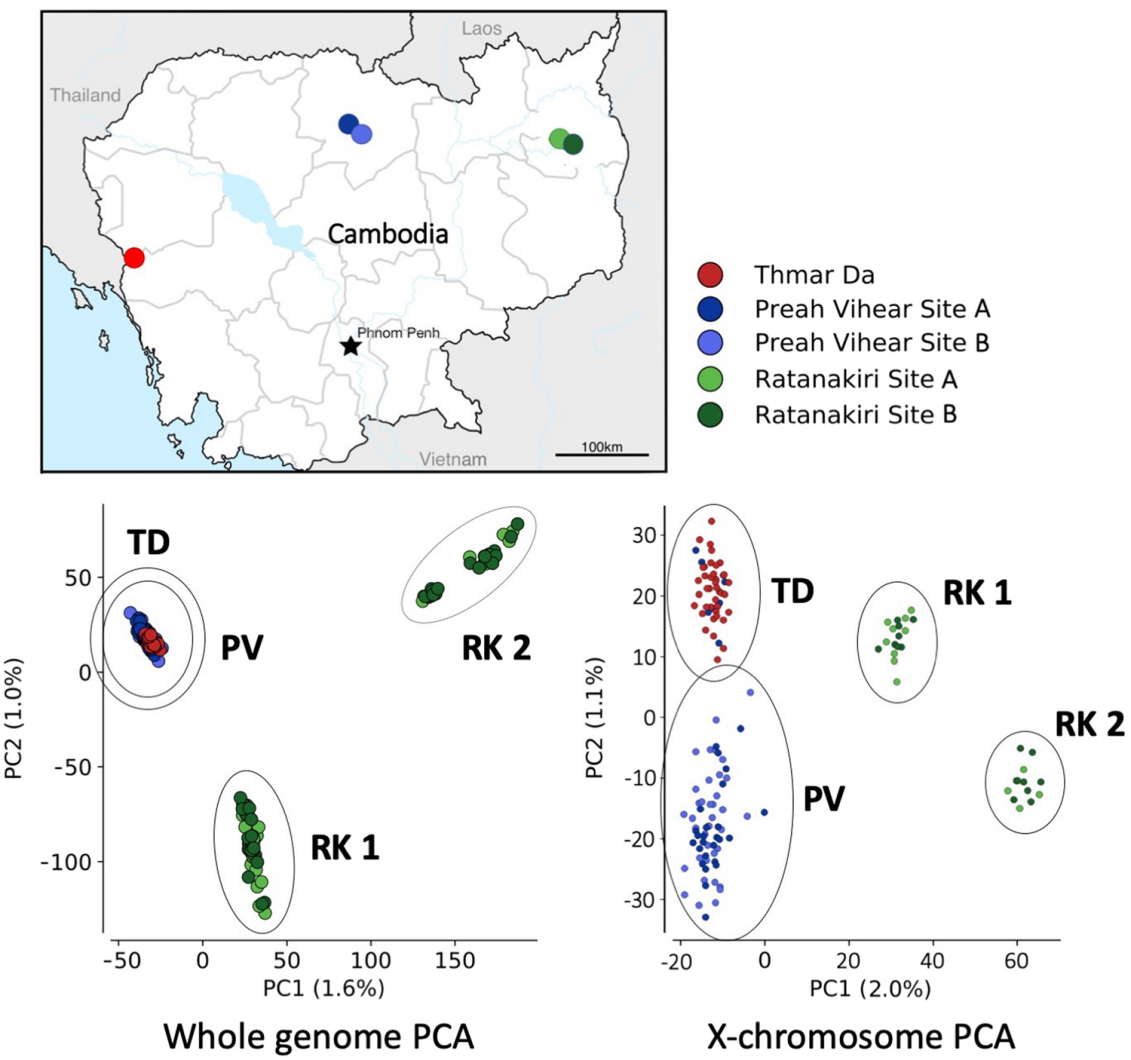
Population structure of An. minimus in Cambodia. The map indicates the five Cambodian collection sites. Principal component analysis (PCA) of whole genome sequences of 283 individual *An. minimus s.s*. collected in five villages in Cambodia shows that there is a distinct population structure and three populations. When performing the same PCA on a large X-chromosomal contig (KB664054), these individuals break into four populations: TD from the West, PV from the northern province in Preah Vihear, and RK1 and RK2, both collected in two sites in Ratanakiri province in the Northeast.

A principal component analysis (PCA) over biallelic SNPs distributed over the genome of 302 individual field-collected mosquitoes showed that there is clear population structure of *An. minimus* in Cambodia. Samples collected from five sites in three provinces split into three distinct clusters; here, we report on 283 individuals that could be clearly assigned to these clusters (Fig. 1), excluding 9 anomalous and 10 outlying individuals. One cluster includes all samples from the western collection site Thmar Da and the northern collection sites in Preah Vihear province, with two further clusters with samples from Ratanakiri province in the northeast. These clusters split primarily along the first and second principal components. This was a surprising finding because this population structure did not correlate to the geographic sampling of these mosquitoes. Individuals collected from the western and northern sites cluster tightly together despite being hundreds of kilometers apart.

To further explore this population structure, we performed the same PCA over individual contigs from different regions of the genome. Performing PCA over the largest X-chromosomal contig KB664054 resulted in a splitting of the western and northern samples, indicating four distinct populations of *An. minimus* in Cambodia (Fig. 1). PCA from this contig on a quickly evolving sex chromosome revealed more population structure compared to autosomal contigs. The populations defined by these PCA clusters are designated in this study as TD from Thmar Da, in Western Cambodia (*n* = 41), on the Thai-Cambodian border, PV from the Northern province Preah Vihear (*n* = 156), and the two distinct populations collected in Ratanakiri province in the Northeast, each including individuals collected at both collection sites, these are designated as populations RK1 (*n* = 58) and RK2 (*n* = 28).

To confirm our results from PCA, we also performed an admixture analysis. We ran admixture on each of the largest 10 contigs for values of K between 2 and 6 (Supplemental Fig. 1). At *K* = 2, the samples from Northeastern Cambodia split from Northern and Western Cambodia samples. At *K* = 3, the two different groups in Ratanakiri were separated, consistent with the PCA results. At *K* = 4, there was some evidence for geographical population structure between the Western TD and Northern PV populations, but the admixture results did not perfectly correspond with geographic sampling, with some evidence of mixed ancestry in the PV samples. Again, this is consistent with the PCA groupings, with the generally weaker evidence of geographic population structure between TD and PV. A cross-validation analysis showed the lowest cross-validation error for *K* = 2 and *K* = 3, consistent with the strongest evidence for population structure between the two RK groups and other populations. Cross-validation error was higher at *K* = 4, consistent with the weaker differentiation between TD and PV. At no point was their an indication of admixture between RK1 and RK2.

**Fig. 2:**
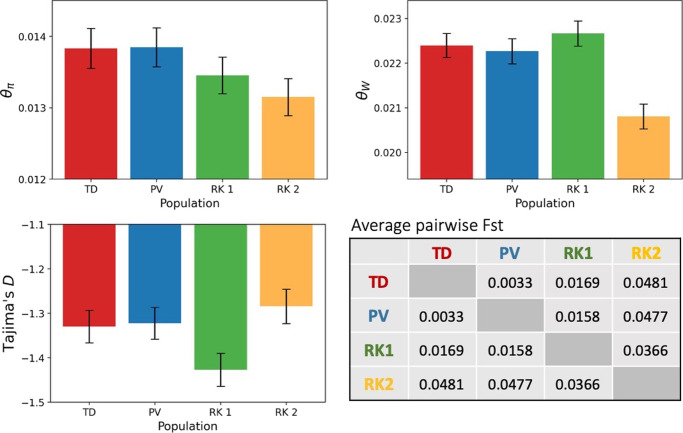
Population diversity and divergence. Nucleotide diversity (*π*), Watterson’s Theta (*θ*_W_), and Tajima’s D statistics were calculated over fourfold degenerate sites on autosomal contigs. The error bars indicate 95% confidence intervals calculated over 100 bootstrap replicates over samples. An average pairwise Fst in the table here was calculated in 20 kb windows over the largest contig KB663610.

To examine population differentiation, we computed differences in allele frequencies between each population using Pairwise Fst. Pairwise Fst between all 4 populations over the largest contig, KB663610, representing 16% of the *An. minimus* genome, (Fig. 2) shows that differentiation was relatively low between populations of TD and PV with an average pairwise Fst of 0.003, while the difference between RK2 and the other three populations is tenfold higher, around 0.03. Pairwise Fst estimates comparing these populations over other large *An. minimus* contigs indicate a similar level of differentiation, with average pairwise Fst values over 0.03 (Supplementary Data 3). The two sympatric populations from the Ratanakiri collection sites are as differentiated from each other as they are from the northern and western clusters.

This level of differentiation of RK2, even from the RK1 population, might indicate an emerging cryptic species within *An. minimus* A or a newly diverging clade. RK1 and RK2 are sympatric populations, both being collected in the same two sites in Northeastern Cambodia. The differences seen here between RK1 and RK2 populations are consistent with cryptic taxa in other anopheline groups. For example, in the *An. gambiae* complex, the level of differentiation between recently diverged sibling species *An. coluzzii* and *An. gambiae* in West Africa is approximately 0.03^19^.

### Population diversity and variation

To characterize population diversity among these populations, nucleotide diversity (*π*), Watterson’s Theta (θ_W_), and Tajima’s D statistics were calculated over 4-fold degenerate sites on autosomal contigs larger than 2 megabases with 100 bootstrap replicates over samples. These 17 contigs represent 80% of the *Anopheles minimus* genome (Fig. 2). The populations were downsampled for these calculations to have sizes equal to that of the smallest population RK2 (*n* = 28).

There are small but significant differences in the magnitude of the genetic diversity summary statistics between these four different populations. In particular, there were notable differences between the putatively cryptic taxa RK1 and RK2, two populations that were collected in the same sites in Northeastern Cambodia. RK1 had higher levels of nucleotide diversity and lower levels of Tajima’s D than RK2. These differences are consistent with different population size histories between these sympatric groups. Lower values of Tajima’s D suggest stronger population growth in RK1. Comparing all four populations, higher levels of genetic diversity indicate larger effective population sizes of TD and PV compared to RK1 and RK2.

RK2 has a significantly reduced nucleotide diversity and Watterson’s Theta compared to the other three populations. This may indicate a smaller population size and a recent bottleneck of the RK2 population in Cambodia. All four *An. minimus* populations have a negative Tajima’s D, indicating an excess of rare variants, particularly in RK1. This suggests recent population expansions in all populations.

### Signals of evolutionary selection

We used Fst to scan across the *Anopheles minimus* genome to look for regions of the genome with increased differentiation. When we scanned the genome using pairwise Fst, there were no apparent long signals of differentiation that might indicate a large inversion or other structural variants, known to be major drivers of adaptive evolution in other *Anopheles* groups. To investigate increased differentiation across large regions of the genome, we performed scans of nucleotide diversity (π), Watterson’s Theta (θ_W_), and Tajima’s D over the largest 14 contigs (representing 80% of the *An. minimus* genome). As with the Fst scans, there were no large regions of higher differentiation in any of the populations that might indicate major structural variants or inversions (Supplementary Figs. 2–4).

Whole-genome sequencing allowed us to identify pointed signals occurring across the entire genome using scans of average pairwise Fst. Isolated points of high differentiation were compared over single contigs with average pairwise Fst calculated over windows of 1000 SNPs each and plotted over whole contigs. The strongest signals, indicated by the highest Fst value at the peak of a strong signal of differentiation, were ranked and compared. The five top signals in each of the six comparisons between the four populations are listed in Table 1. These isolated points of high differentiation are one indication of a signal of evolutionary selection. The most differentiated regions by Fst occurred when comparing the RK2 population to the other three populations, with the highest selection peaks with pairwise Fst over 0.4. RK2 also had more distinct signals of selection when compared to the other populations than RK1. Since these signals of differentiation were highly localized, we could look to known gene annotations and gene predictions across the AminM1 reference genome to see which genes were within 100 kbp of the peaks of these signals. We have noted candidate genes of interest that were near the strongest Fst signal peaks and also had known or predicted gene functions (Table 1, Supplementary Fig. 6, Supplementary Fig. 8).

**Table 1:** The top five Fst signals of high differentiation within each of six population comparisons are reported here.

Population comparison	Contig	Signal Position (Mbp)	Fst Value	Candidate genes in region	Gene(s)	Gene functions
RK1 v RK2	KB663622	1.3	0.54	UDP-glucuronosyltransferase	AMIN004611	Pyrethroid resistance, detoxification
RK1 v RK2	KB663622	2.3	0.52	Metalloendopeptidase, aminopeptidase N	AMIN004703, AMIN004700, AMIN004701	Pyrethroid resistance, decreased susceptibility to BT toxin
RK1 v RK2	KB664266	6	0.49	Ecdysteroid UDP-glucosyltransferase	AMIN009683	Pyrethroid resistance
RK1 v RK2	KB663832	5.7	0.49	histone acetyltransferase, α-tubulin, CHK domain-containing protein	AMIN016061, AMIN000390, AMIN000391	Dieldrin resistance, organophosphate interactions, mitochondrial function
RK1 v RK2	KB663610	2	0.48	Peptidase S1 domain-containing protein	AMIN002286	Immune response to parasites, detoxification
PV v RK2	KB663622	1.25	0.54	UDP-glucuronosyltransferase	AMIN004611	Pyrethroid resistance, detoxification
PV v RK2	KB664277	0.4	0.53	COP9 signalosome complex subunit	AMIN001615	Hormone and neural signaling
PV v RK2	KB663610	0.1	0.53	cyclic AMP-responsive element-binding protein, JNK1/MAPK8-associated membrane protein	AMIN002152, AMIN002155	G protein-coupled receptor (GPCR) signal activated transcription factor, stress response
PV v RK2	KB664266	6	0.51	Ecdysteroid UDP-glucosyltransferase	AMIN009683	Pyrethroid resistance
PV v RK2	KB663622	2.2	0.5	Methionine aminopeptidase	AMIN004701	Pyrethroid resistance, decreased susceptibility to BT toxin
PV v RK1	KB663832	1.4	0.39	Elongation of very long chain fatty acids protein	AMIN000203	Lipid concentration, metabolic detoxification
PV v RK1	KB663832	3.7	0.38			Mixed, unspecified products
PV v RK1	KB664054	2.6	0.34	NADPH–cytochrome P450 reductase, 39S ribosomal protein L22	AMIN005245, AMIN005257	Insecticide resistance, oxidative stress
PV v RK1	KB663622	1.8	0.33	csk homologous kinase domain-containing protein, translation initiation factor 1A	AMIN004659, AMIN004663	JH signaling, hemocyte activation
PV v RK1	KB663655	1	0.33			Mixed, unspecified products
TD v RK2	KB663610	0.1	0.55	Cyclic AMP-responsive element-binding protein, JNK1/MAPK8-associated membrane protein	AMIN002152, AMIN002155	G protein-coupled receptor (GPCR) signal activated transcription factor, stress response
TD v RK2	KB663832	1.3	0.55	Sodium-independent sulfate anion transporter, Protein arginine methyltransferase RmtB, Phosphate carrier- mitochondrial	AMIN000191, AMIN015783, AMIN015787	Signal transduction, phosphate carrier activated in mosquitoes by malaria infection
TD v RK2	KB663622	1.25	0.52	UDP-glucuronosyltransferase	AMIN004611	Pyrethroid resistance, detoxification
TD v RK2	KB664266	6	0.5	Ecdysteroid UDP-glucosyltransferase	AMIN009683	Pyrethroid resistance
TD v RK2	KB664277	0.4	0.49	COP9 signalosome complex subunit	AMIN001615	Hormone and neural signaling
TD v RK1	KB663832	1.25	0.5	UDP-glucuronosyltransferase	AMIN004611	Pyrethroid resistance, detoxification
TD v RK1	KB664054	2.6	0.49	NADPH–cytochrome P450 reductase, 39S ribosomal protein L22	AMIN005245, AMIN005257	Insecticide resistance, oxidative stress
TD v RK1	KB663832	3.75	0.4			Mixed, unspecified products
TD v RK1	KB663622	1.8	0.34	CHK domain-containing protein, translation initiation factor 1A	AMIN004659, AMIN004663	Mitochondrial function
TD v RK1	KB663622	3.1	0.32	Carbohydrate sulfotransferase	AMIN004763	Detoxification
TD v PV	KB663622	3.1	0.125	Carbohydrate sulfotransferase	AMIN004763	Detoxification
TD v PV	KB663832	0.6	0.049	Carboxylesterase	AMIN000169	Pyrethroid resistance and detoxification
TD v PV	KB664054	2.7	0.042	NADPH–cytochrome P450 reductase, 39S ribosomal protein L22	AMIN005245, AMIN005257	Insecticide resistance, oxidative stress
TD v PV	KB663666	0.5	0.032	Solute carrier family 6 neurotransmitter transporter	AMIN009210	Neural signaling
TD v PV	KB663832	1.35	0.027	Sodium-independent sulfate anion transporter, Protein arginine methyltransferase RmtB, Phosphate carrier- mitochondrial	AMIN000191, AMIN015783, AMIN015787	Signal transduction, a phosphate carrier activated in mosquitoes by malaria infection

Signals were identified using Fst scans in 1000 SNP windows over AminM1 contigs that were larger than 2 Mb. Genes and gene predictions near the signal peaks were identified using the VePathDB genome browser.

**Fig. 3:**
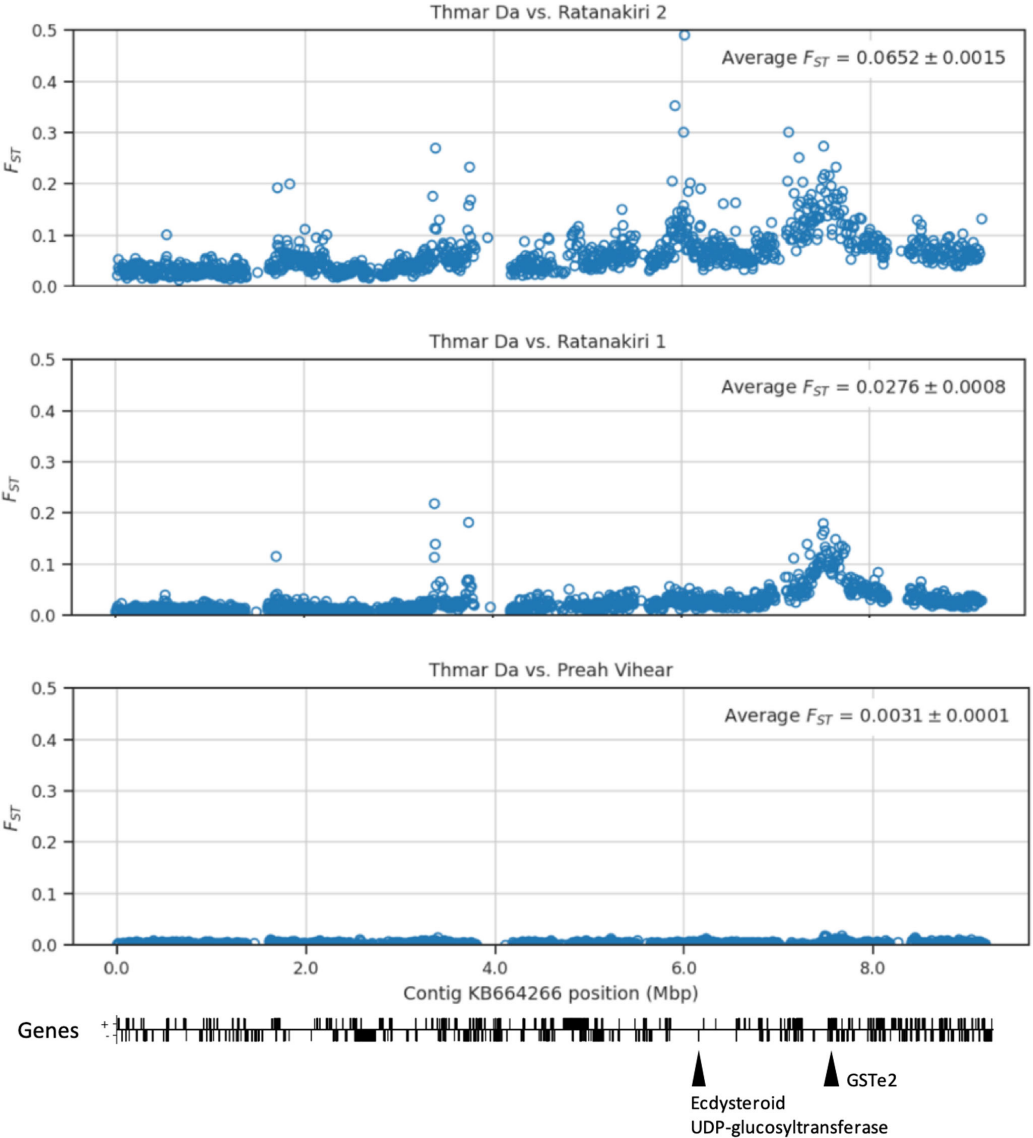
Signals of selection over a single autosomal contig. Pairwise Fst was calculated in 1000 SNP windows over autosomal contig KB664266, comparing the Thmar Da population to the three other populations, Ratanakiri 2, Ratanakiri 1, and Preah Vihear. There is almost no indication of selection when comparing Thmar Da with Preah Vihear. There is a strongly supported signal of differentiation in both Ratanakiri 1 and Ratanakiri 2 populations at 7.5 Mbp, which is in the same location as a cluster of GSTe genes, including *GSTe2*, which are known to be involved in metabolic resistance to DDT and pyrethroids. The signal with the highest Fst peak here in RK2, at 6 Mbp is close to an Ecdysteroid UDP-glucosyltransferase gene, shown to confer pyrethroid insecticide resistance in other anophelines. These are a few of many selection signals identified in this study that may be associated with insecticide pressure on these *An. minimus* populations.

There is almost no indication of selection when comparing the Thmar Da population with Preah Vihear, with all but one signal with Fst values below 0.05. The one strong signal between TD and PV (Fst = 0.125) is near a Carbohydrate sulfotransferase, which is involved in detoxification processes. Comparing TD to RK1 and RK2 reveals multiple strong signals of selection, some which are present in both Northeastern populations, as well as many unique RK2-specific signals (Fig. 3, Supplementary Fig. 5).

Many of the strongest signals identified in this study may be associated with insecticide pressure on these *An. minimus* populations. The strongest selection signals in every population comparison were close to genes that are involved in detoxification, signal transduction, and adaptations to oxidative stress, or have been functionally validated to have mutations that confer resistance to insecticides (Table 1). Some signals of interest include a strongly supported signal of selection in both RK1 and RK2 populations at 7.5 Mbp on the contig KB664266, which is in the same location as a cluster of glutathione-S-transferases, including *GSTe2*, which has been shown to be involved in the metabolism of DDT and pyrethroids, mutations in which mediate metabolic insecticide resistance^29^. The signal with the highest pairwise Fst peak on the same contig KB664266, at 6 Mbp is an RK2-specific signal and close to an Ecdysteroid UDP-glucosyltransferase gene, which has been shown to confer pyrethroid insecticide resistance in *An. stephensi*^30^.

Another notable signal is between the RK1 and RK2 populations on the contig KB663610, a Peptidase S1 domain-containing protein AMIN002286, which has been shown to be involved in response to parasite pathogens in insects^31^. The signals of selection observed in this study are mostly distinct from the main selection signals seen in *An. gambiae* complex mosquitoes^19^, the primary vectors of *Plasmodium falciparum* in Africa.

### Insecticide resistance

We report here variants at known insecticide resistance-associated alleles for each of the four *An. minimus* populations. Variants occurring at a frequency of more than 2% in at least one of the four populations are reported in the known insecticide-resistance-associated genes *Ace1*, *Rdl*, *KDR*, and *GSTe2* (Supplementary Data 2). *GSTe2* mutants are present in multiple populations, at a low rate, and there are a few individuals in Thmar Da and Preah Vihear with the *Rdl* resistance mutation, which is known to confer resistance to cyclodiene insecticides, despite evidence from other studies that species in this region lack this resistance mutation^32^. We did not investigate copy number variation, which is one mechanism by which *GSTe2* confers insecticide resistance. These SNP variants indicate variation throughout these insecticide-resistance-associated genes, and though most of these populations do not currently have high rates of validated insecticide resistance-associated mutations, this underlying variation provides the potential for structural and transcriptional events resulting in greater levels of insecticide resistance in *An. minimus* populations.

## Discussion

This is the first study investigating the population genomics of *An. minimus*. We examined the whole genomes of 283 wild-collected female mosquitoes from five different sites in Cambodia over several years. This important vector species appears to be highly structured within Cambodia, with a division of West, North, and North-eastern populations. This structure cannot be fully accounted for by geography. The RK1 and RK2 populations are as differentiated from each other as *An. coluzzii* is from *An. gambiae s.s*., were collected in the same sites and at the same time points. The other populations PV and TD, are separated by a few hundred kilometers with no major geographic barrier between these regions of Cambodia. An admixture analysis supported the PCA analysis, breaking these specimens into at least four groups. The sympatric RK1 and RK2 populations are as distinct from one another as they are from the geographically distinct populations PV and TD. The RK2 population likely represents an emerging cryptic species or diverging clade within *An. minimus s.s*.. Further work will be needed to determine the degree to which RK2 is reproductively isolated from other *An. minimus s.s*. populations and how widespread this population is within Southeast Asia.

All four populations appear to be under selective pressure and may have undergone recent population expansions. These diverging populations of *An. minimus* each have unique signals of evolutionary selection. These signals are particularly prominent in the most differentiated population, RK2. Many of the signals identified in this study indicate a strong evolutionary response to insecticidal pressure in the GMS, this pressure is more likely due to heavy and long-term agricultural pesticide use rather than malaria vector control such as indoor residual spray or long-lasting insecticidal nets. Multiple mechanisms of insecticide resistance have been identified in other insects and in other disease vector mosquitoes in Cambodia^23,24,33^. The widespread use of pyrethroid, organophosphate, and carbamate insecticides in agriculture exacerbates the development of insecticide resistance in populations of many different species across the GMS^23^.

This study shows that populations of *An. minimus* in Cambodia are evolving, and this may be in the context of strong and sustained pressure from agricultural insecticide use. Each population in this study has unique signals of evolutionary selection associated with multiple detoxification pathways and may also be under different types of insecticidal pressure than mosquitoes in other parts of the world. For example, since *An. minimus* is a species that is able to exploit rice paddies and flooded agricultural fields as larval habitats, individual mosquitoes may experience insecticidal pressure as larvae, which might impact their susceptibility to insecticides as adults. The presence of these signals reveals a complicated landscape of potentially multiple insecticide-resistant vectors that may not be responsive to insecticides implemented as part of a malaria control campaign.

Current vector control tools in Cambodia and most of the GMS include the widespread distribution of long-lasting insecticidal nets (LLINs) and topical repellents. New control tools are needed in this region and in the context of outdoor, residual malaria transmission. The population structure within this single Southeast Asian vector species in a small country like Cambodia may indicate barriers to implementing vector control methods centered around genetic modification or sterile insect release. If populations of vectors are diverging with limited gene flow, despite being in the same place, genetic control based on any assumption of a single population of vectors would be unlikely to penetrate vector populations in this region.

This study expands the *Anopheles* species represented in whole genome population data. This resource of *An. minimus* whole genome data will be a starting point to explore *Anopheles* genome variation in important transmission contexts such as Southeast Asia. This study is the foundation for further delving into the population structure and history of *An. minimus*, which acts as an important malaria vector across all of Southeast Asia.

Examining the population structure in a single species over its range and through time will help us to better understand the potential risk associated with multiple insecticide-resistant populations of vectors and how best to approach managing and monitoring insecticide resistance. *An. minimus* in Cambodia includes an adaptive, resilient, and diverse set of populations that can thrive in human-created habitats and potentially contribute to epidemics of drug-resistant malaria as we work toward malaria elimination. It is crucial that we appreciate the evolutionary history and ecology of this important and widespread malaria vector.

## Materials and methods

### Collection methods

*An. minimus* female mosquitoes were selected from several previous field collections in Cambodia with the goal of including a subsample of *An. minimus* specimens from different field sites and time points for population genomic analysis. These anophelines were collected using CDC light traps, human landing collections, cow-baited tents, and barrier fences from five different locations in Cambodia. Collections in Thmar Da took place in 2010, while collections in two sites in Preah Vihear and Ratanakiri provinces took place monthly from February 2014 to January 2015 and in four quarterly collections during 2016. These five sites had enough *An. minimus* individuals to include in population genomic analyses.

Collection locations included Thmar Da, in Western Cambodia on the Thai/Cambodian border (Thmar Da) and in the North in Preah Vihear province (Chean Mok pagoda and Preah Kleang village), and in the Northeast Ratanakiri (Sayas and Chamkar San villages)^34,35^. Multiple *Anopheles* species were collected in each of these studies, including the *An. minimus A* specimens that have been included in this study. GPS coordinates for each collection site are available in the sample metadata (Supplementary Data 1 and 5).

### DNA extraction and identification

Specimens were morphologically identified immediately after collection using keys from^36,37^. These were stored individually in 1.5 ml tubes with silica gel desiccant. DNA was extracted from the head and thorax of collected mosquitoes using either a CTAB extraction method or in Nextec plates. Individual mosquitoes were molecularly identified using Sanger sequencing of the rDNA ITS2 region using primers ITS2A and ITS2B from^38^ and compared to voucher reference rDNA ITS2 sequences in NCBI.

### Whole-genome sequencing

Individual samples were sequenced at the Wellcome Trust Sanger Institute using whole-genome sequencing on the Illumina HiSeq 2000 platform. Paired-end multiplex libraries were prepared as per the manufacturer’s specifications, with DNA fragmented using Covaris Adaptive Focused Acoustics. Multiplexes comprised 12 tagged individual mosquitoes. Three lanes of sequencing were used for each multiplex to control for variation between sequencing runs. Sequencing and cluster generation was done using the manufacturer’s protocol for paired-end reads of 150 bp with insert sizes from 100 to 200 bp. The target coverage for each individual was 30X. To include some samples that had DNA concentration that was too low to run through the standard Illumina pipeline, we whole genome sequenced a set of specimens using a new low-input DNA sequencing pipeline as in^39,40^. This low-input sequencing pipeline uses enzymatic fragmentation and less than 10 nanograms of input DNA. In our sequence and population QC, these low-input samples did not appear to be of lower quality or influence the clustering of whole genome sequences in our PCA analysis. 144 individual *An. minimus* mosquitoes were sequenced using the standard Illumina pipeline, and 158 individuals were sequenced using the low-input pipeline.

### Sequence alignment

Illumina whole genome sequence reads were aligned to the *Anopheles* reference genome AminM1 using BWA version 0.7.15^41,42^. using bwa mem. A BAM file was constructed for each individual using merged alignments from multiple lanes. Duplicate reads were marked using Picard version 2.6.0^43^.

### Coverage

For each sample, the depth of coverage was computed at all genome positions. Samples were excluded if median coverage across all chromosomes was less than 10×, or if less than 80% of the reference genome was covered by at least 1X.

### Population outliers and anomaly detection

We used PCA to identify and exclude individual samples that were population outliers. SNPs were down-sampled to use 100,000 segregating non-singleton sites from autosomal contigs. PCA was computed using scikit-allel version 1.2.0. We iteratively identified and excluded any individual samples that were outliers along a single principal component. We then identified and excluded any individual samples or small sample groups that clustered together with other samples in a way that was not plausible, given metadata regarding their collection location. We initially performed a PCA with all of the 302 samples that passed our QC filters. We identified a group of 9 mosquitoes labeled as being collected in Preah Vihear province, but clustering with RK2. To be extremely conservative, the Preah Vihear samples that grouped with RK2 samples were removed from our final population-level assignment in case of potential mislabelling or plate mix-up or DNA contamination. Another 10 individual mosquitoes were excluded due to being isolated outliers in the PCA (not part of any cluster), driving PC2 and PC3. In all, we did not give a population assignment to 19 mosquitoes from our data set.

### ADMIXTURE analysis

We performed an analysis of population structure using ADMIXTURE version 1.3.0^39^. For each of the ten largest contigs in the reference genome, we identified biallelic SNPs with a minor allele frequency above 1% and then thinned these SNPs to select 50,000 per contig. We then ran ADMIXTURE on each contig separately for values of *K* from 2 to 6 inclusive. For each contig and value of *K* we repeated the ADMIXTURE analysis twice with different random seeds. All runs were performed with five-fold cross-validation to compute cross-validation error, which provides some guidance regarding the confidence of the fitted ancestry fractions.

### Quantification of population diversity and divergence

Diversity statistics Pairwise Fst, nucleotide diversity, Tajima’s D, and Watterson’s Theta were calculated in windows across the largest 40 contigs on the *An. minimus* AminM1 genome using scikit-allel version 1.3.3. and custom code in Python.

Nucleotide diversity (π), Watterson’s Theta (θ_W_), and Tajima’s D statistics were calculated over 4-fold degenerate sites on autosomal contigs larger than 2,000,000 bases. These 17 contigs represent 80% of the *Anopheles minimus* genome. These statistics were calculated as an average over sliding windows with 100 bootstrap replicates over samples. Populations were downsampled to the size of the smallest population RK2 (*n* = 28) for these calculations.

### Quantification of signals of selection

We used pairwise Fst in 1000 SNP windows to scan across the *Anopheles minimus* genome to look for any signals of increased differentiation, which might indicate evolutionary selection. This scan was performed for contigs larger than 2 Megabases, these contigs represent 86% of the *An. minimus* genome. Signals that were represented by at least five 1000 SNP windows above two times the genome-wide average were recorded and ranked by the highest Fst value at the peak of each selection signal.

Genes potentially associated with these signals were identified by going to the location of the peak of each signal on the *An. minimus* genome (AminM1) using the Genome Browser tool in VEuPathDB Vectorbase^28^ and walking in both directions from the location signal to see which annotated genes were present in that location. Gene IDs and gene functional predictions were recorded.

### Variants in known insecticide-resistance-associated genes

We report in this study variants at known insecticide resistance-associated alleles. Variants occurring at a frequency of more than 2% in at least one of the four populations are reported in the known insecticide-resistance-associated genes *Ace1*, *Rdl*, *KDR*, and *Gste2* (Supplementary Data 2).

### Reporting summary

Further information on research design is available in the Nature Portfolio Reporting Summary linked to this article.

## Supplementary information

Supplementary materials

Description of Additional Supplementary Files

Supplementary data 1-5

Reporting Summary

## Data Availability

These genome and SNP data are available at https://www.malariagen.net/resource/35. These data are hosted in Google Cloud. For more information about downloading data, please see the data download guide at https://malariagen.github.io/vector-data/amin1/intro.html. For more information about accessing data in the cloud, please see the cloud access guide at https://malariagen.github.io/vector-data/amin1/download.html. These data are also publicly available on Vectorbase:^28^ https://vectorbase.org/vectorbase/app/record/dataset/DS_dc54305595.

## References

[1] Van Bortel W (2010). Malaria transmission and vector behaviour in a forested malaria focus in central Vietnam and the implications for vector control. Malar. J..

[2] Durnez L (2013). Outdoor malaria transmission in forested villages of Cambodia. Malar. J..

[3] Hawkes FM (2019). Vector compositions change across forested to deforested ecotones in emerging areas of zoonotic malaria transmission in Malaysia. Sci. Rep..

[4] Trung HD (2005). Behavioural heterogeneity of *Anopheles* species in ecologically different localities in Southeast Asia: a challenge for vector control. Trop. Med. Int. Health.

[5] Van Bortel W (2004). Eco-ethological heterogeneity of the members of the *Anopheles minimus* complex (Diptera: Culicidae) in Southeast Asia and its consequences for vector control. J. Med. Entomol..

[6] Dev V, Manguin S (2016). Biology, distribution and control of *Anopheles* (Cellia) *minimus* in the context of malaria transmission in northeastern India. Parasites Vectors.

[7] Vantaux A (2021). *Anopheles* ecology, genetics and malaria transmission in northern Cambodia. Sci. Rep..

[8] Harbach RE, Gingrich JB, Pang LW (1987). Some entomological observations on malaria transmission in a remote village in northwestern Thailand. J. Am. Mosq. Control Assoc..

[9] Somboon P, Suwonkerd W, Lines JD (1994). Susceptibility of Thai zoophilic Anophelines and suspected malaria vectors to local strains of human malaria parasites. Southeast Asian J. Trop. Med Public Health.

[10] Amaratunga, C. et al. Dihydroartemisinin & piperaquine resistance in *Plasmodium falciparum* malaria in Cambodia: a multisite prospective cohort study. *Lancet Infectious Dis.* **16**, P357–365 (2016). 10.1016/S1473-3099(15)00487-9 PMC4792715 26774243

[11] Miotto O (2013). Multiple populations of artemisinin-resistant *Plasmodium falciparum* in Cambodia. Nat. Genet..

[12] Takala-Harrison S (2015). Independent Emergence of Artemisinin Resistance Mutations Among *Plasmodium falciparum* in Southeast Asia. J. Infect. Dis..

[13] Hamilton WL (2019). Evolution and expansion of multidrug-resistant malaria in southeast Asia: a genomic epidemiology study. Lancet Infect. Dis..

[14] St Laurent B (2015). Artemisinin-resistant *Plasmodium falciparum* clinical isolates can infect diverse mosquito vectors of Southeast Asia and Africa. Nat. Commun..

[15] Poolphol P (2017). Natural *Plasmodium vivax* infections in *Anopheles* mosquitoes in a malaria endemic area of northeastern Thailand. Parasitol. Res..

[16] Cui L (2012). Malaria in the Greater Mekong subregion: heterogeneity and complexity. Acta Trop..

[17] Sinka ME (2011). The dominant *Anopheles* vectors of human malaria in the Asia-Pacific region: occurrence data, distribution maps and bionomic precis. Parasites Vectors.

[18] Fontaine MC (2015). Mosquito genomics. Extensive introgression in a malaria vector species complex revealed by phylogenomics. Science.

[19] The *Anopheles gambiae* 1000 Genomes Consortium. Genetic diversity of the African malaria vector *Anopheles gambiae*. *Nature* **552**, 96–100 (2017). 10.1038/nature24995 PMC6026373 29186111

[20] WHO, *Anopheline Species Complexes in South and South-East Asia*. S. K. Subbarao, Ed., (World Health Organization 2007), pp. 102.

[21] Garros C, Harbach RE, Manguin S (2005). Systematics and biogeographical implications of the phylogenetic relationships between members of the funestus and minimus groups of Anopheles (Diptera: Culicidae). J. Med. Entomol..

[22] Sochantha T (2006). Insecticide‐treated bednets for the prevention of *Plasmodium falciparum* malaria in Cambodia: a cluster‐randomized trial. Tropical Med. Int. Health.

[23] Van Bortel W (2008). The insecticide resistance status of malaria vectors in the Mekong region. Malar. J..

[24] Marcombe S (2017). Insecticide resistance status of malaria vectors in Lao PDR. PloS ONE.

[25] Somboon P, Prapanthadara LA, Suwonkerd W (2003). Insecticide susceptibility tests of *Anopheles minimus* s.l., *Aedes aegypti*, *Aedes albopictus*, and *Culex quinquefasciatus* in northern Thailand. Southeast Asian J. Trop. Med. Public Health.

[26] van den Berg H, Velayudhan R, Yadav RS (2021). Management of insecticides for use in disease vector control: lessons from six countries in Asia and the Middle East. PLoS Negl. Trop. Dis..

[27] The Anopheles gambiae 1000 Genomes Consortium (2020). Genome variation and population structure among 1142 mosquitoes of the African malaria vector species *Anopheles gambiae* and *Anopheles coluzzii*. Genome Res..

[28] Giraldo-Calderon GI (2015). VectorBase: an updated bioinformatics resource for invertebrate vectors and other organisms related with human diseases. Nucleic Acids Res..

[29] Ranson H (2001). Identification of a novel class of insect glutathione S-transferases involved in resistance to DDT in the malaria vector *Anopheles gambiae*. Biochem. J..

[30] Zhou Y (2019). UDP-glycosyltransferase genes and their association and mutations associated with pyrethroid resistance in *Anopheles sinensis* (Diptera: Culicidae). Malar. J..

[31] Cao X, Gulati M, Jiang H (2017). Serine protease-related proteins in the malaria mosquito, *Anopheles gambiae*. Insect Biochem. Mol. Biol..

[32] Marcombe S (2020). Malaria and dengue mosquito vectors from Lao PDR show a lack of the rdl mutant allele responsible for cyclodiene insecticide resistance. J. Med. Entomol..

[33] Boyer, S. et al. Resistance of *Aedes aegypti* populations to deltamethrin, permethrine and temephos in Cambodia. *Am. J. Trop. Med. Hyg.* **99**, 265 (2018). 10.1177/1010539517753876 29502428

[34] St Laurent B (2016). Cow-baited tents are highly effective in sampling diverse *Anopheles* malaria vectors in Cambodia. Malar. J..

[35] St. Laurent, B. Clinically informed mosquito sampling in Cambodia—a year-long survey of diverse and abundant malaria vectors in three provinces. Unpublished.

[36] Peyton, E. Scanlon, J. E. Malikul, V. Imvitaya, S. “Illustrated key to the female *Anopheles* mosquitoes of Thailiand,” (Army Medical Component AFRIMS APO San Francisco 96346, 1966).

[37] Rattanarithikul R, Harrison BA, Panthusiri P, Peyton EL, Coleman RE (2006). Illustrated keys to the mosquitoes of Thailand III. Genera Aedeomyia, Ficalbia, Mimomyia, Hodgesia, Coquillettidia, Mansonia, and Uranotaenia. Southeast Asian J. Trop. Med. Public Health.

[38] Beebe NW, Saul A (1995). Discrimination of all members of the Anopheles punctulatus complex by polymerase chain reaction-restriction fragment length polymorphism analysis. Am. J. Trop. Med. Hyg..

[39] Alexander DH, Novembre J, Lange K (2009). Fast model-based estimation of ancestry in unrelated individuals. Genome Res..

[40] Ellis P (2021). Reliable detection of somatic mutations in solid tissues by laser-capture microdissection and low-input DNA sequencing. Nat. Protoc..

[41] Li H, Durbin R (2009). Fast and accurate short read alignment with Burrows-Wheeler transform. Bioinformatics.

[42] Li H, Durbin R (2010). Fast and accurate long-read alignment with Burrows-Wheeler transform. Bioinformatics.

[43] Broad_Institute, Picard Toolkit. *GitHub Repository*. http://broadinstitute.github.io/picard/ (2019).

