# Supplementary material for: Population genomics reveal distinct and diverging populations of An. minimus in Cambodia
Source: Commun Biol. 2022 Nov 28;5:1308. doi: 10.1038/s42003-022-04259-y (PMC9705317; doi:10.1038/s42003-022-04259-y)

Supplementary materials for:

Population genomics reveal distinct and diverging populations of *An. minimus* in Cambodia – a widespread malaria vector in Southeast Asia

Brandyce St. Laurent, Nick Harding, Nick Deason, Kolthida Oy, Chea Sok Loeun, Men Sary, Rous Sunly, Sen Nhep, Eleanor Drury, Kirk Rockett, Siv Sovannaroth, Sonia Goncalves, Alistair Miles, Dominic Kwiatkowski

Supplementary Figures:

Supplementary Figure 1 – ADMIXTURE analysis

Supplementary Figure 2 - Nucleotide diversity across the largest 14 AminM1 contigs for four Cambodian populations

Supplementary Figure 3 - Watterson's Theta across the largest 14 AminM1 contigs for four Cambodian populations

Supplementary Figure 4 - Tajima's D across the largest 14 AminM1 contigs for four Cambodian populations

Supplementary Figure 5.1 - 5.6 - *An. minimus* Fst scan population comparisons
These tables include Fst scans in 1000 SNP windows across the largest 18 AminM1 contigs for each of 6 population comparisons.

Supplementary Figure 6.A- 6.DD – Fst plots at 1000 SNP, 200 SNP, and individual SNP scales plotted over annoted genes within 100kb of each top selection signal

Supplementary Figure 7 – Histograms of mean coverage of samples by population

Supplementary Figure 8.1 - 8.6 – Raw Fst (single SNP values) over the largest 10 AminM1 contigs for each population comparison

Supplementary Data:

Supplementary Data 1 - Sample metadata
This table includes unique sample identifiers and collection metadata for each individual female mosquito included in this study.

Supplementary Data 2 - IR SNP variants
SNP variants occurring in over 2% in any within known insecticide-resistance associated genes Ace1, Rdl, KDR, and GSTe2, population are reported here.

Supplementary Data 3 - Population Fst
Pariwise average Fst calculations in 20Kb windows for the four populations over the five largest contigs are reported.

Supplementary Data 4 – Min contig locations
Summary of largest 40 contigs used for diversity statistics and other calculations in this study, including which *Anopheles* genomic element and AgamP4 chromosome equivalent arm they lie on.

Supplementary Data 5 – Sampling summary
A summary of timepoints and sites where individual *An. minimus* samples were collected.

Supplementary Figure 1 – ADMIXTURE Analysis

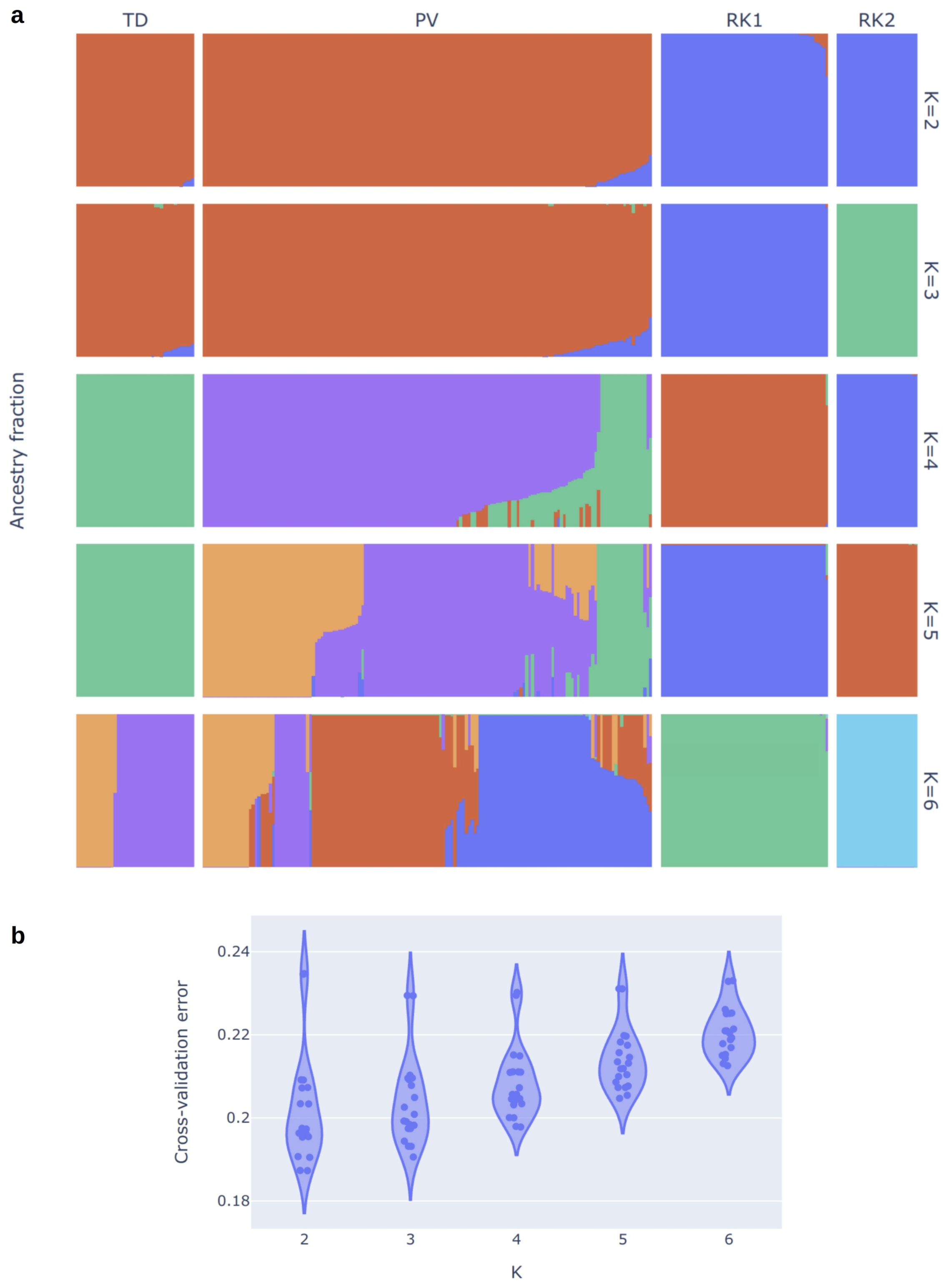

A) Results of admixture analysis using the largest contig KB663610 for values of K=2 to K=6. Plots show the fraction of ancestry for 283 individuals. Individuals have been grouped into four cohorts based on sampling location and results from PCA analysis. TD for Thmar Da, PV for Preah Vihear, RK1 for Ratanakiri population 1, and RK2 for Ratanakiri population 2 corresponding to population designations derived from PCA clustering of the same samples.

B) Results from 5-fold cross validation analysis from admixture run on the 10 largest AminM1 contigs with 2 replicates per contig using different random seeds.

Supplementary Figure 2 - Nucleotide diversity calculated in 50kb windows across the largest 14 AminM1 contigs for four Cambodian populations

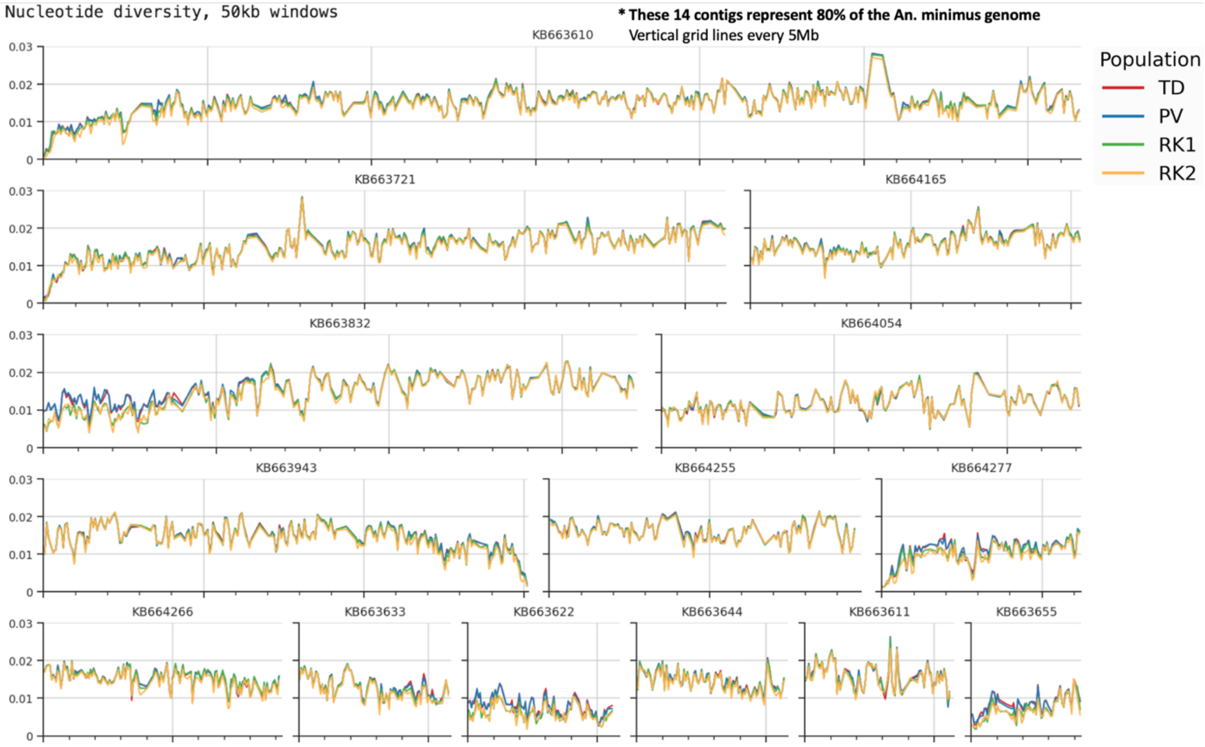

Supplementary Figure 3 - Watterson's Theta calculated in 50kb windows across the largest 14 AminM1 contigs for four Cambodian populations

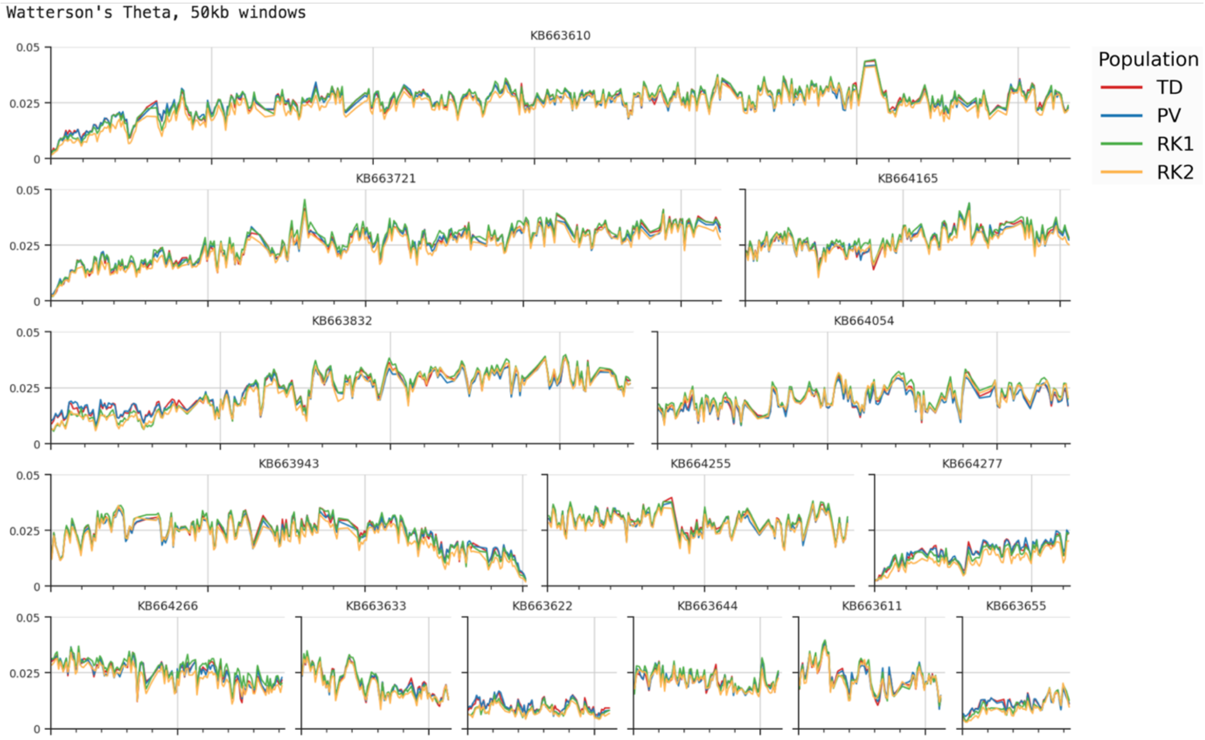

Supplementary Figure 4 - Tajima's D calculated in 50kb windows across the largest 14 AminM1 contigs for four Cambodian populations

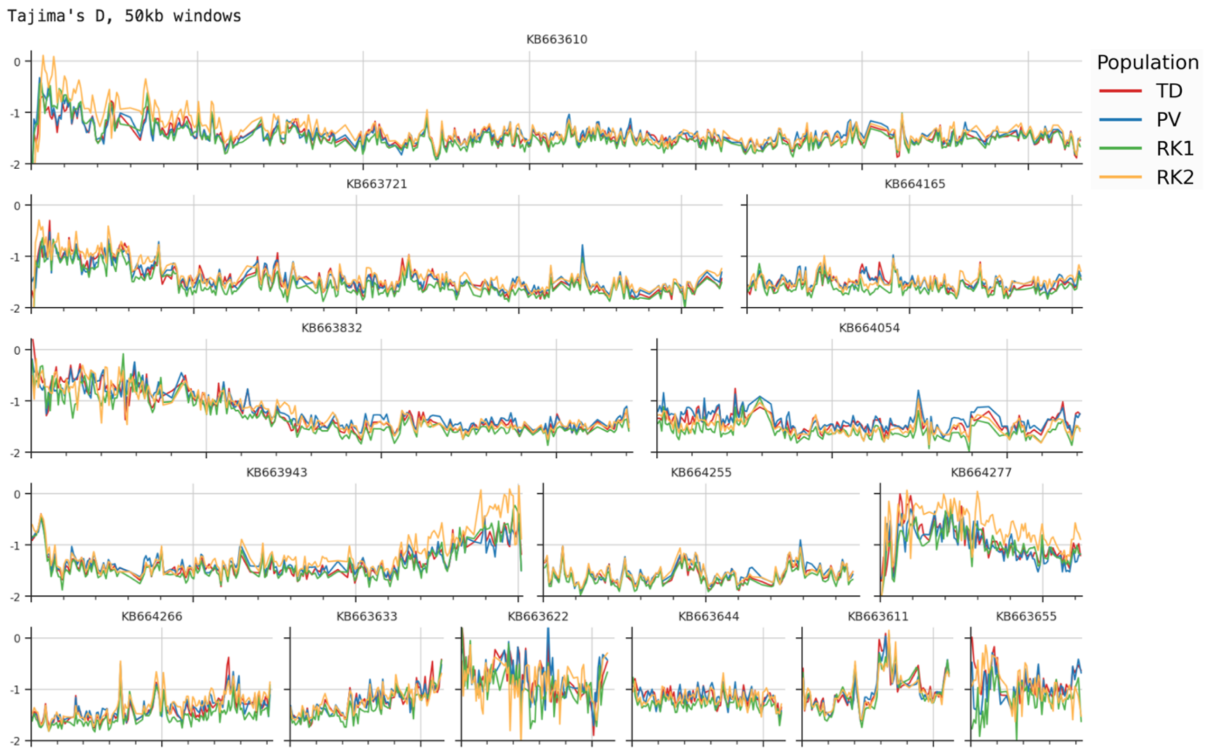

Supplementary Figure 5.1

TD vs. PV

Fst scans in 1000 SNP windows across the largest 18 AminM1 contigs

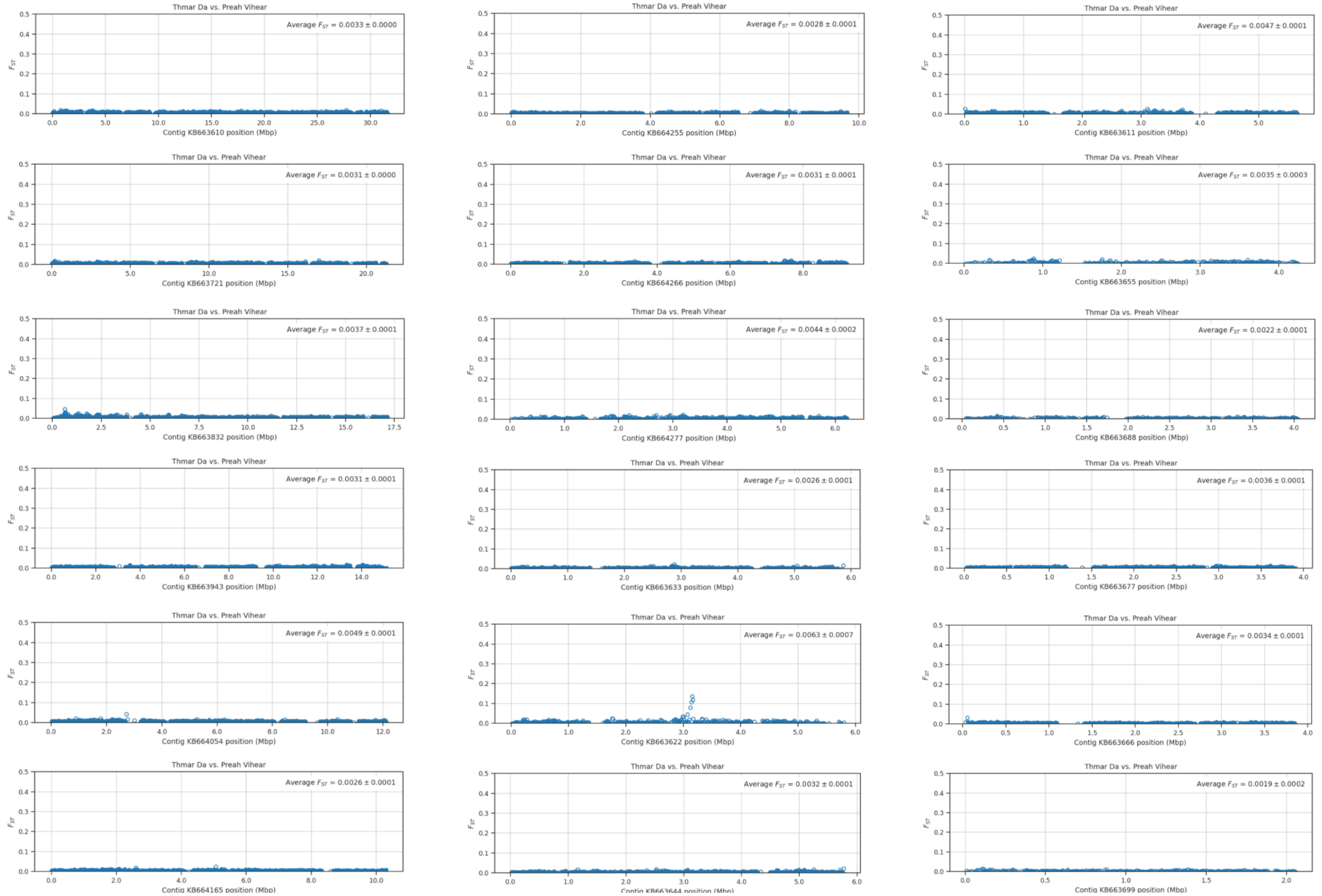

Supplementary Figure 5.2

TD vs. RK1

Fst scans in 1000 SNP windows across the largest 18 AminM1 contigs

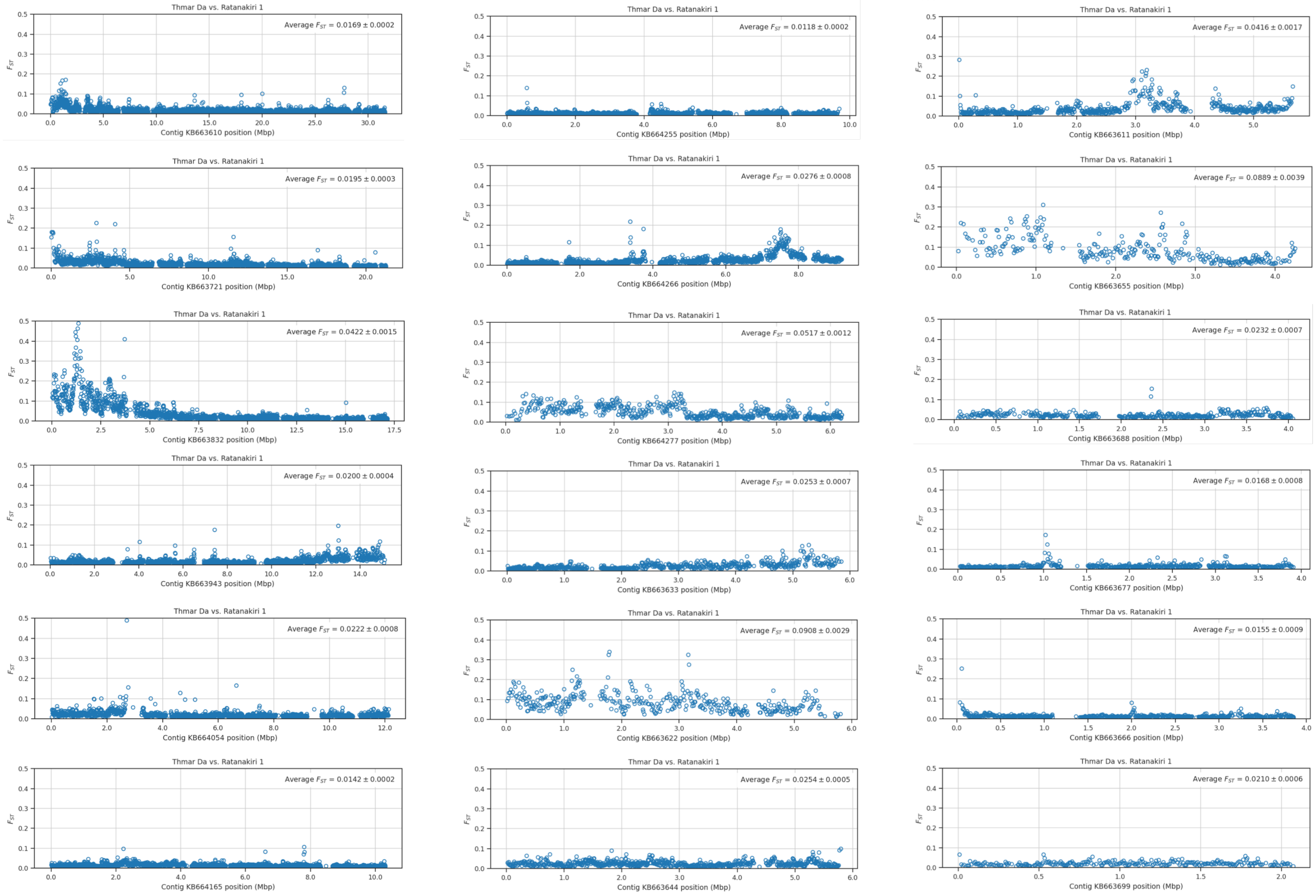

Supplementary Figure 5.3

TD vs. RK2

Fst scans in 1000 SNP windows across the largest 18 AminM1 contigs

*axes adjusted to accommodate Fst greater than 0.5

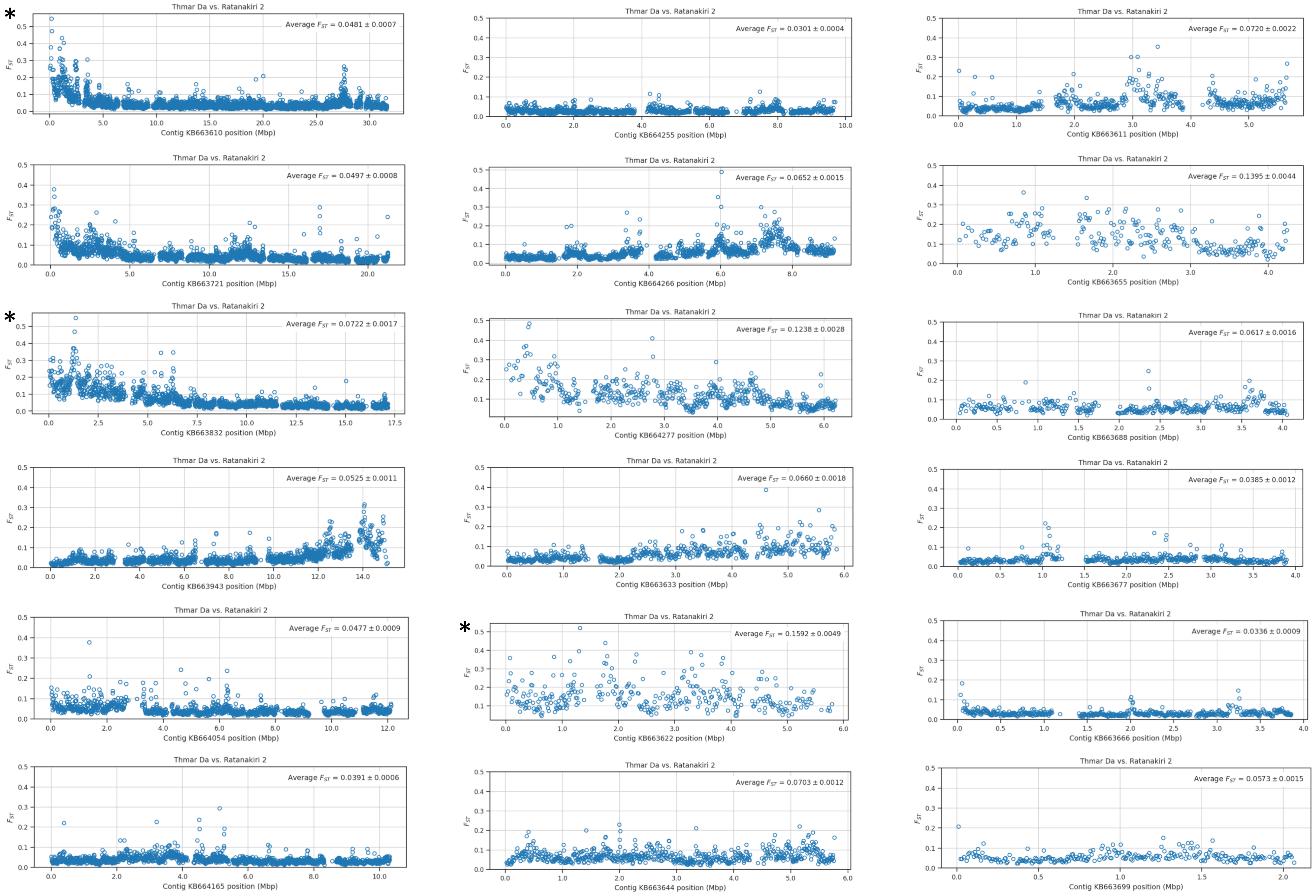

Supplementary Figure 5.4

PV vs. RK1

Fst scans in 1000 SNP windows across the largest 18 AminM1 contigs

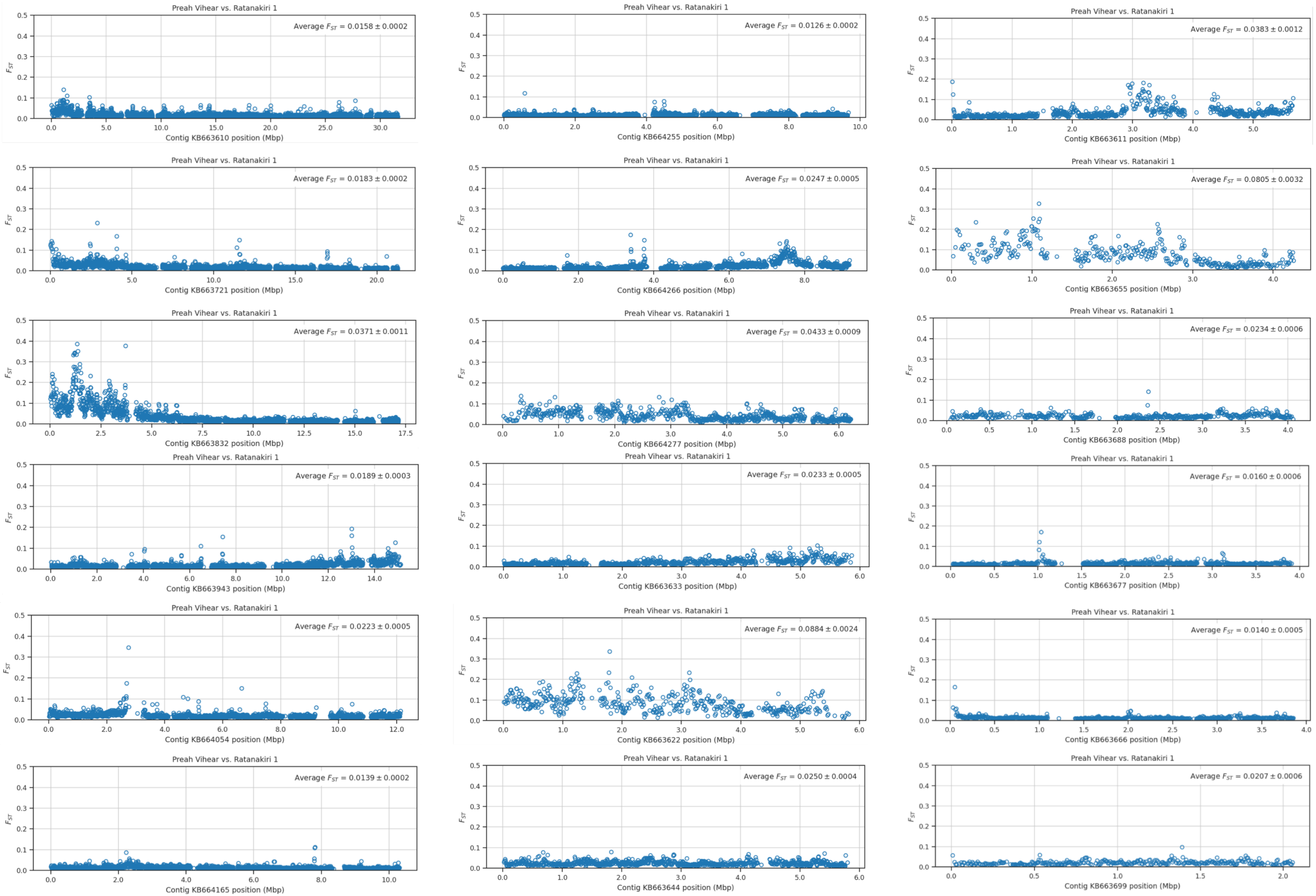

Supplementary Figure 5.5

PV vs. RK2

Fst scans in 1000 SNP windows across the largest 18 AminM1 contigs

*axes adjusted to accommodate Fst greater than 0.5

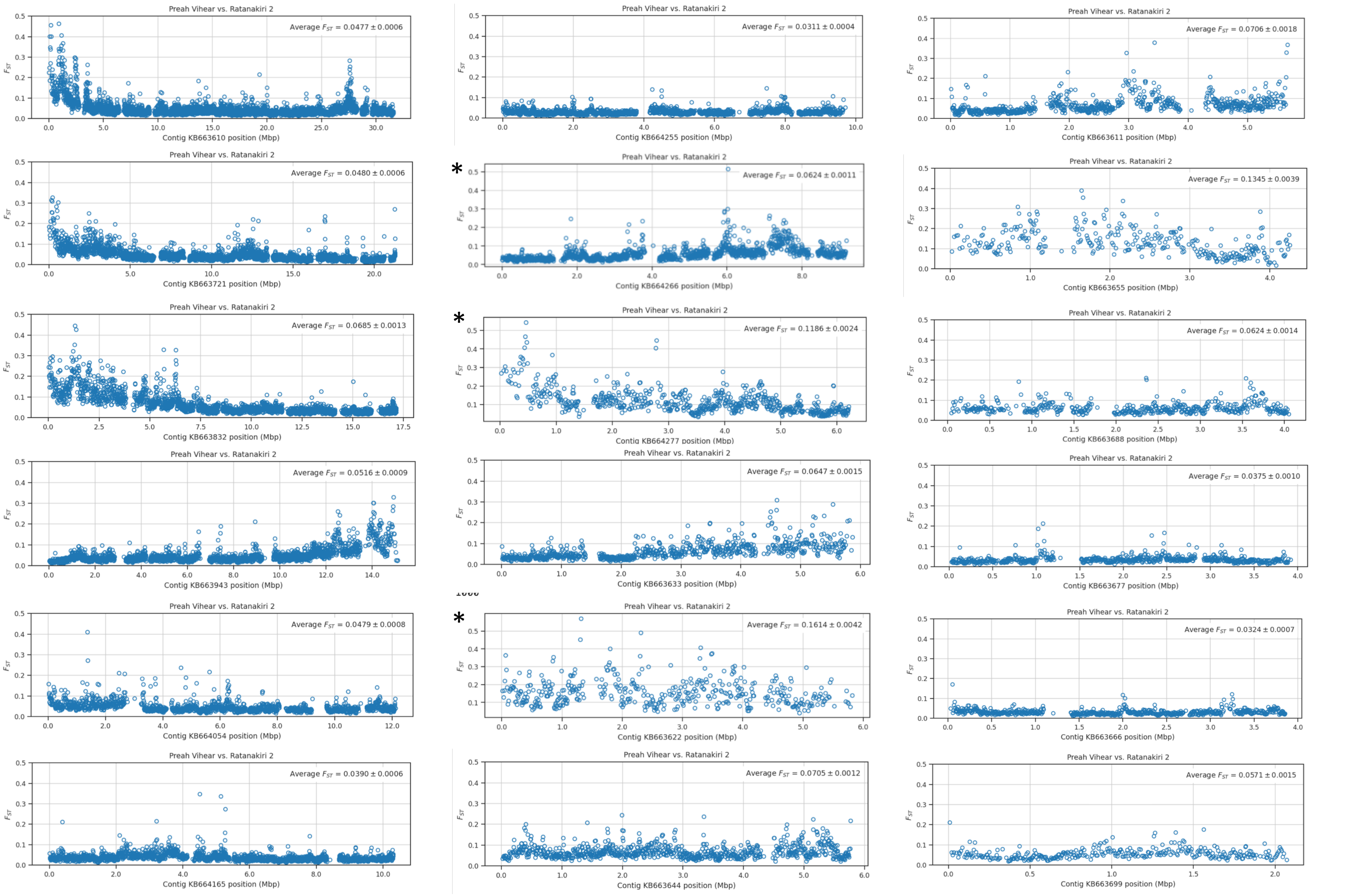

Supplementary Figure 5.6

RK1 vs. RK2

Fst scans in 1000 SNP windows across the largest 18 AminM1 contigs

*axes adjusted to accommodate Fst greater than 0.5

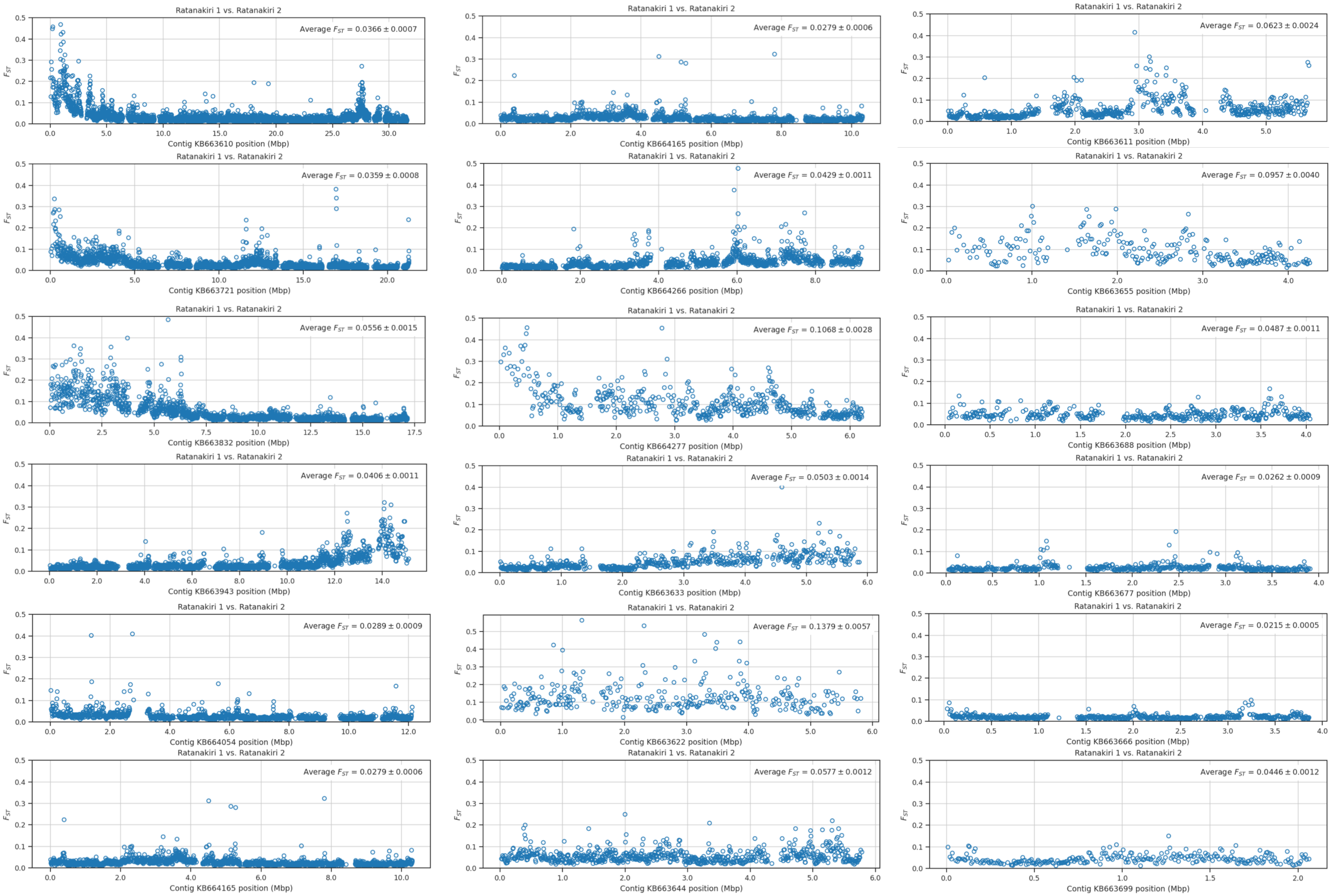

Supplementary Figure 6

Fst plots over selection signals

Guide to signals

Signal	Population Comparison	Contig	Signal Position (Mb)	Fst Value
A	RK1 v RK2	KB663622	1.3	0.54
B	RK1 v RK2	KB663622	2.3	0.52
C	RK1 v RK2	KB664266	6	0.49
D	RK1 v RK2	KB663832	5.7	0.49
E	RK1 v RK2	KB663610	2	0.48
F	PV v RK2	KB663622	1.25	0.54
G	PV v RK2	KB664277	0.4	0.53
H	PV v RK2	KB663610	0.1	0.53
I	PV v RK2	KB664266	6	0.51
J	PV v RK2	KB663622	2.2	0.5
K	PV v RK1	KB663832	1.4	0.39
L	PV v RK1	KB663832	3.7	0.38
M	PV v RK1	KB664054	2.6	0.34
N	PV v RK1	KB663622	1.8	0.33
O	PV v RK1	KB663655	1	0.33
P	TD v RK2	KB663610	0.1	0.55
Q	TD v RK2	KB663832	1.3	0.55
R	TD v RK2	KB663622	1.25	0.52
S	TD v RK2	KB664266	6	0.5
T	TD v RK2	KB664277	0.4	0.49
U	TD v RK1	KB663832	1.25	0.5
V	TD v RK1	KB664054	2.6	0.49
W	TD v RK1	KB663832	3.75	0.4
X	TD v RK1	KB663622	1.8	0.34
Y	TD v RK1	KB663622	3.1	0.32
Z	TD v PV	KB663622	3.1	0.125
AA	TD v PV	KB663832	0.6	0.049
BB	TD v PV	KB664054	2.7	0.042
CC	TD v PV	KB663666	0.5	0.032
DD	TD v PV	KB663832	1.35	0.027

Supplementary Figure 6

Signal A

1000 SNP windows

200 SNP windows

Raw Fst values (single SNPs)

Genes

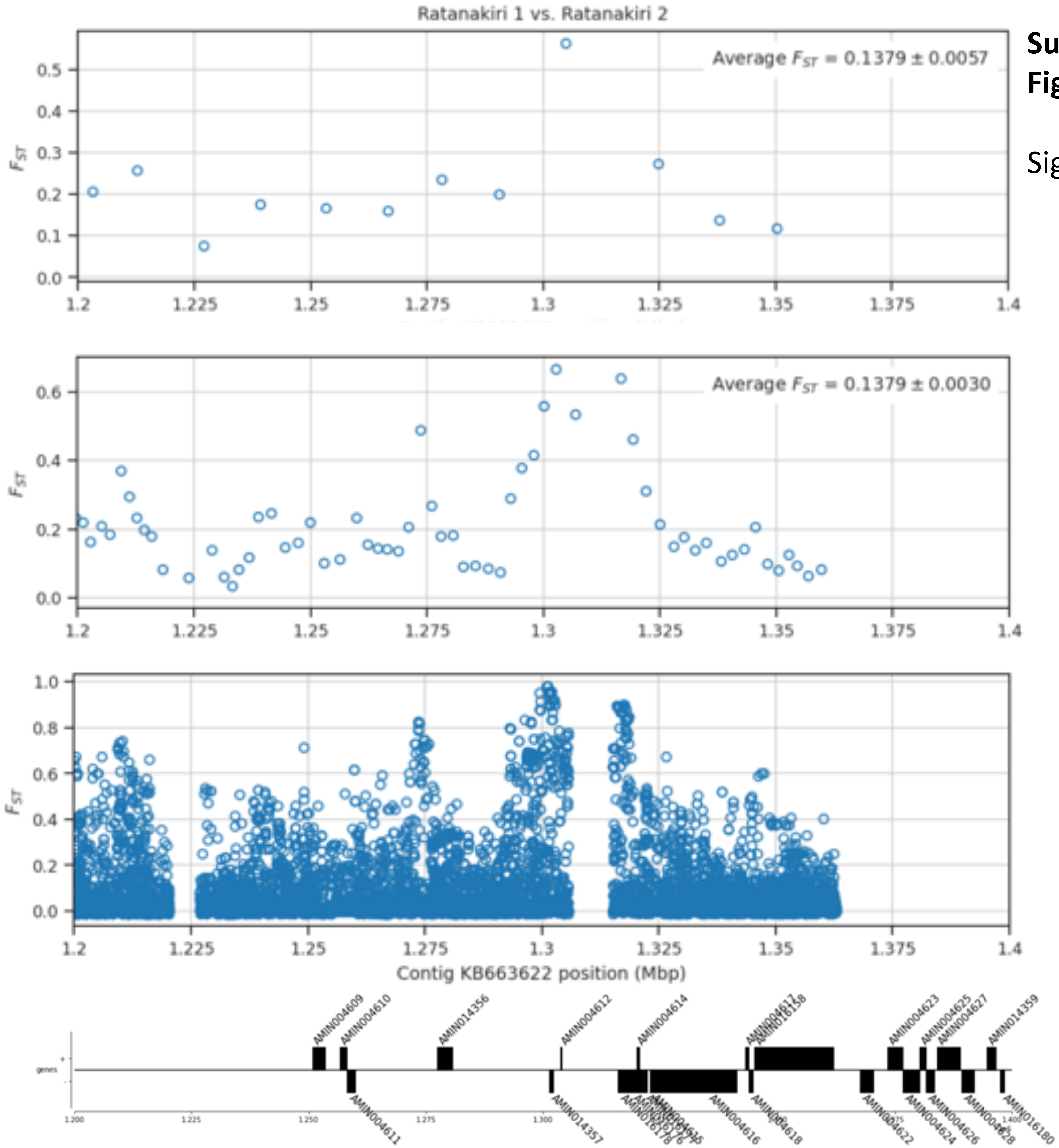

Supplementary Figure 6

Signal B

1000 SNP windows

200 SNP windows

Raw Fst values (single SNPs)

Genes

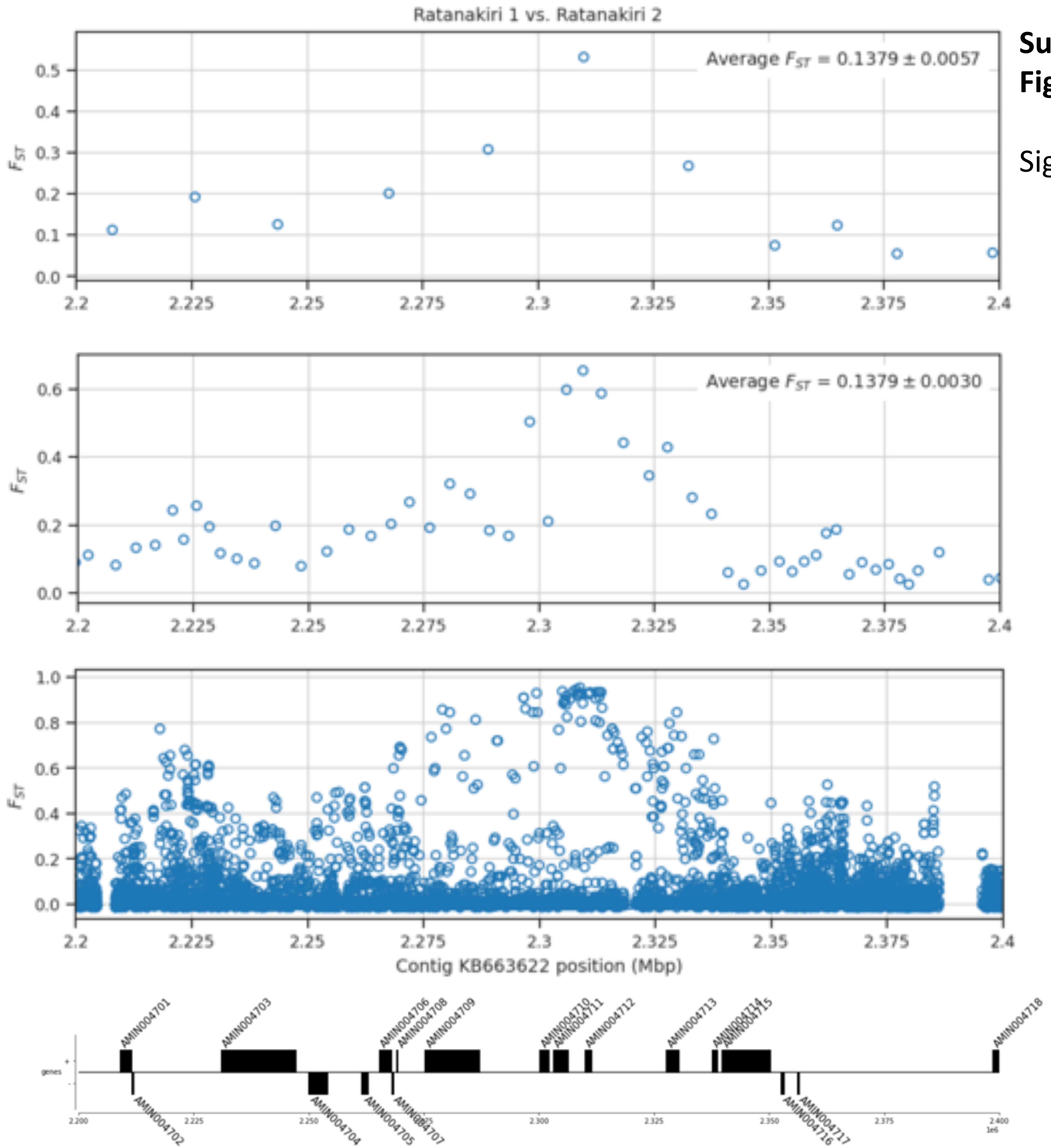

Supplementary Figure 6

Signal C

1000 SNP windows

200 SNP windows

Raw Fst values (single SNPs)

Genes

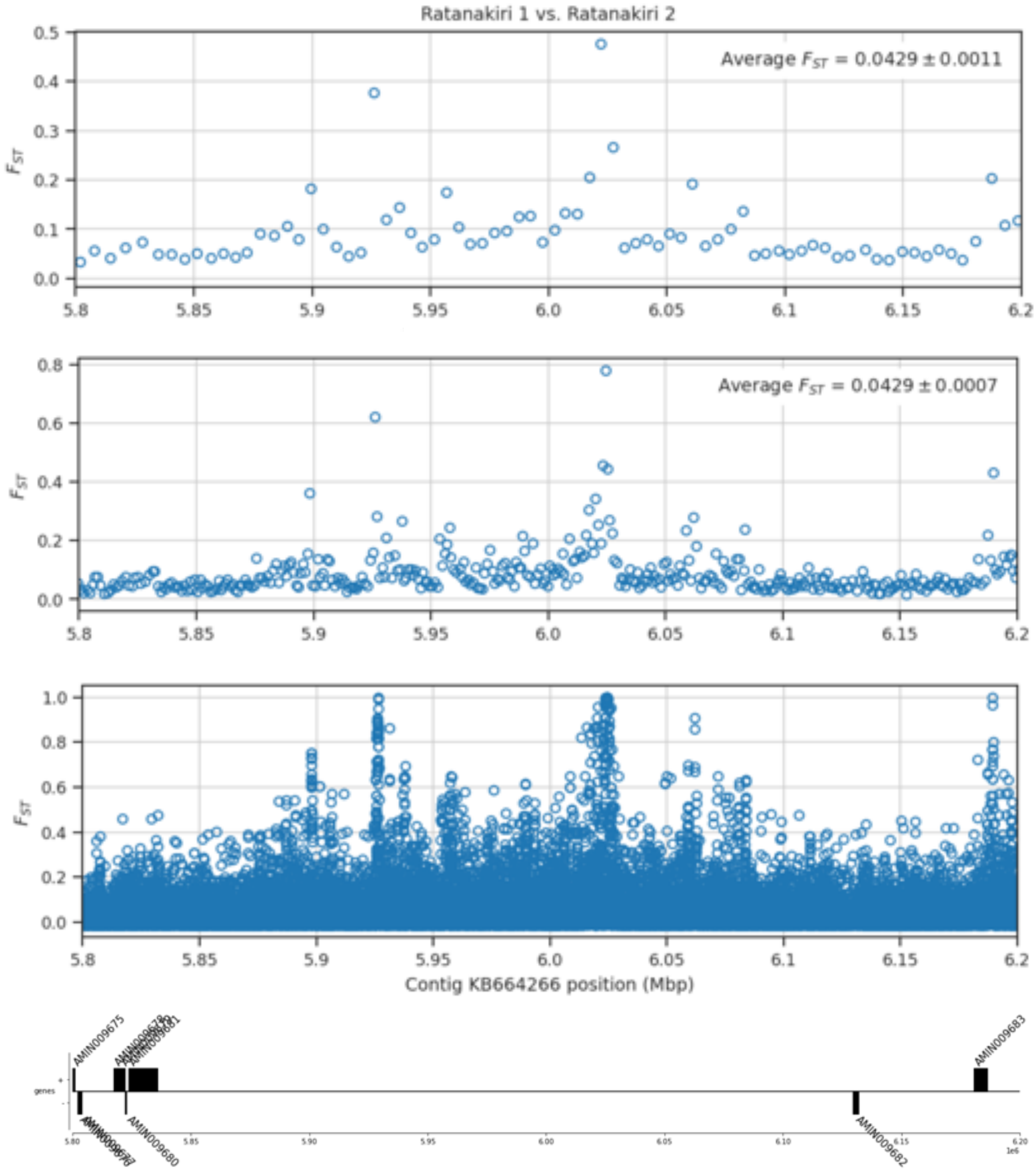

Supplementary Figure 6

Signal D

1000 SNP windows

200 SNP windows

Raw Fst values (single SNPs)

Genes

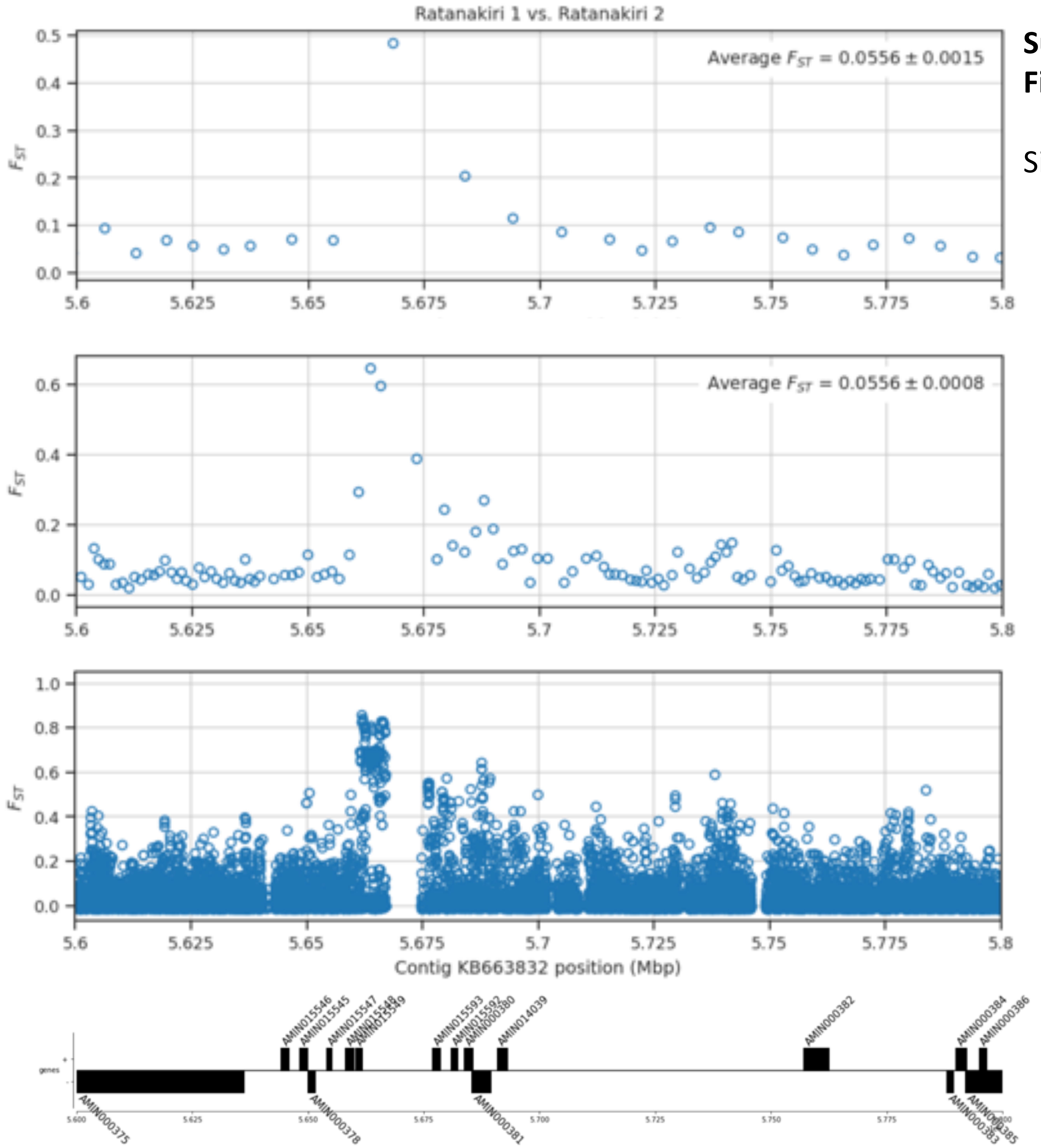

Supplementary Figure 6

Signal E

1000 SNP windows

200 SNP windows

Raw Fst values (single SNPs)

Genes

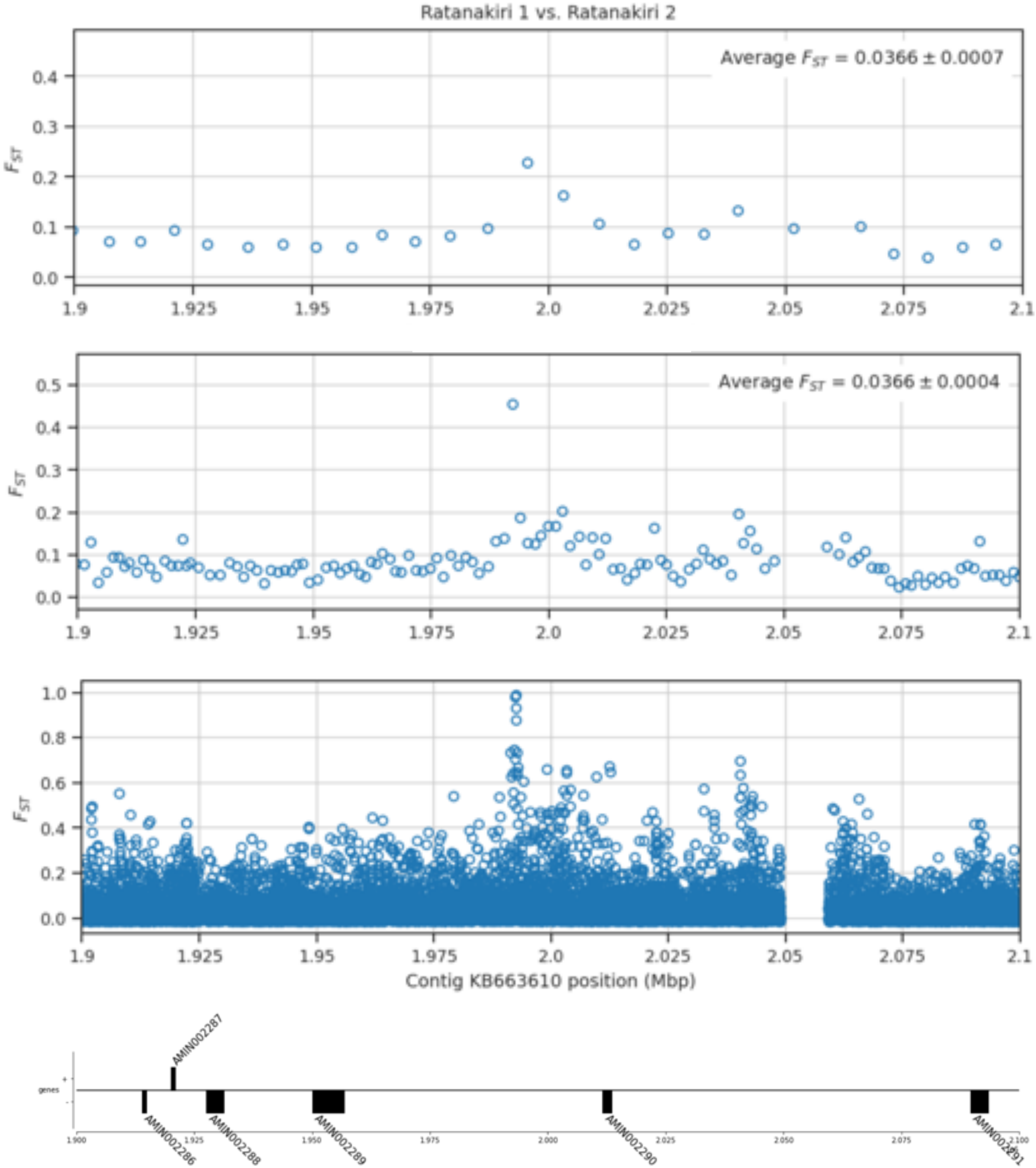

Supplementary Figure 6

Signal F

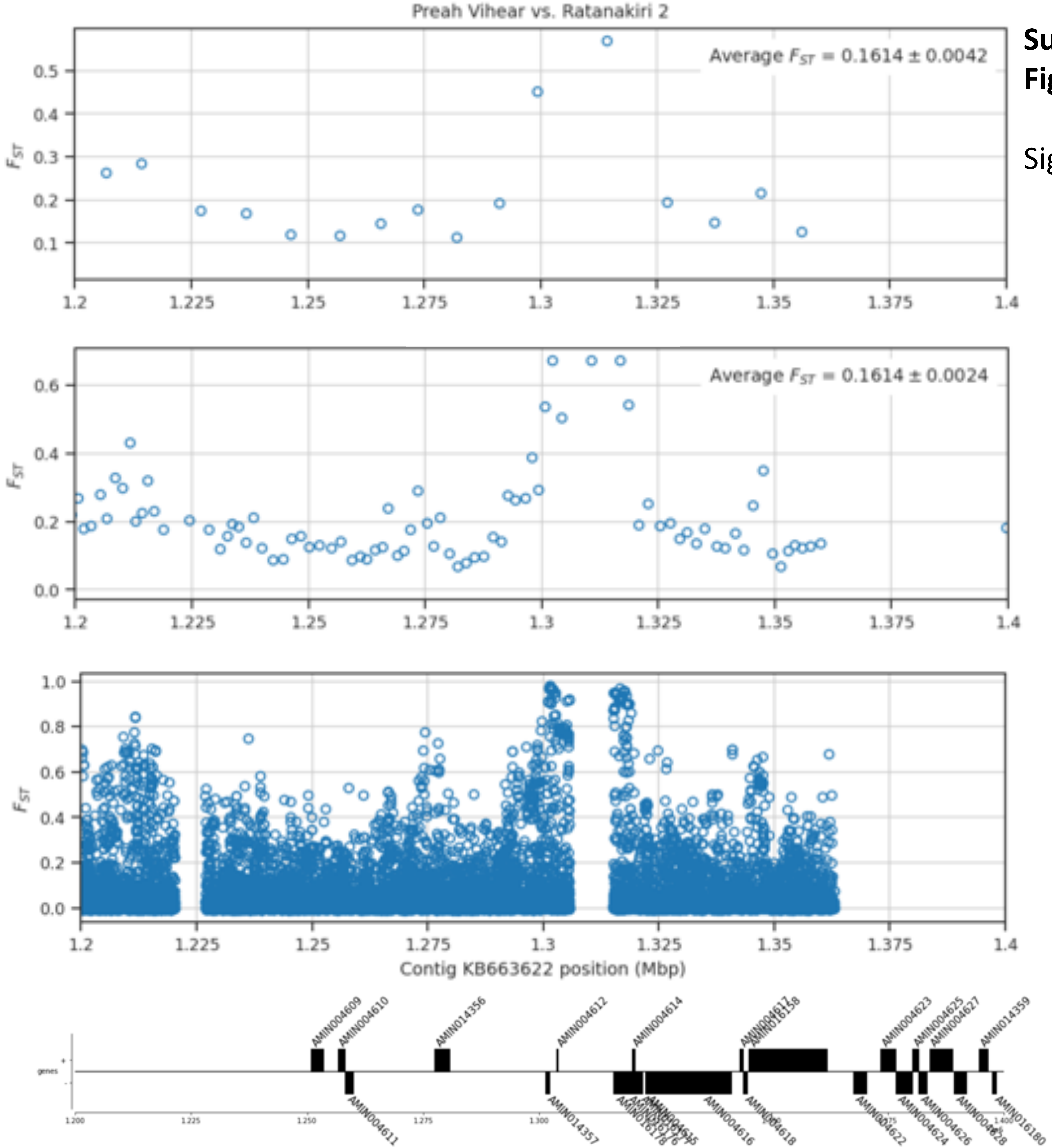

1000 SNP windows

200 SNP windows

Raw Fst values (single SNPs)

Genes

Supplementary Figure 6

Signal G

1000 SNP windows

200 SNP windows

Raw Fst values (single SNPs)

Genes

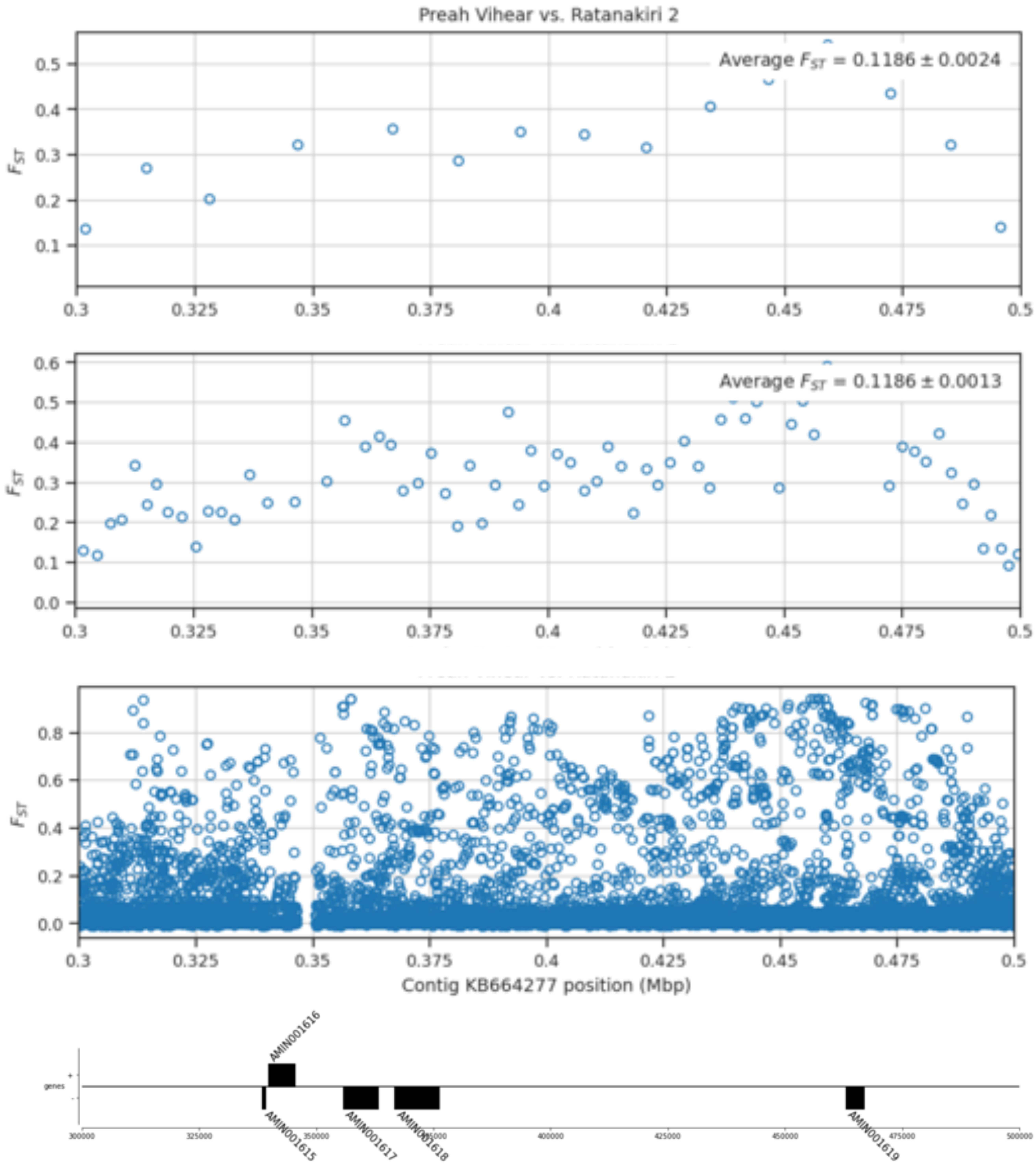

Supplementary Figure 6

Signal H

1000 SNP windows

200 SNP windows

Raw Fst values (single SNPs)

Genes

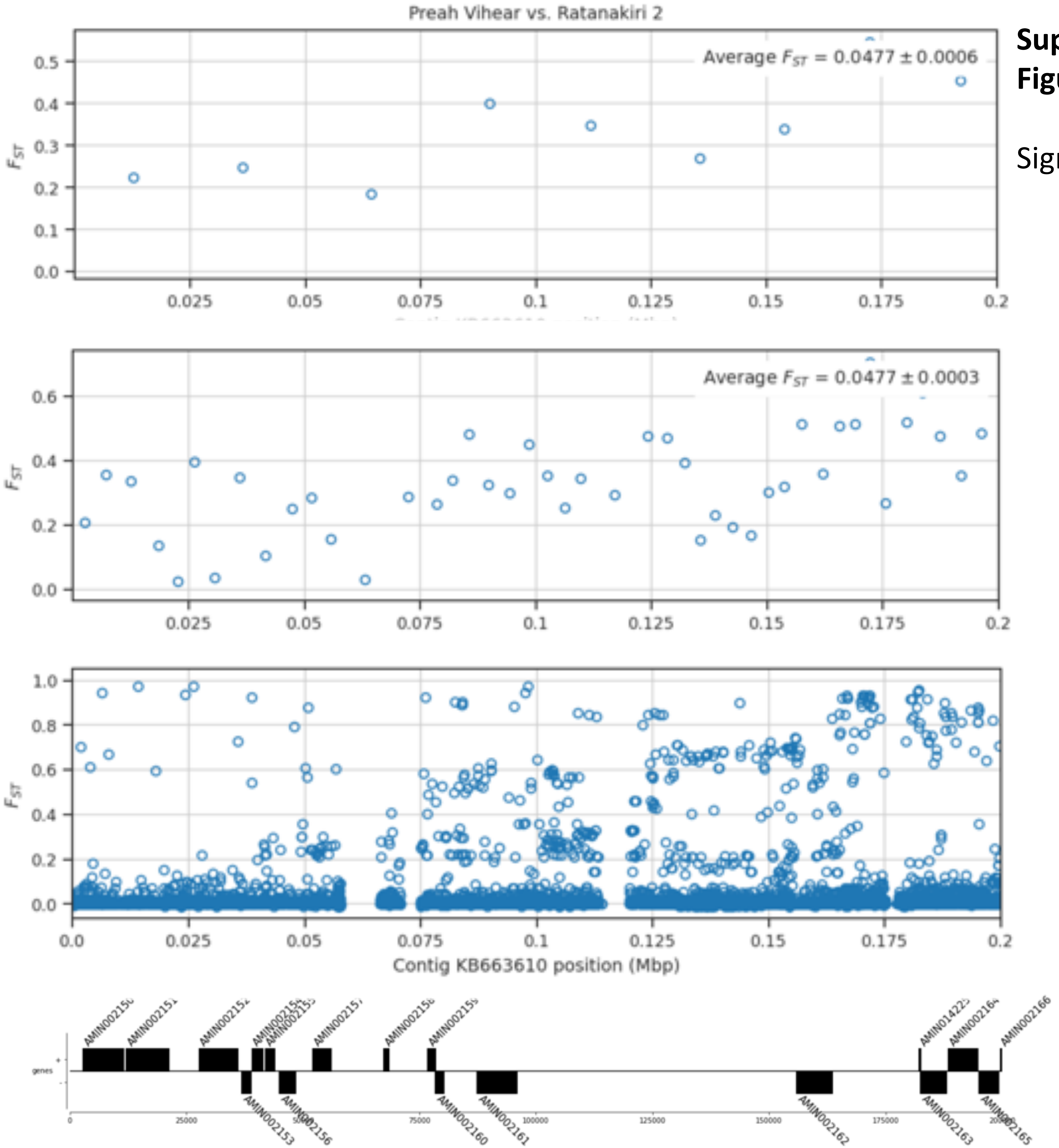

Supplementary Figure 6

Signal I

1000 SNP windows

200 SNP windows

Raw Fst values (single SNPs)

Genes

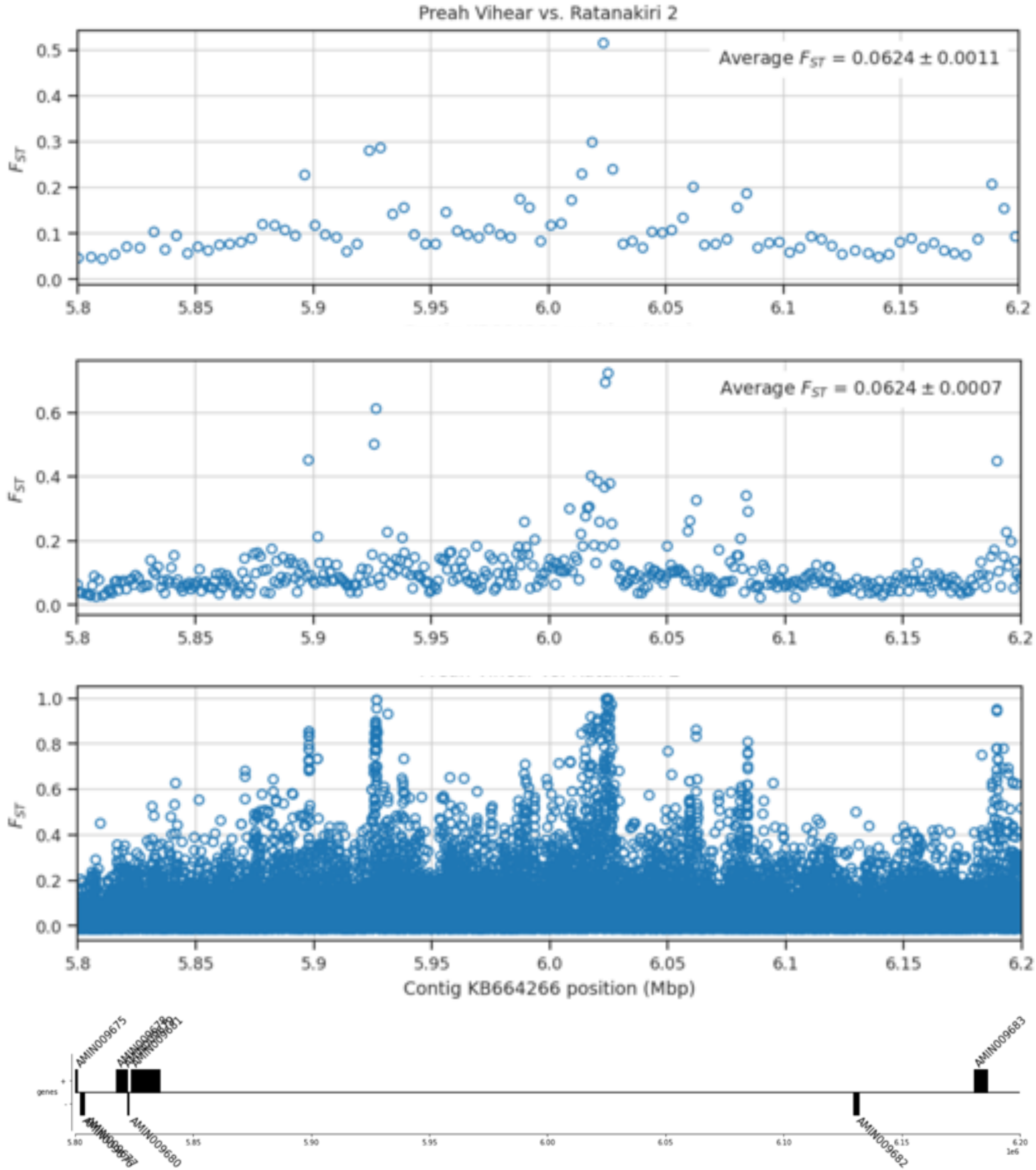

Supplementary Figure 6

Signal J

1000 SNP windows

200 SNP windows

Raw Fst values (single SNPs)

Genes

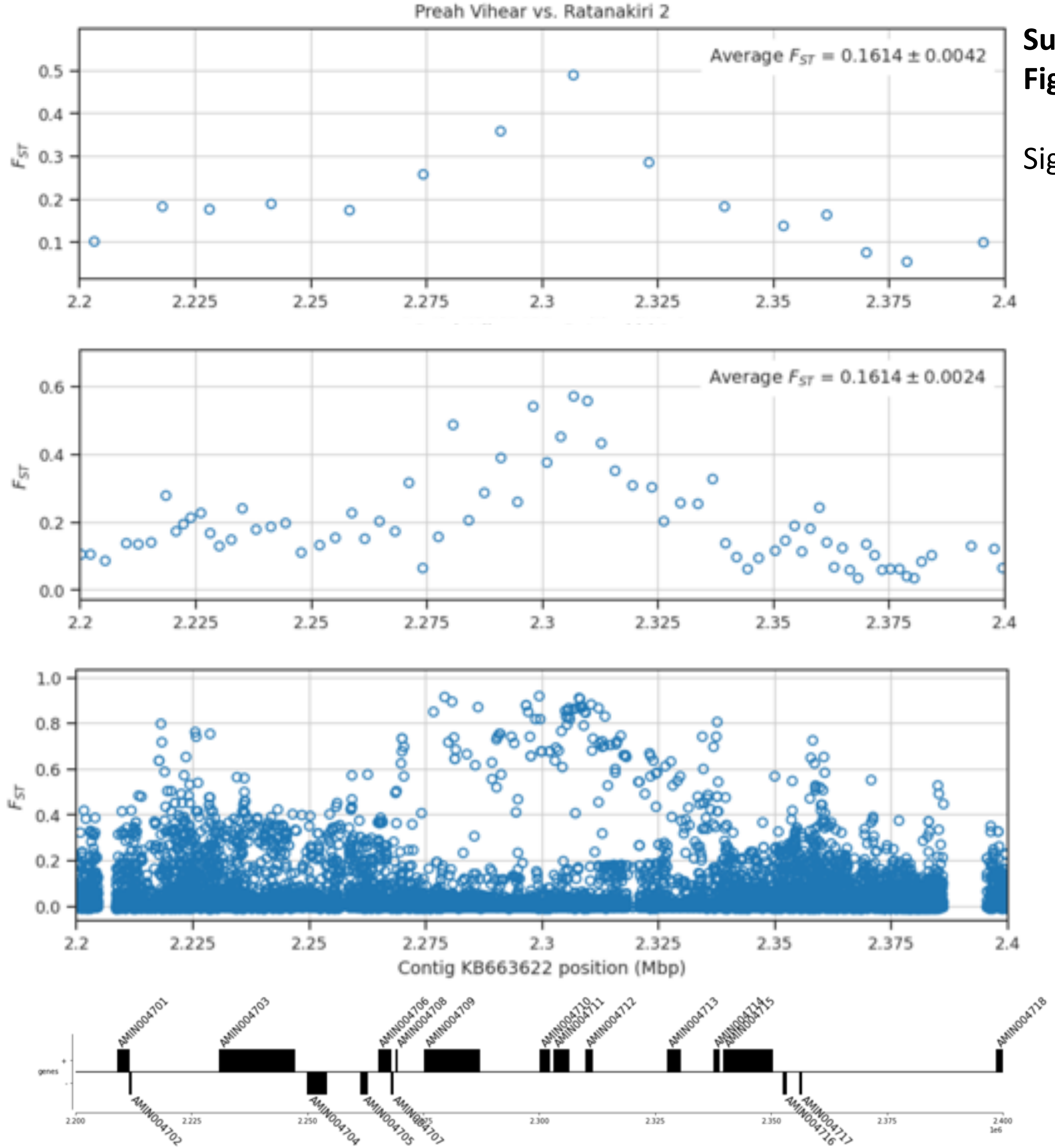

Supplementary Figure 6

Signal K

1000 SNP windows

200 SNP windows

Raw Fst values (single SNPs)

Genes

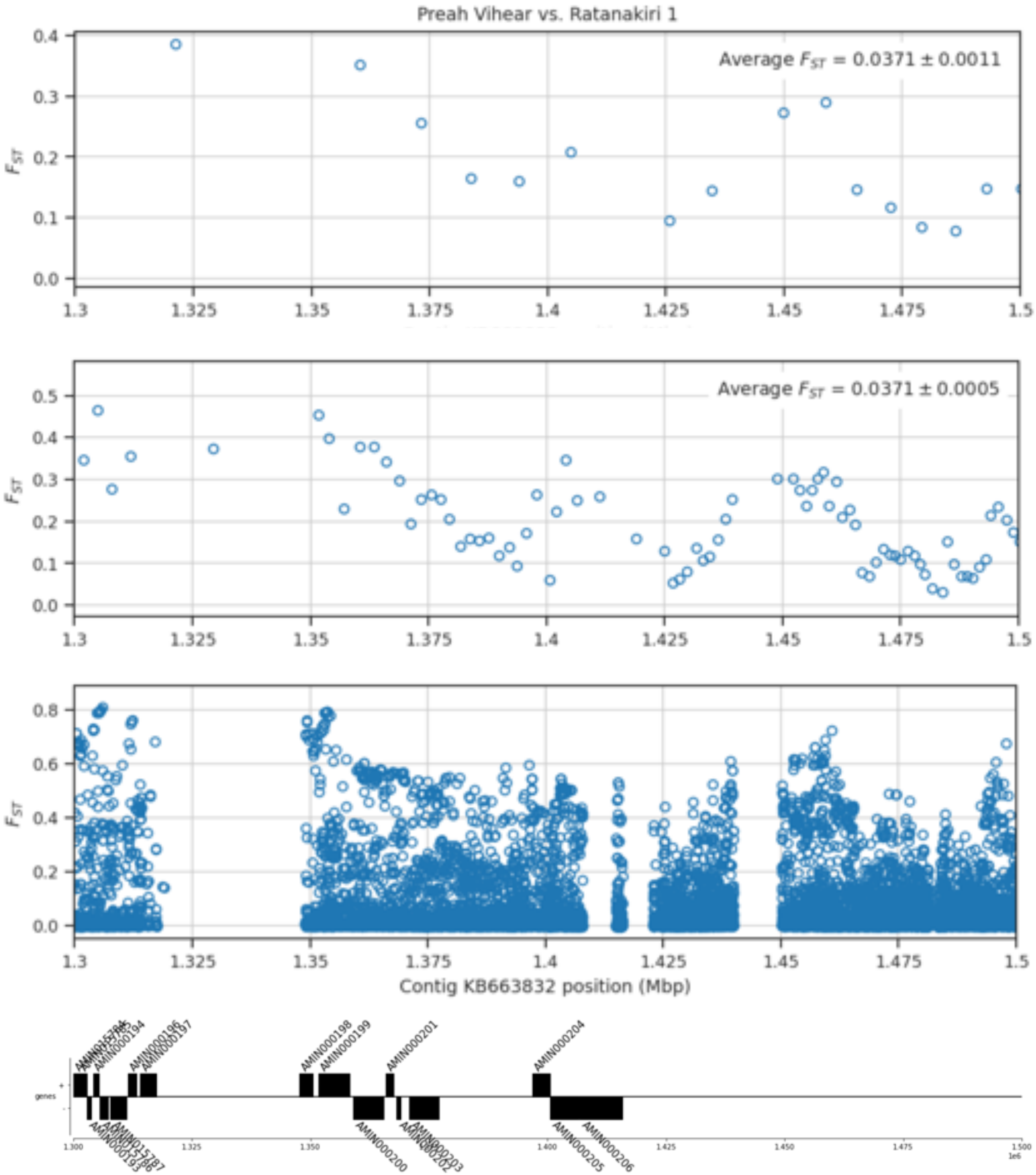

Supplementary Figure 6

Signal L

1000 SNP windows

200 SNP windows

Raw Fst values (single SNPs)

Genes

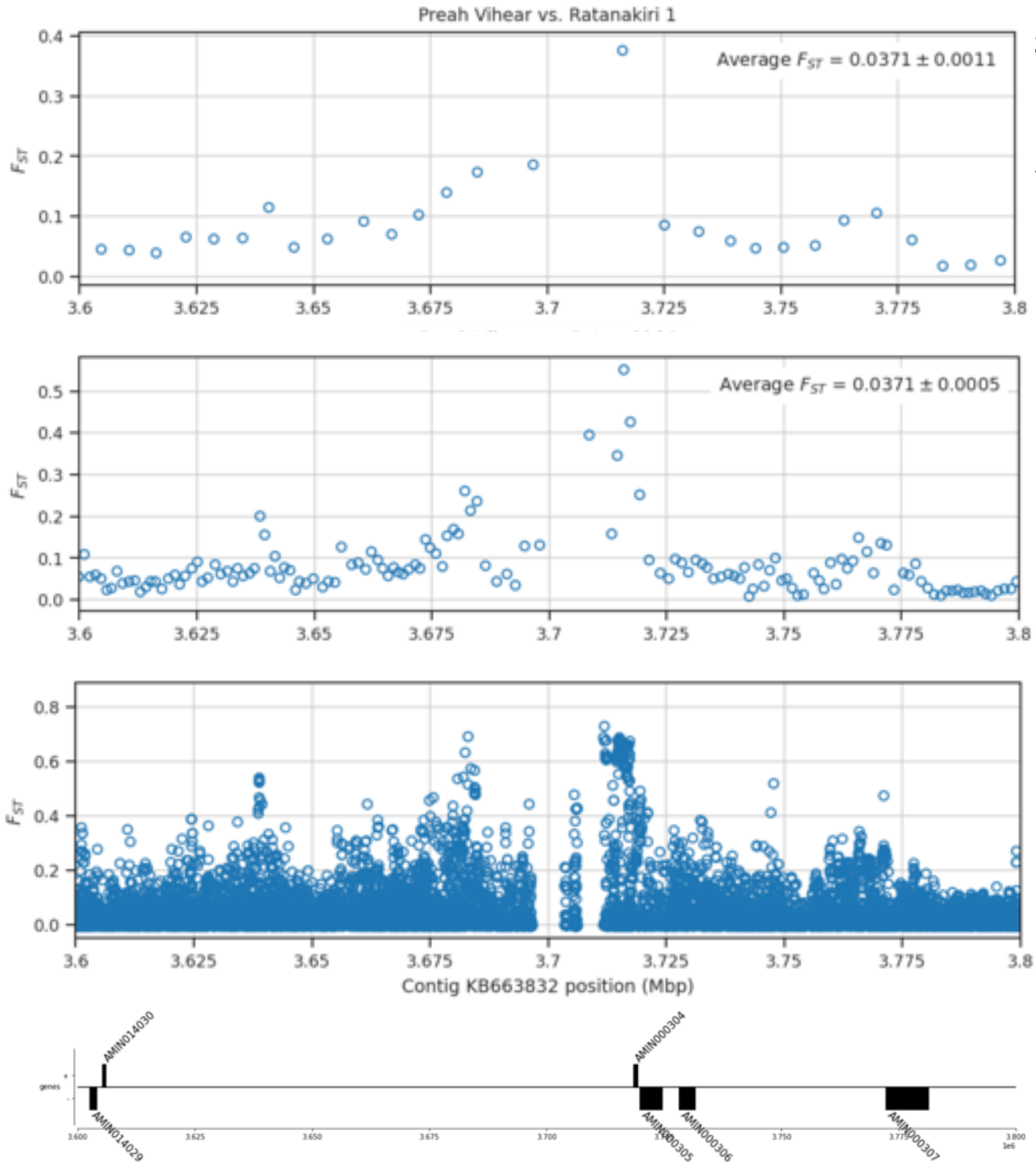

Supplementary Figure 6

Signal M

1000 SNP windows

200 SNP windows

Raw Fst values (single SNPs)

Genes

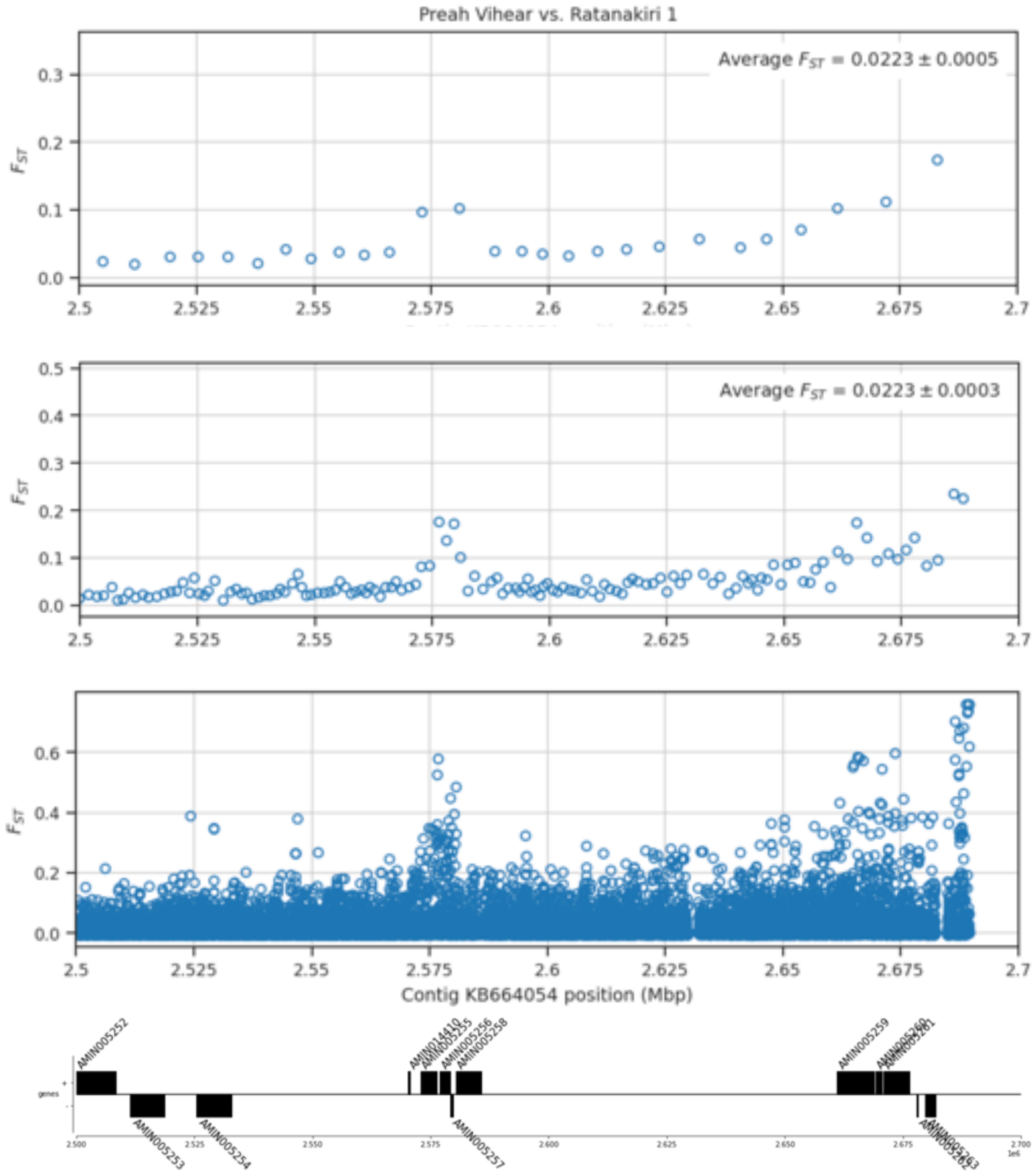

Supplementary Figure 6

Signal N

1000 SNP windows

200 SNP windows

Raw Fst values (single SNPs)

Genes

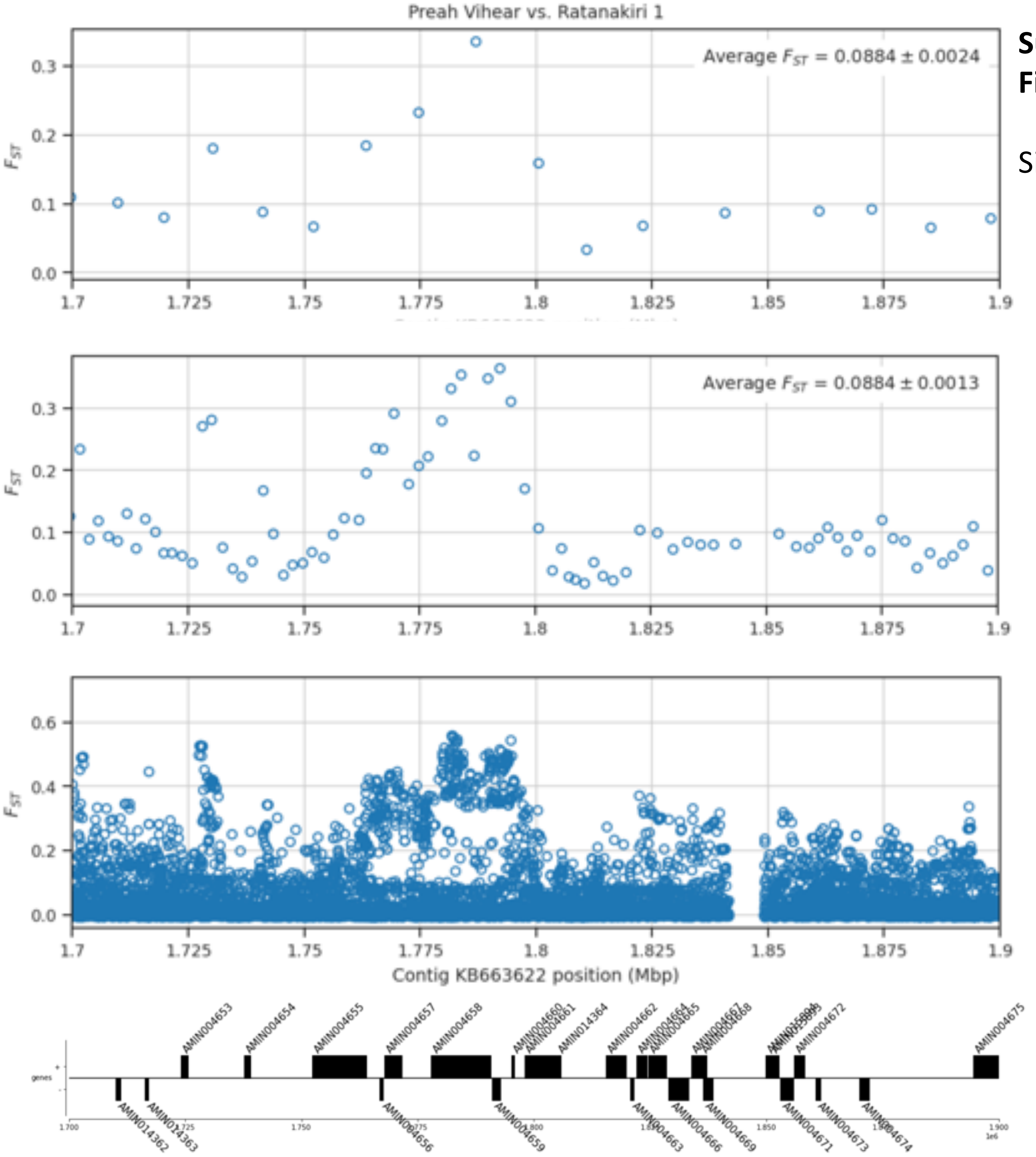

Supplementary Figure 6

Signal O

1000 SNP windows

200 SNP windows

Raw Fst values (single SNPs)

Genes

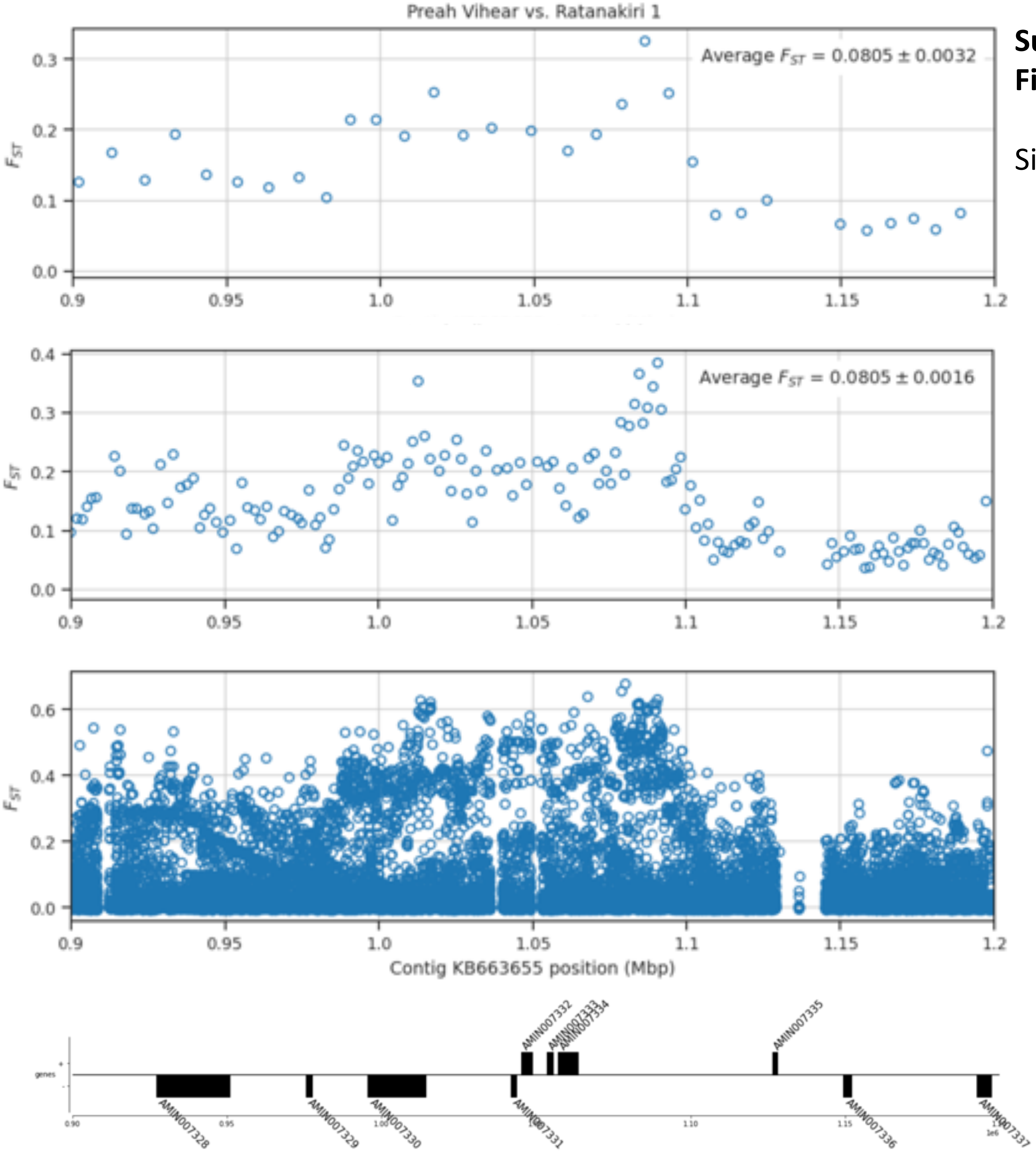

Supplementary Figure 6

Signal P

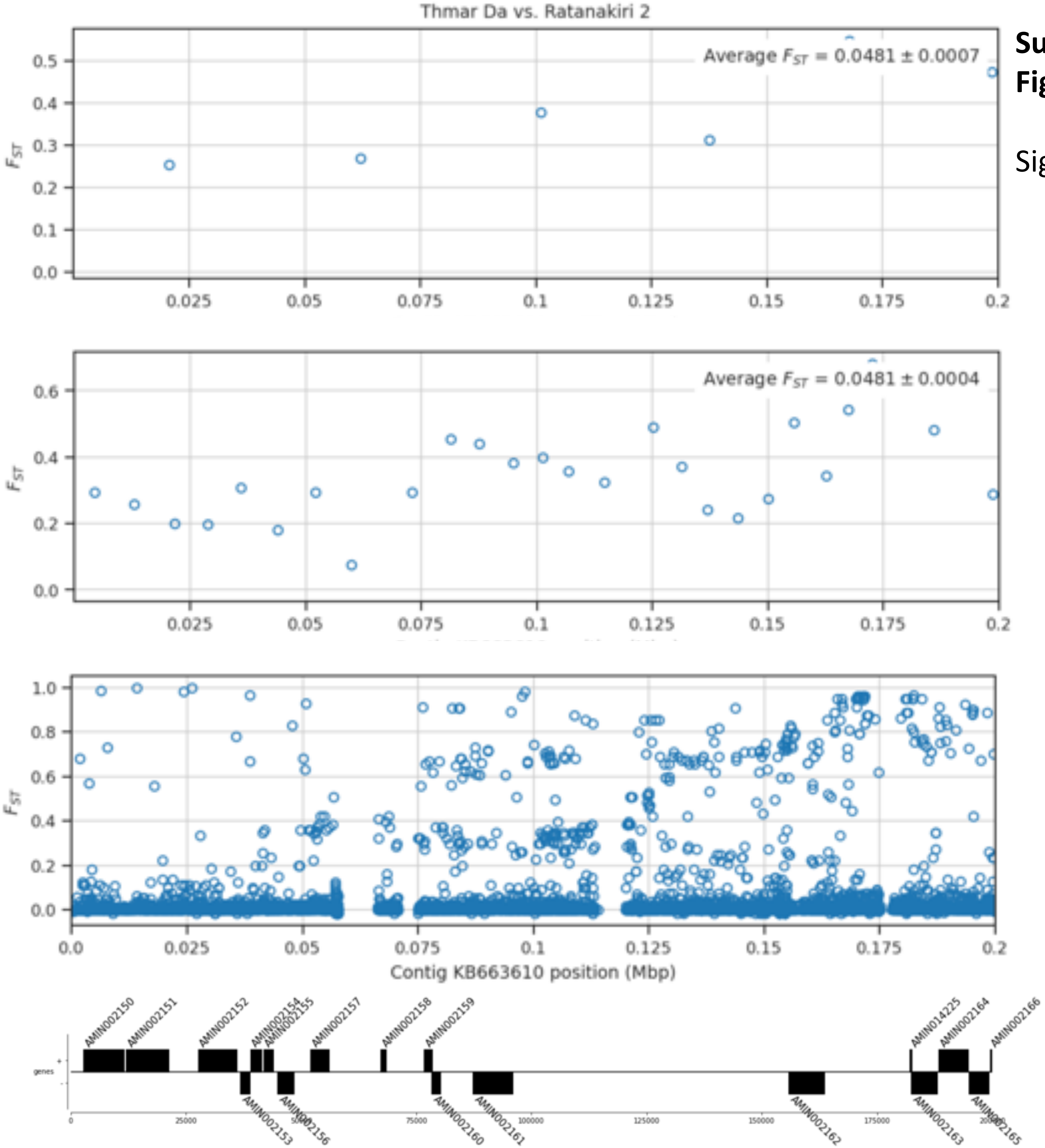

1000 SNP windows

200 SNP windows

Raw Fst values (single SNPs)

Genes

Supplementary Figure 6

Signal Q

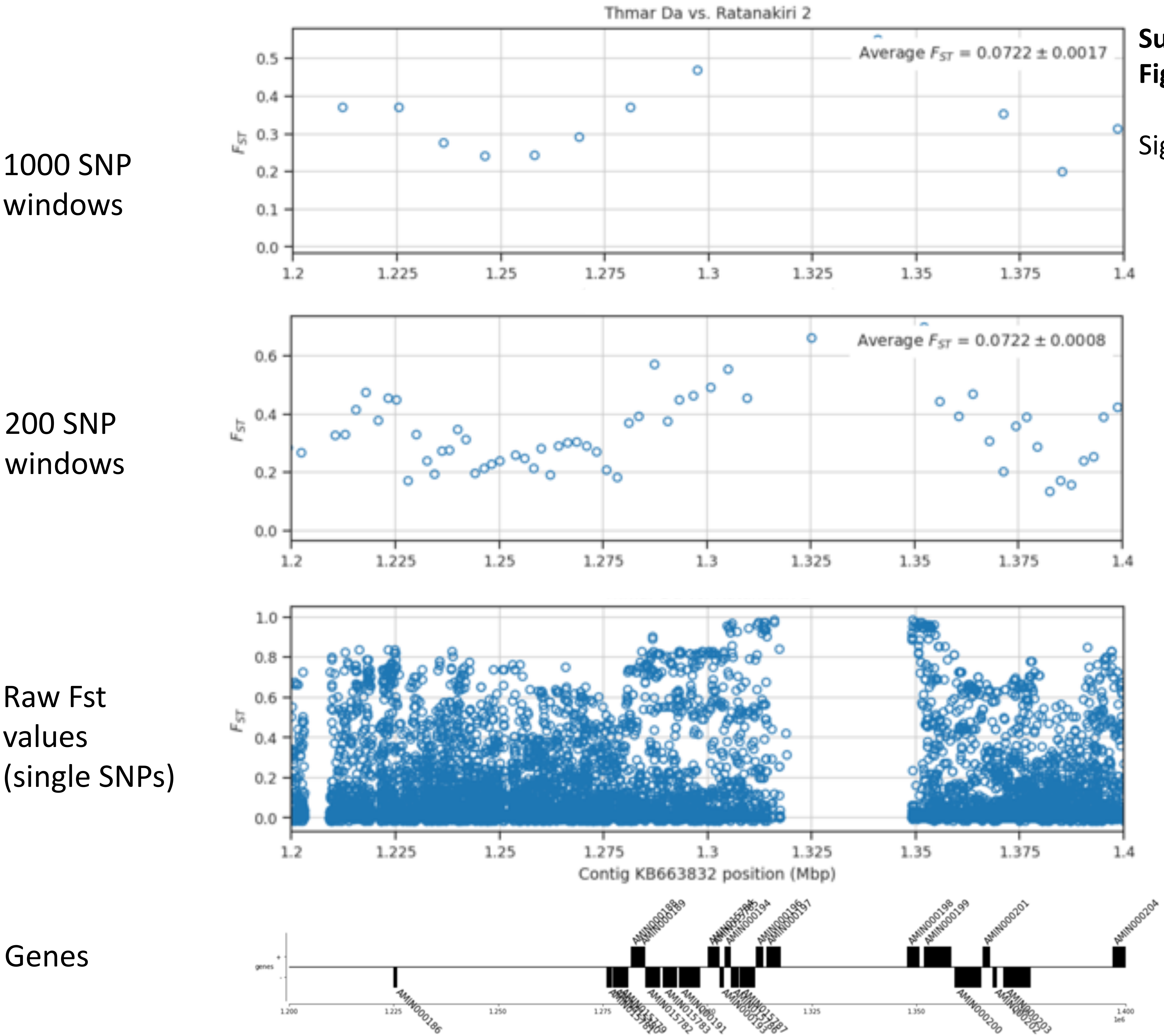

Supplementary Figure 6

Signal R

1000 SNP windows

200 SNP windows

Raw Fst values (single SNPs)

Genes

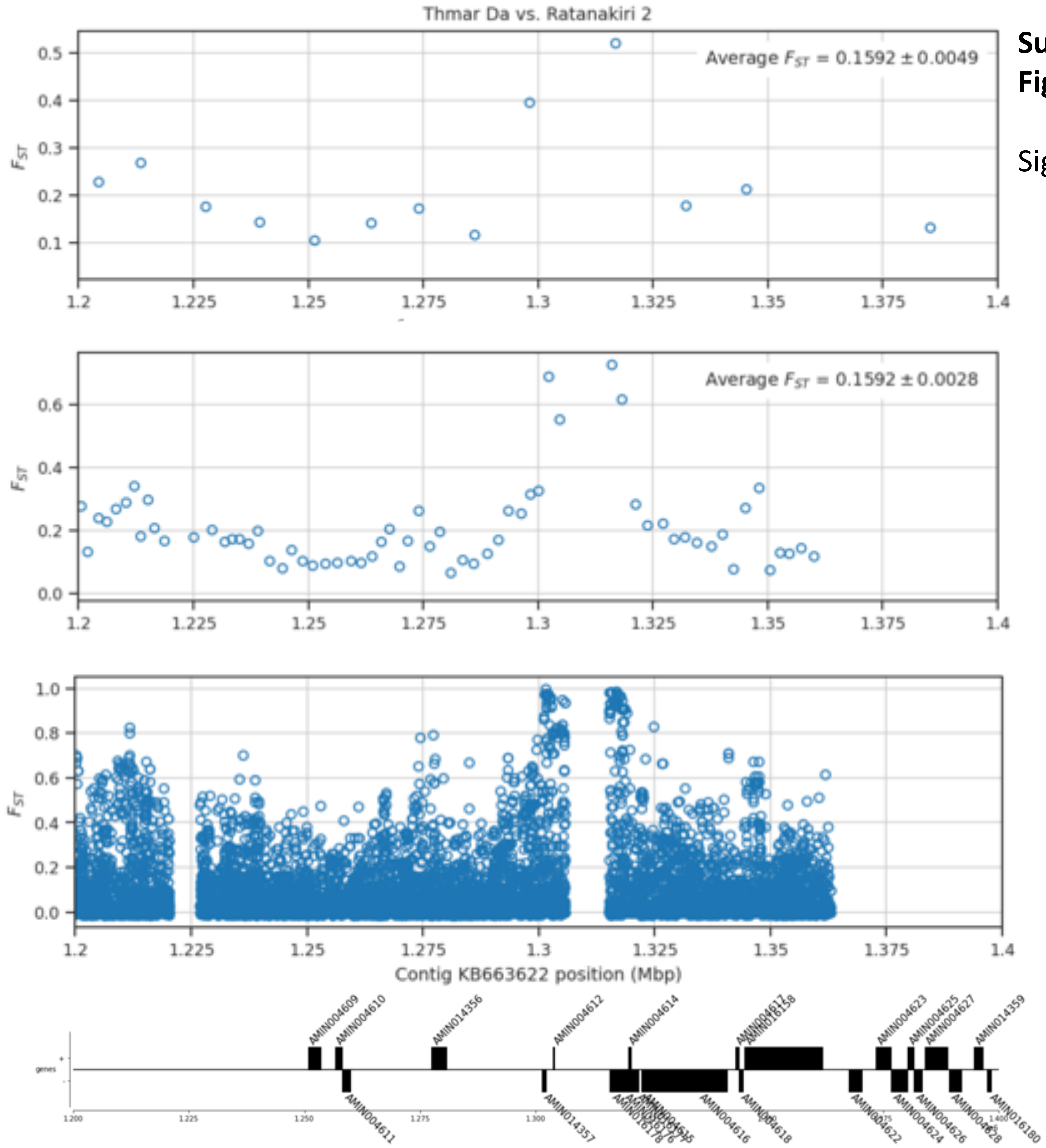

Supplementary Figure 6

Signal S

1000 SNP windows

200 SNP windows

Raw Fst values (single SNPs)

Genes

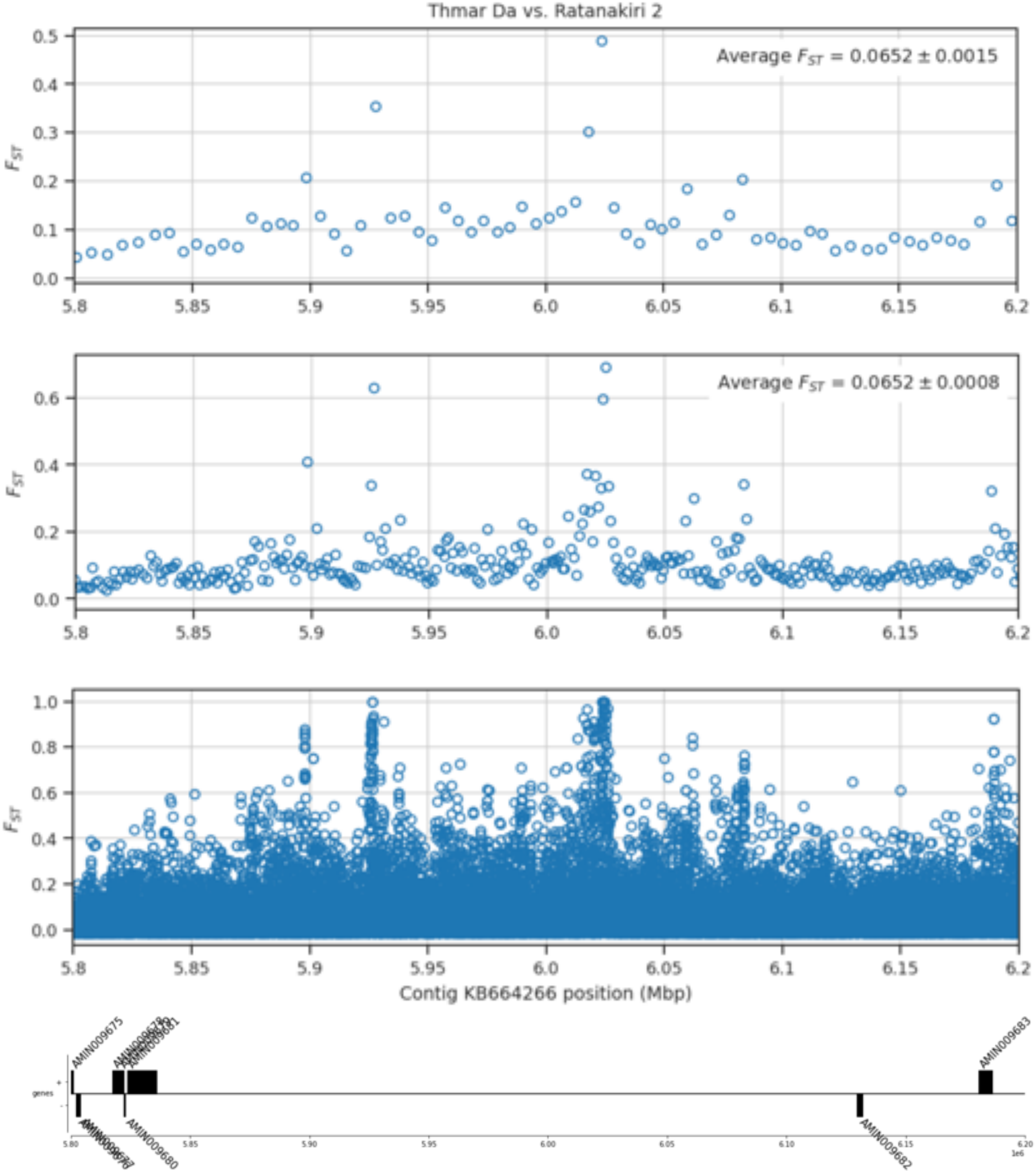

Supplementary Figure 6

Signal T

1000 SNP windows

200 SNP windows

Raw Fst values (single SNPs)

Genes

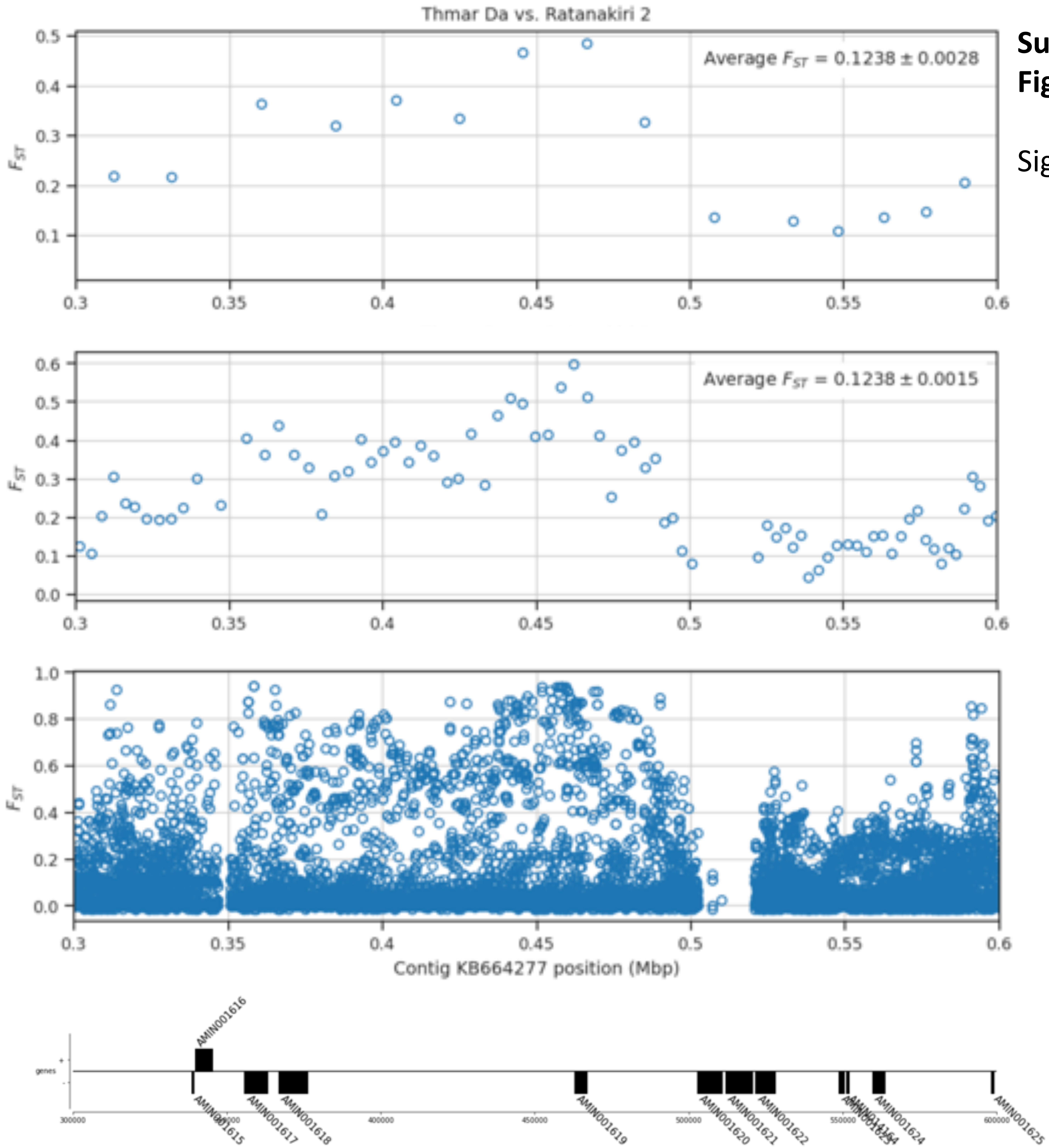

Supplementary Figure 6

Signal U

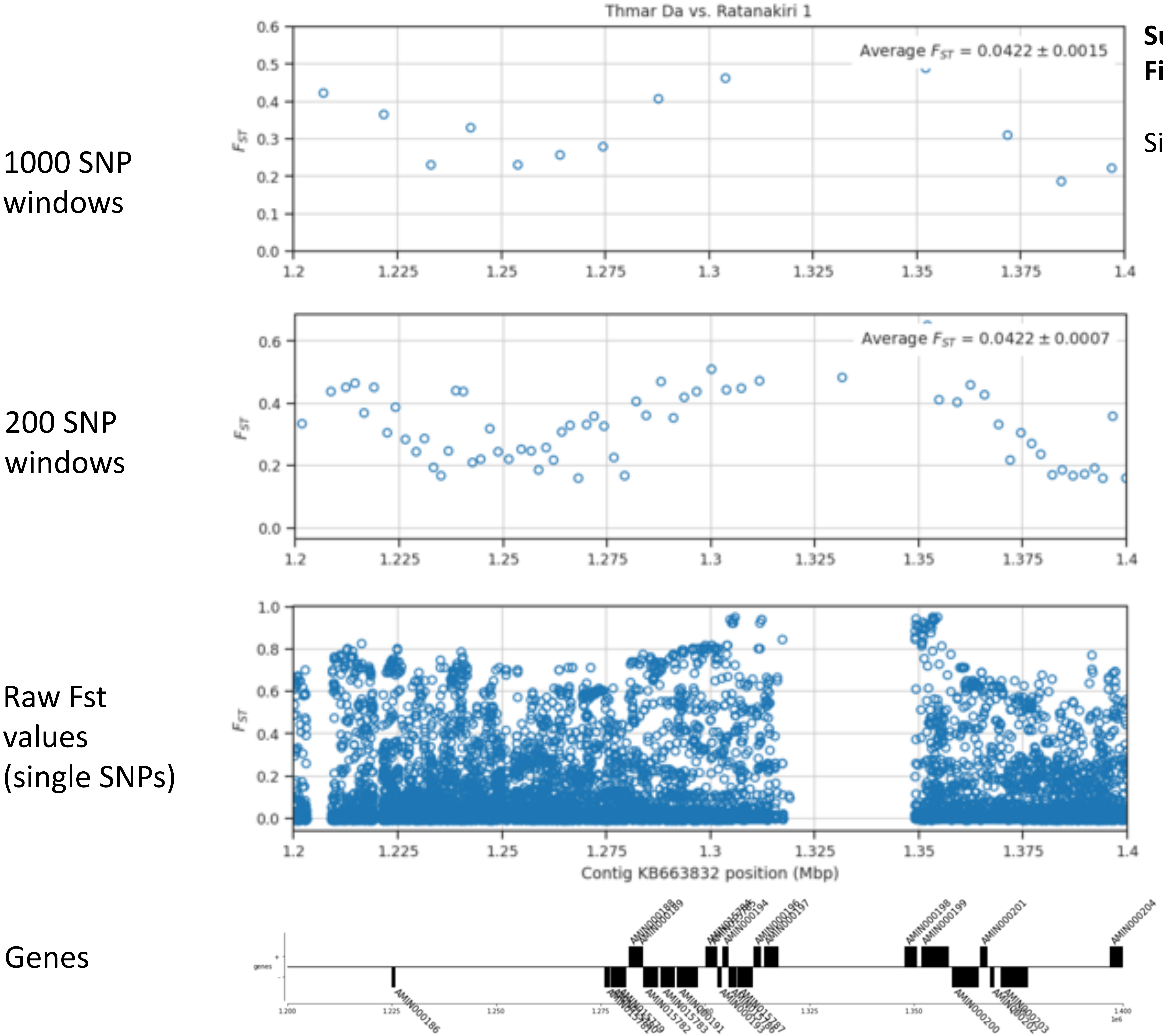

Supplementary Figure 6

Signal V

1000 SNP windows

200 SNP windows

Raw Fst values (single SNPs)

Genes

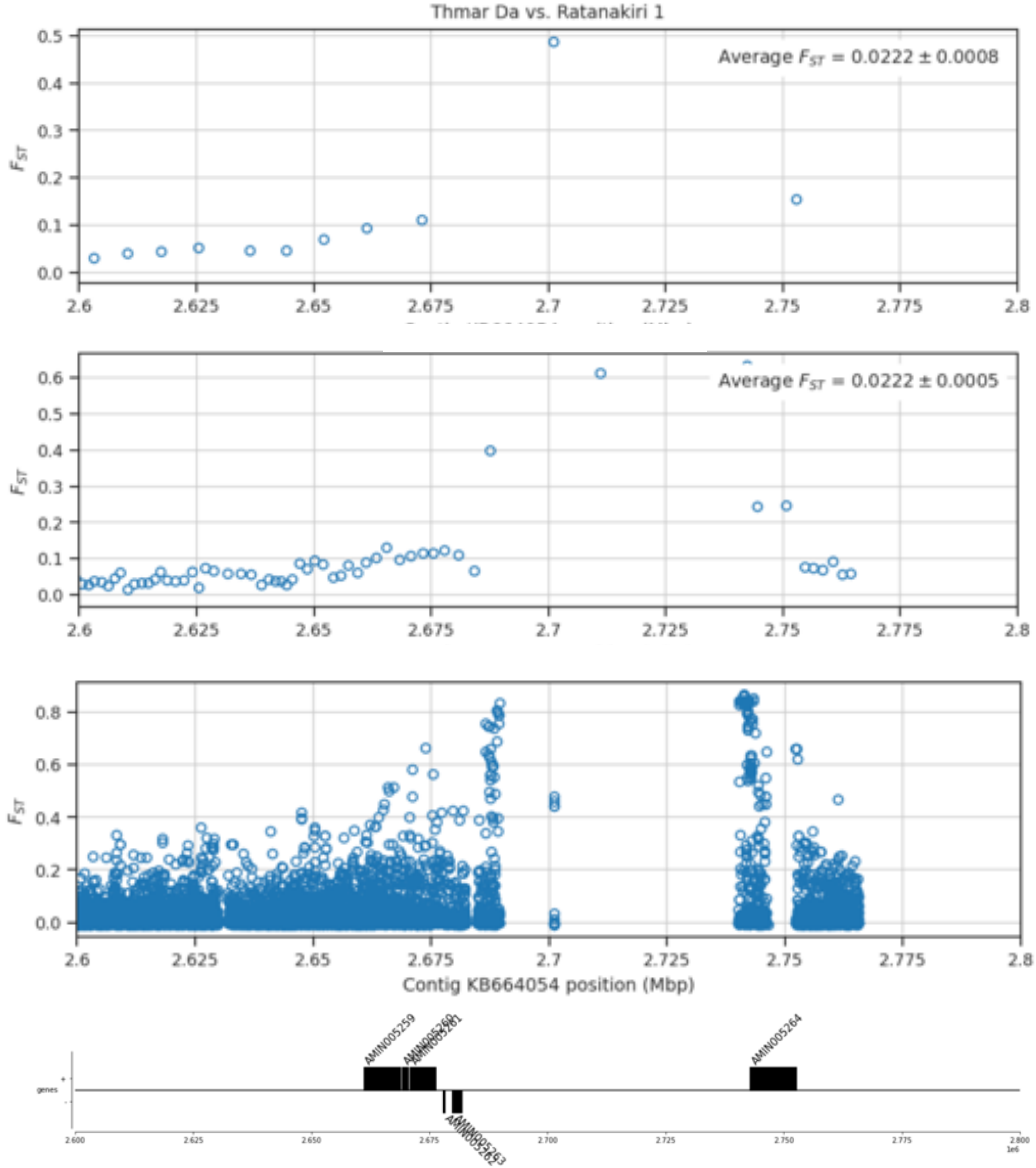

Supplementary Figure 6

Signal W

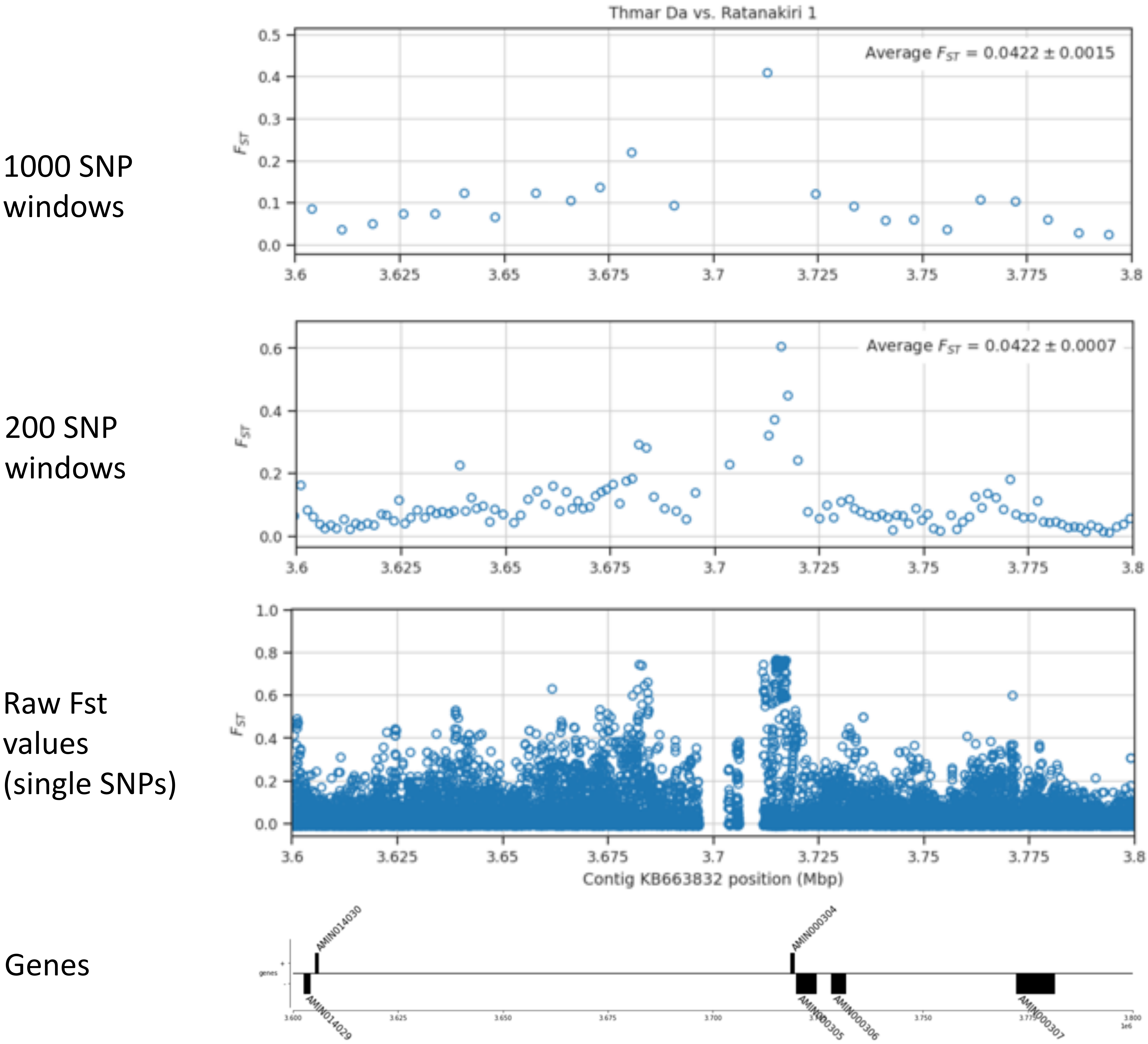

Supplementary Figure 6

Signal X

1000 SNP windows

200 SNP windows

Raw Fst values (single SNPs)

Genes

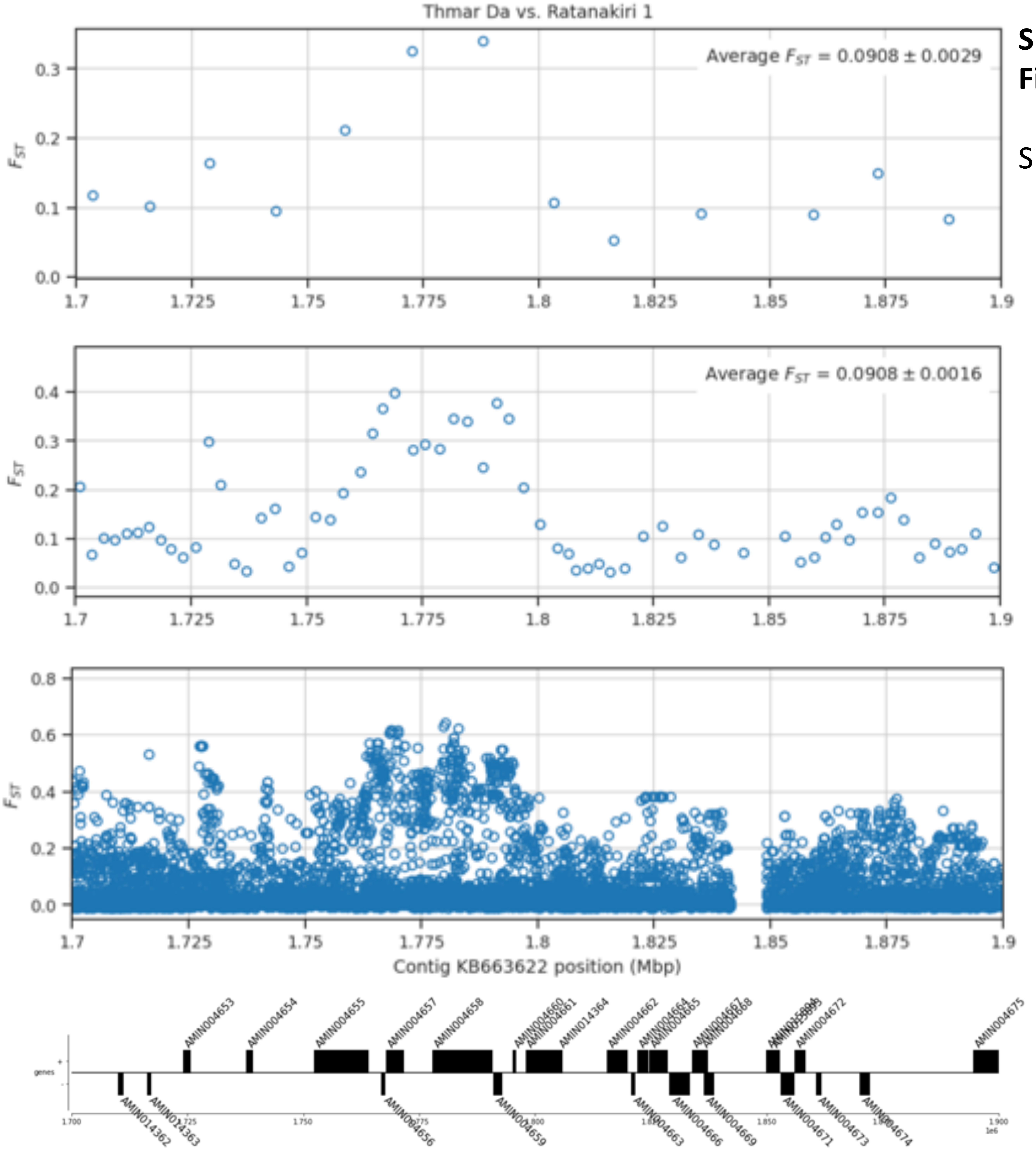

Supplementary Figure 6

Signal Y

1000 SNP windows

200 SNP windows

Raw Fst values (single SNPs)

Genes

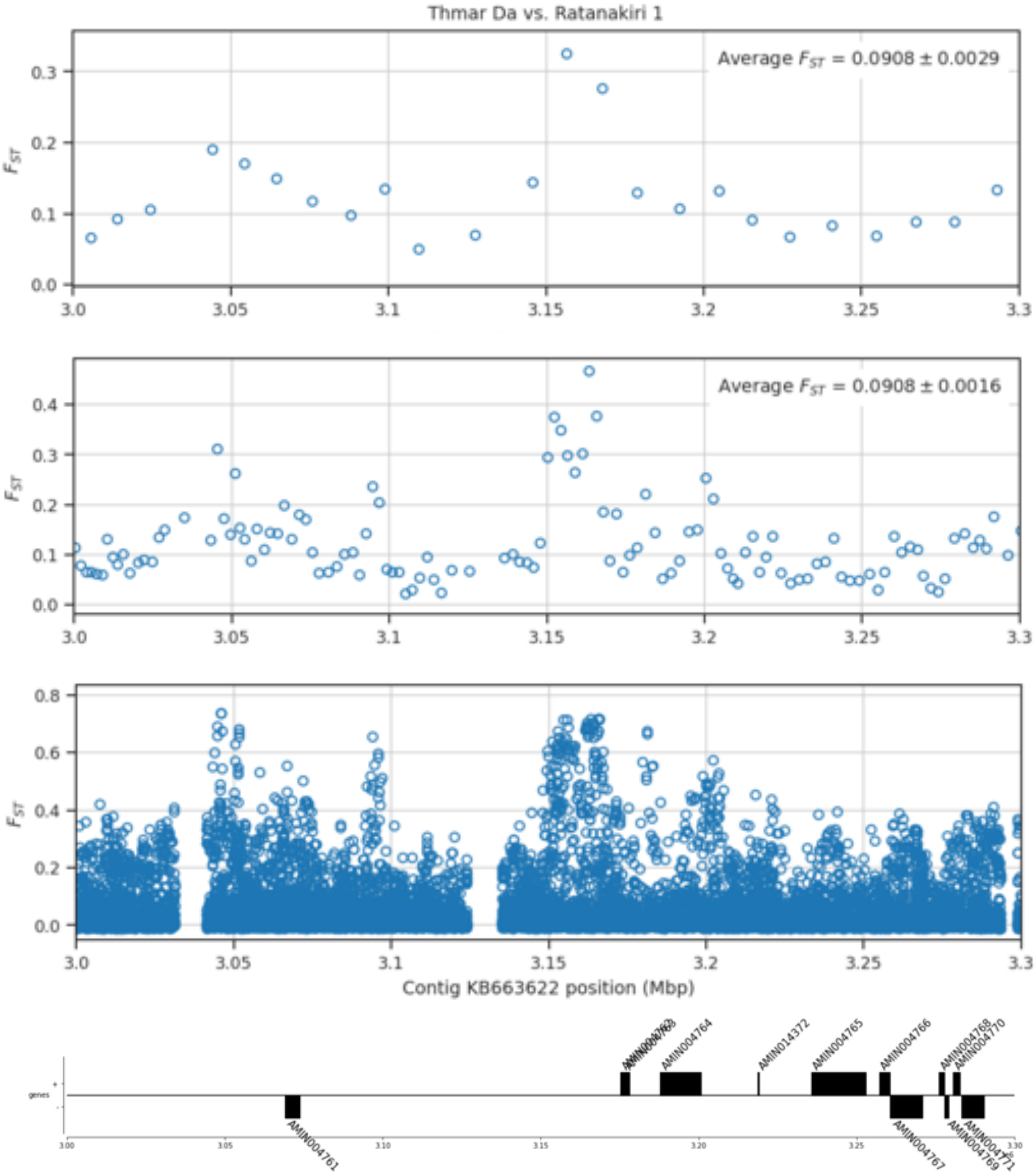

Supplementary Figure 6

Signal Z

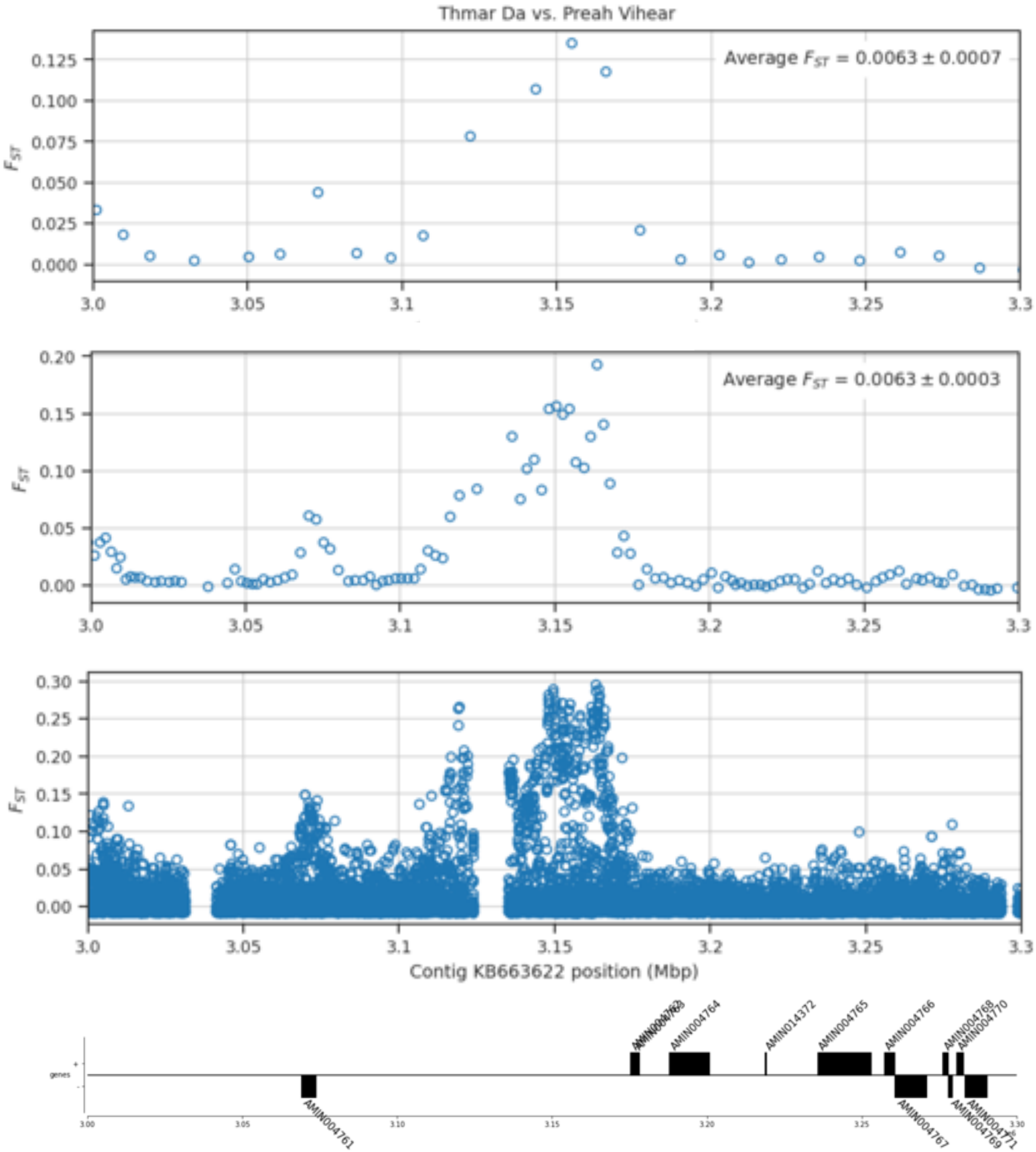

1000 SNP windows

200 SNP windows

Raw Fst values (single SNPs)

Genes

Supplementary Figure 6

Signal AA

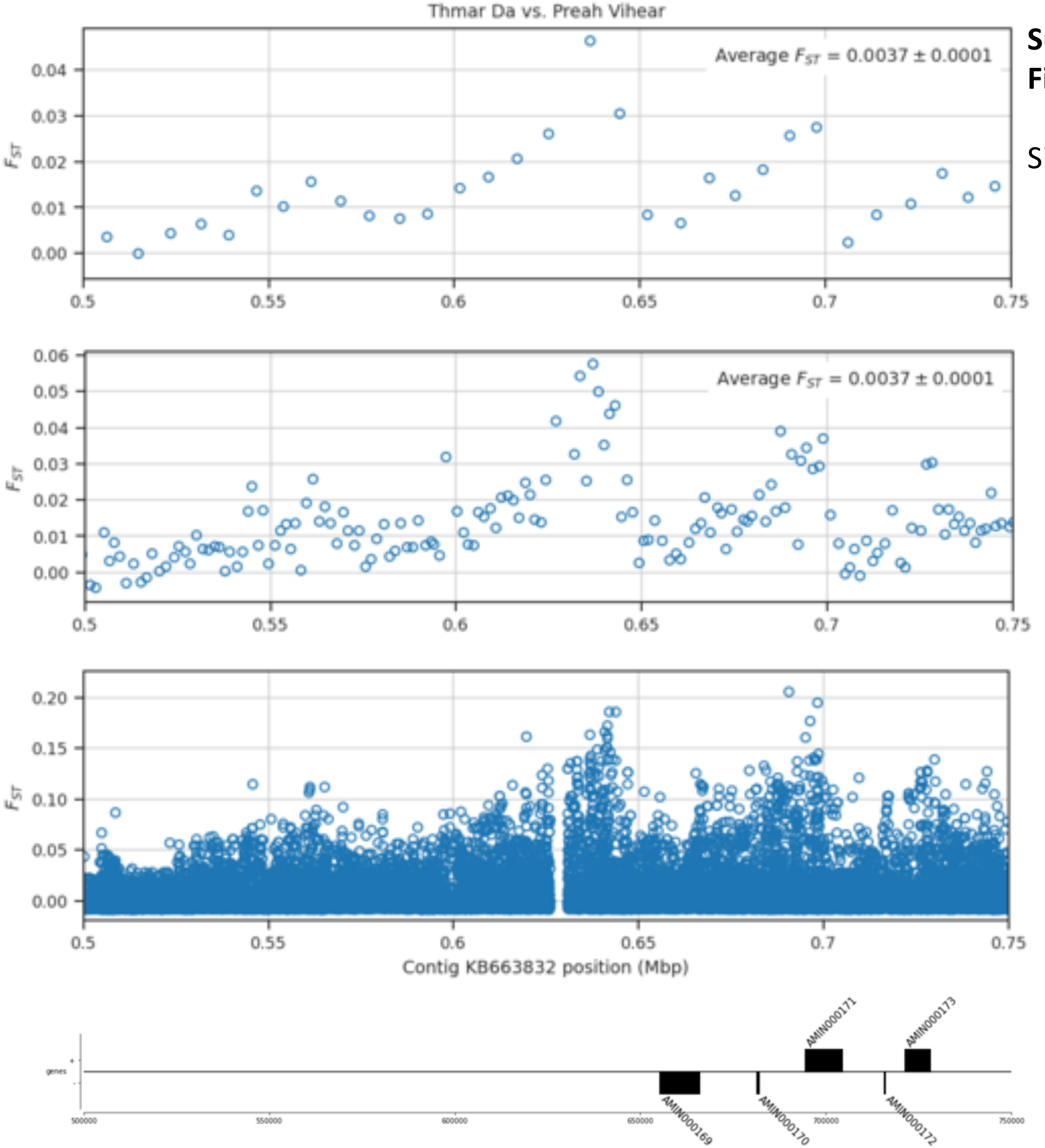

1000 SNP windows

200 SNP windows

Raw Fst values (single SNPs)

Genes

Supplementary Figure 6

Signal BB

1000 SNP windows

200 SNP windows

Raw Fst values (single SNPs)

Genes

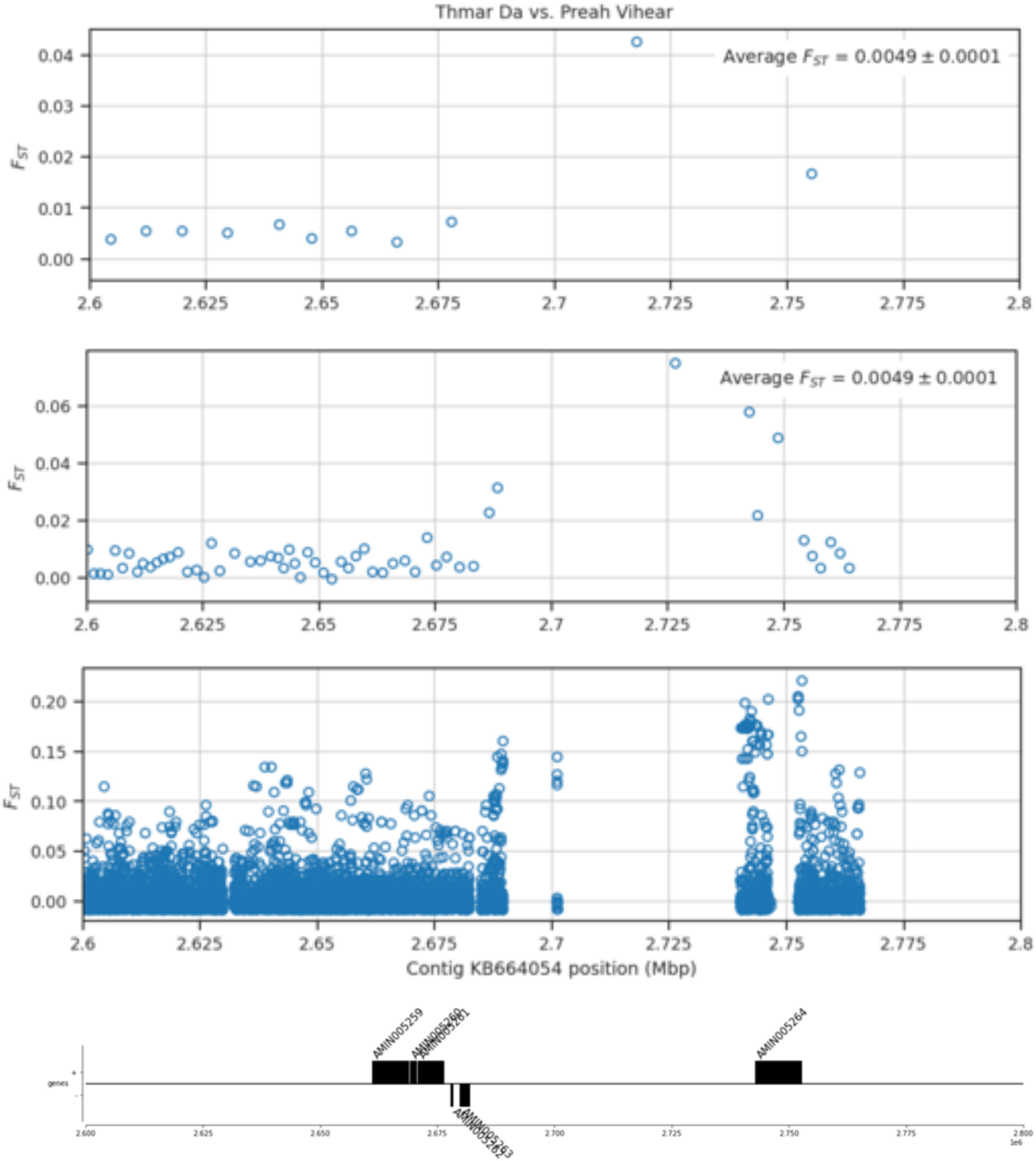

Supplementary Figure 6

Signal CC

1000 SNP windows

200 SNP windows

Raw Fst values (single SNPs)

Genes

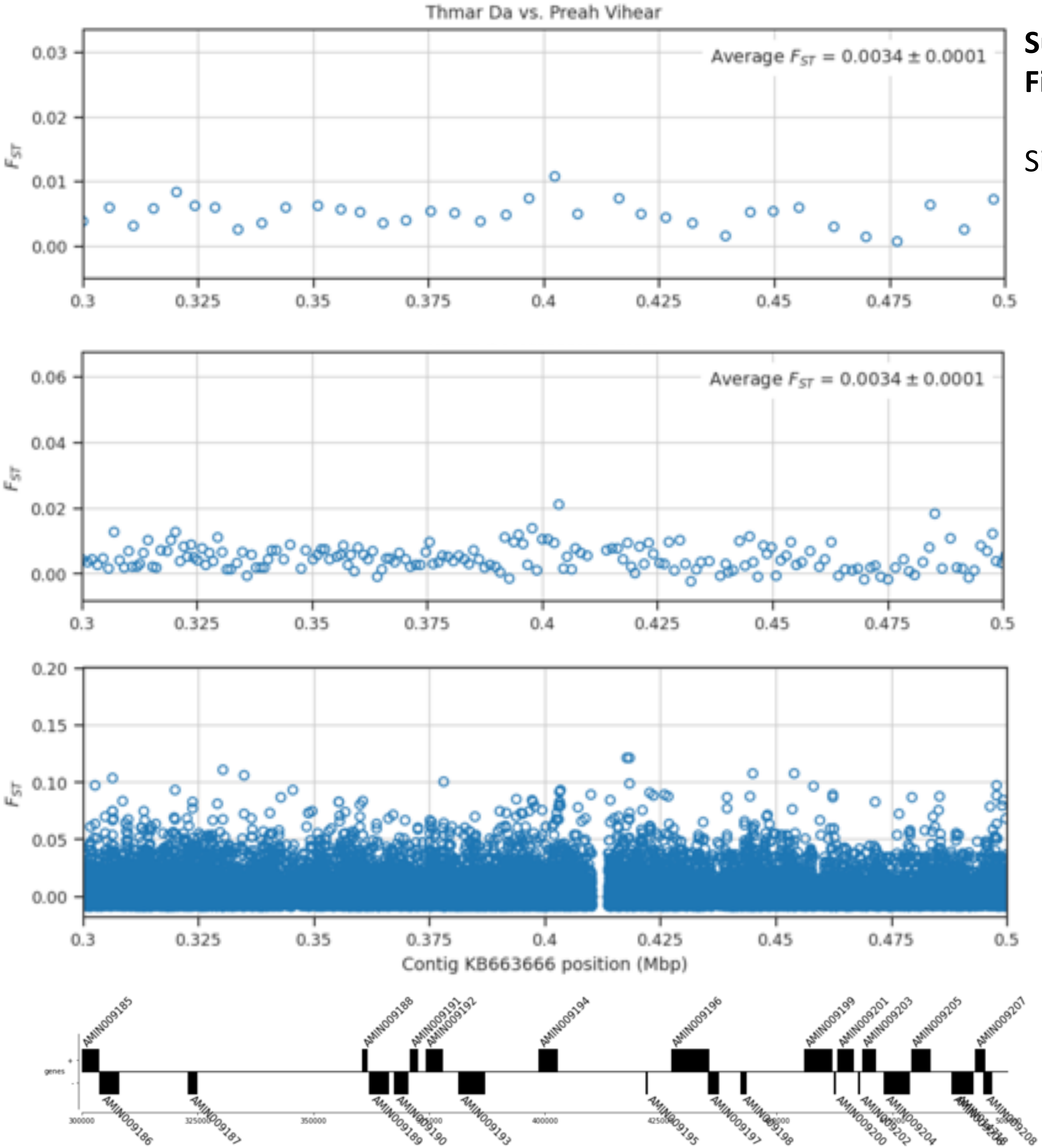

Supplementary Figure 6

Signal DD

1000 SNP windows

200 SNP windows

Raw Fst values (single SNPs)

Genes

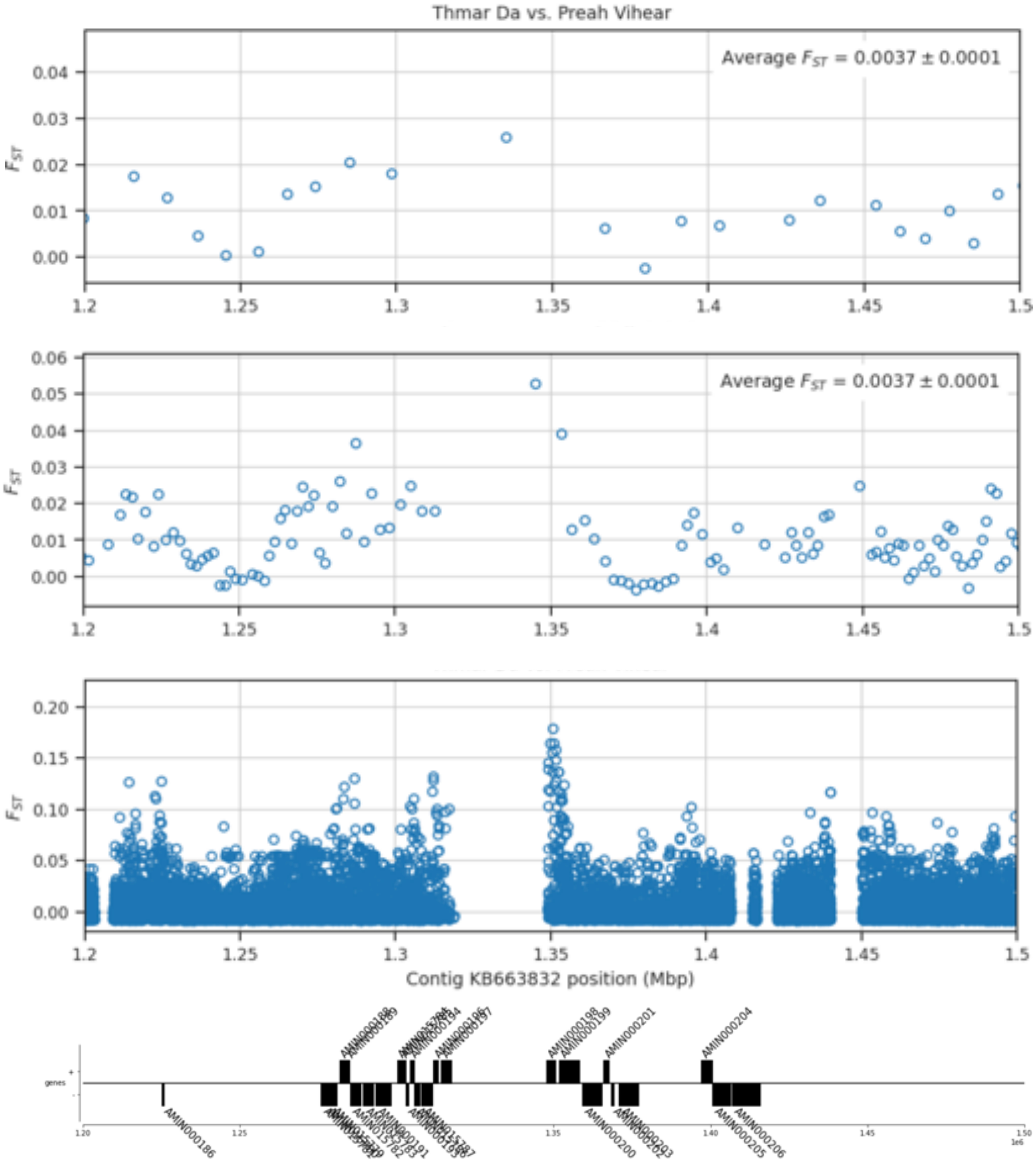

Supplementary Figure 7 – Histograms of mean coverage by sample and population

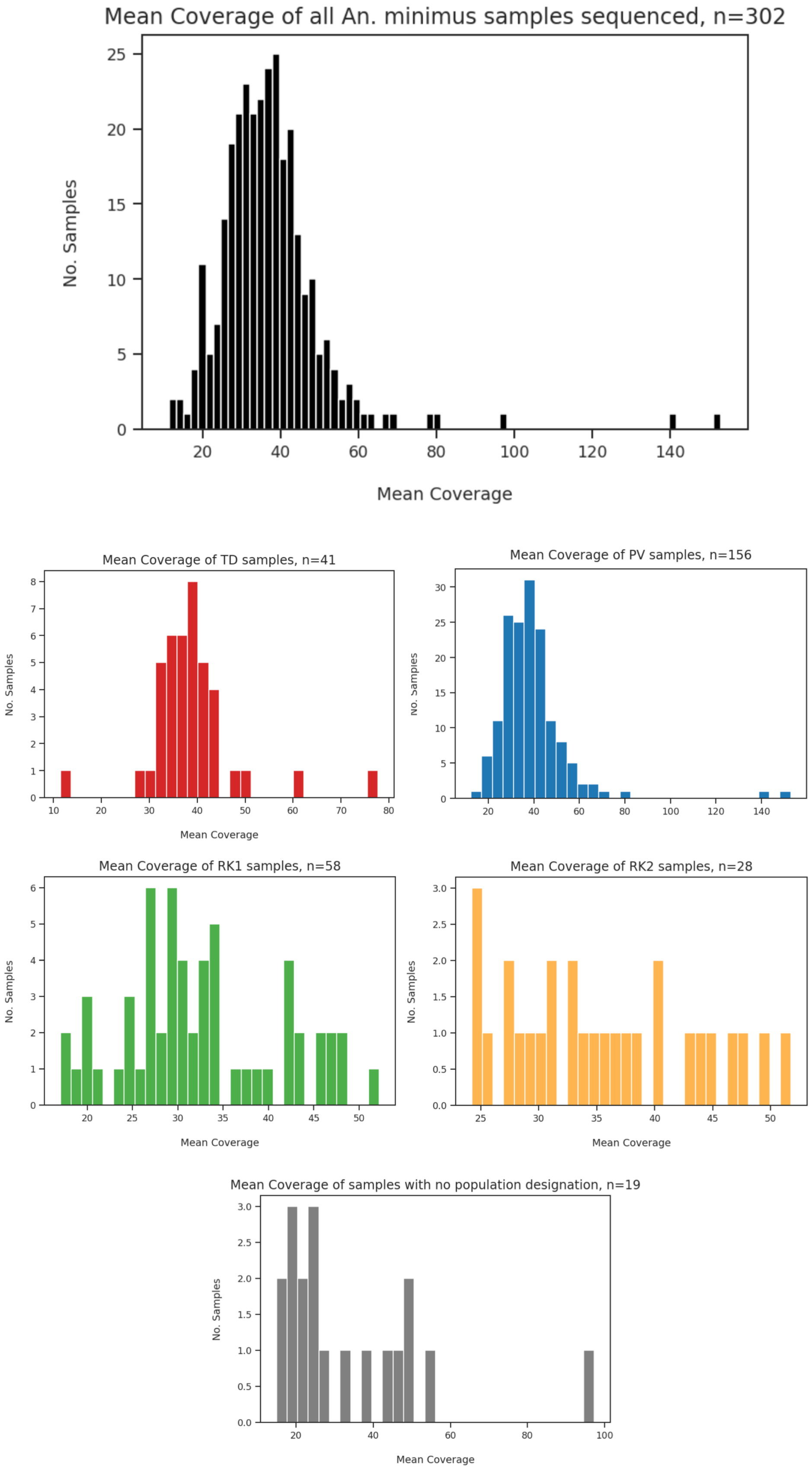

Supplementary Figure 8.1

RK1 vs. RK2

Raw Fst plots over the 10 largest AminM1 contigs

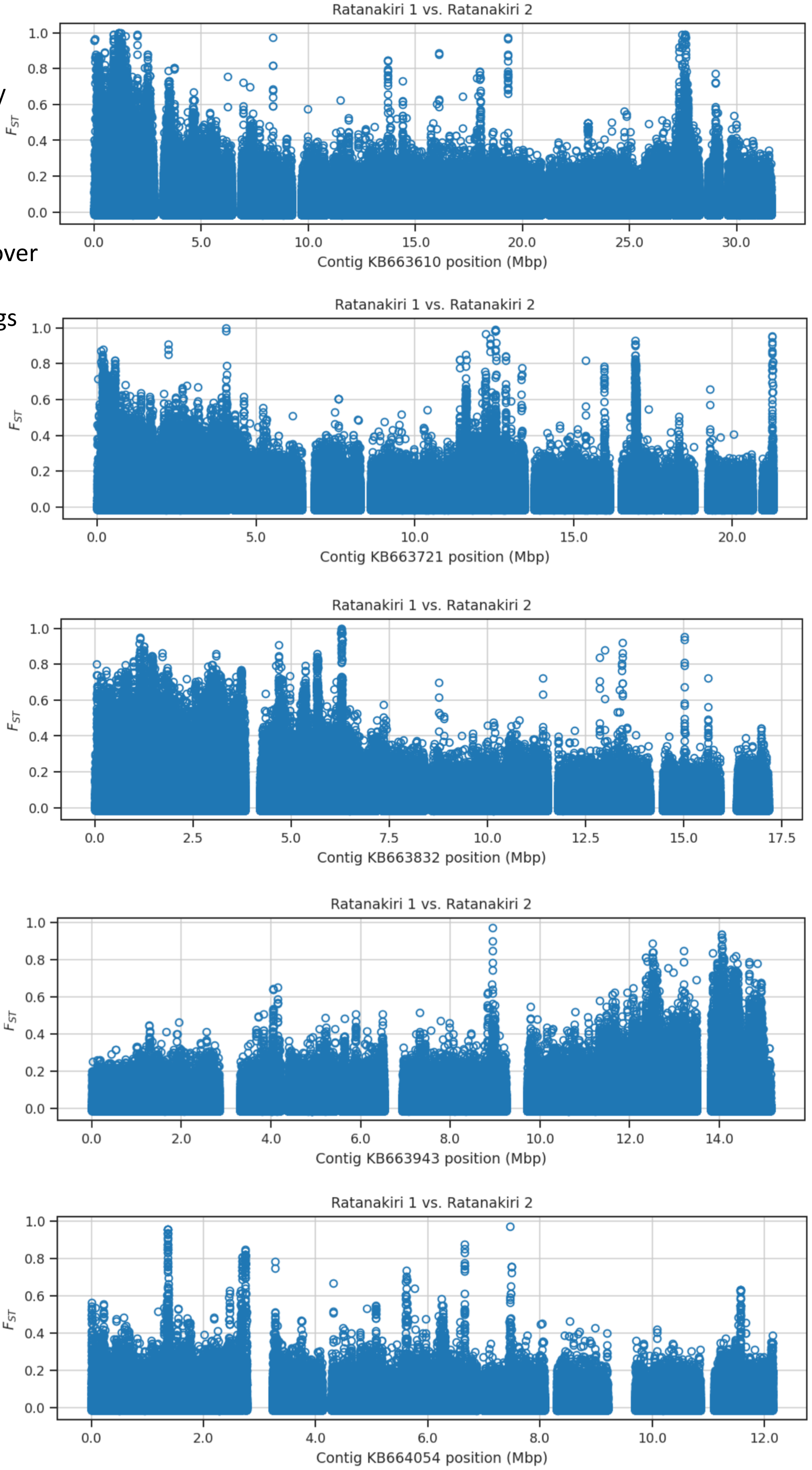

Supplementary Figure 8.1

RK1 vs. RK2

Raw Fst plots over the 10 largest AminM1 contigs

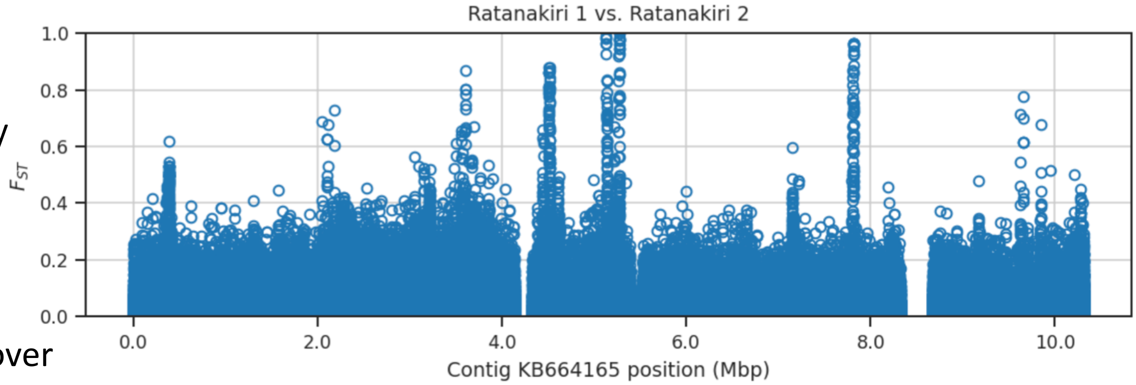

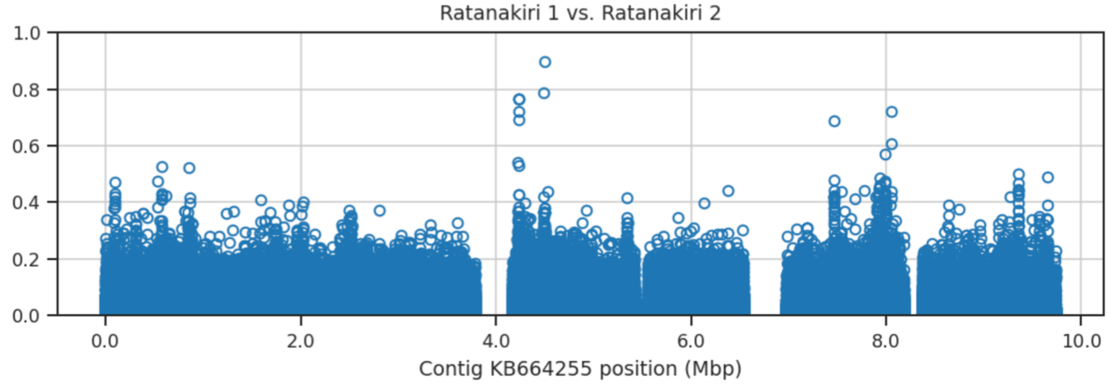

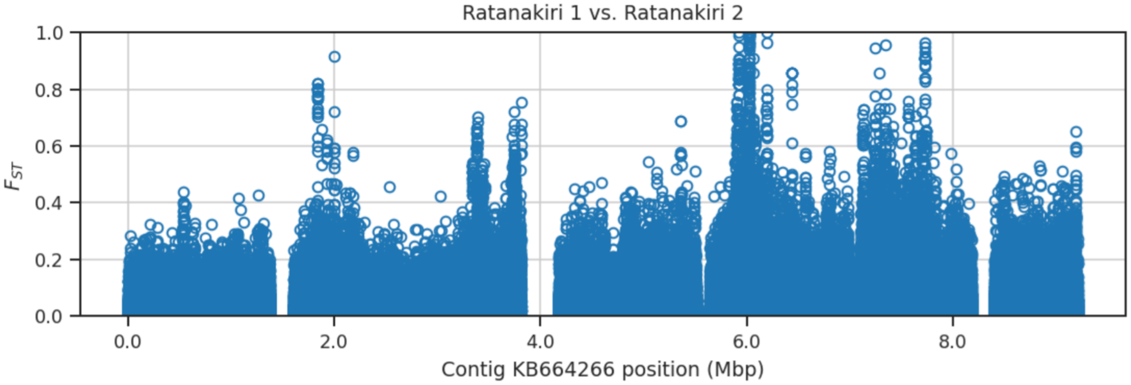

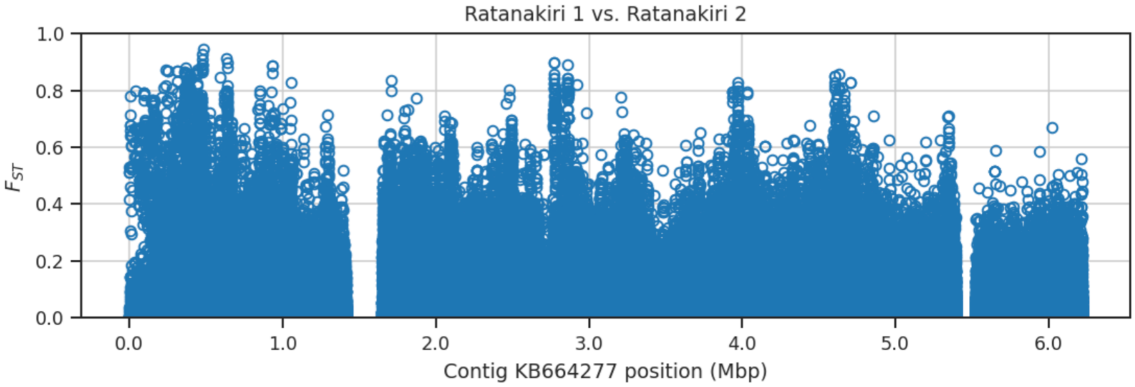

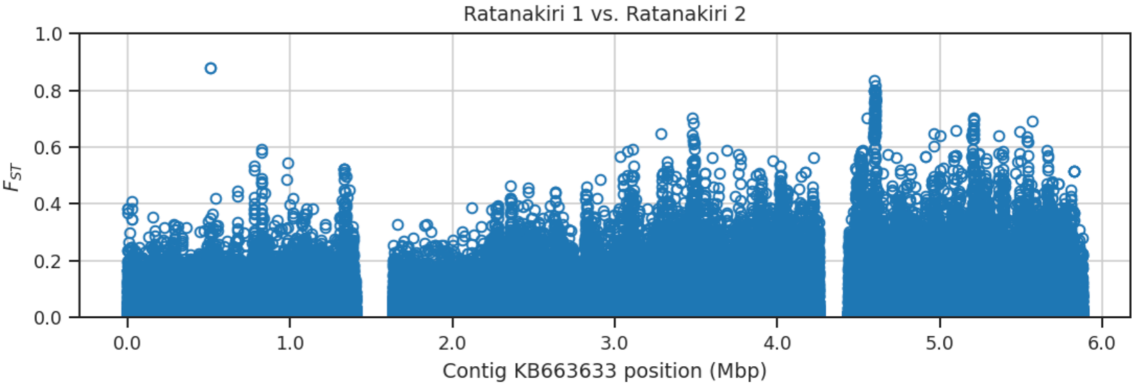

Supplementary Figure 8.2

PV vs. RK2

Raw Fst plots over the 10 largest AminM1 contigs

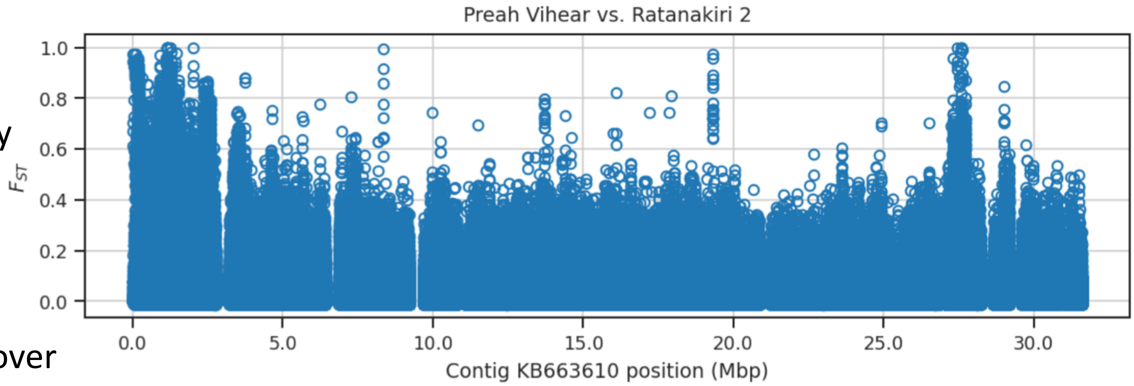

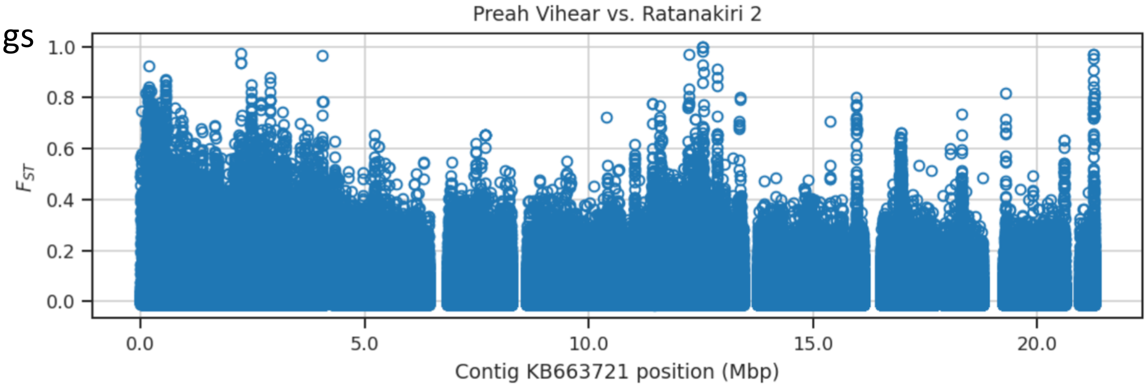

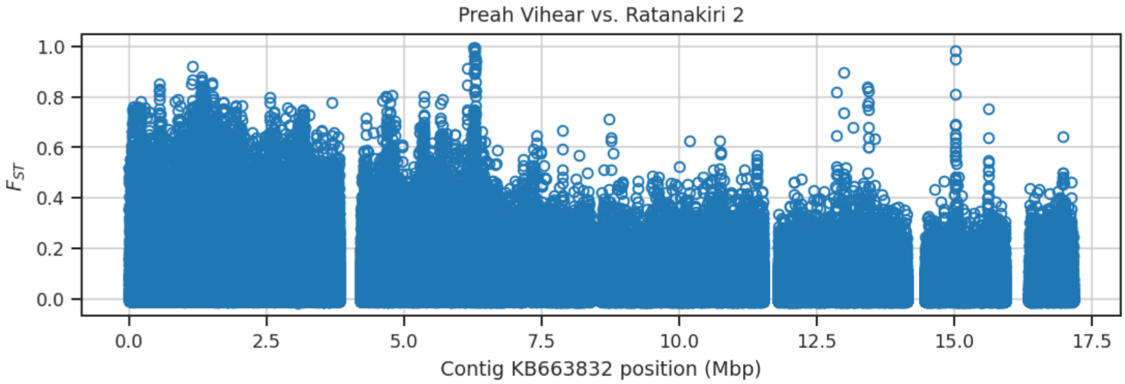

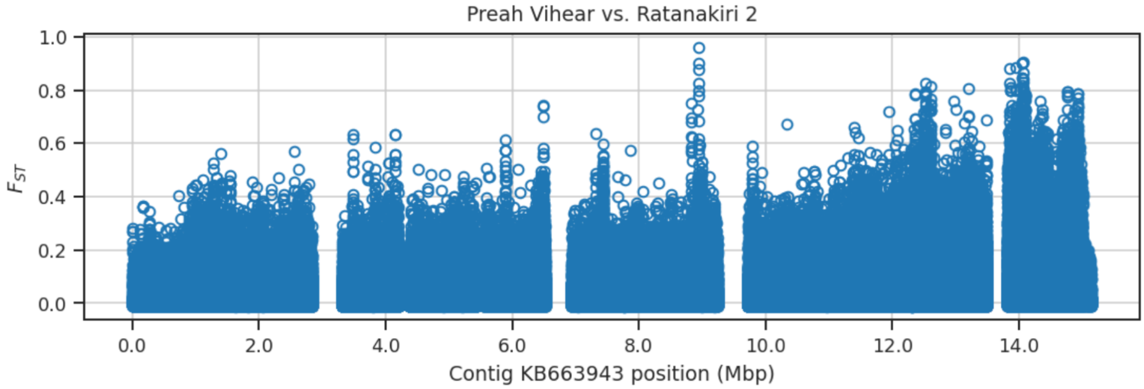

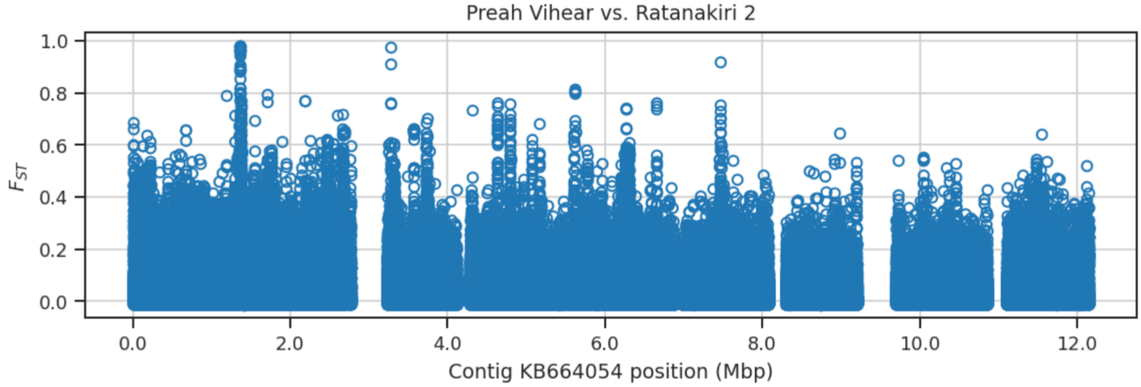

Supplementary Figure 8.2

PV vs. RK2

Raw Fst plots over the 10 largest AminM1 contigs

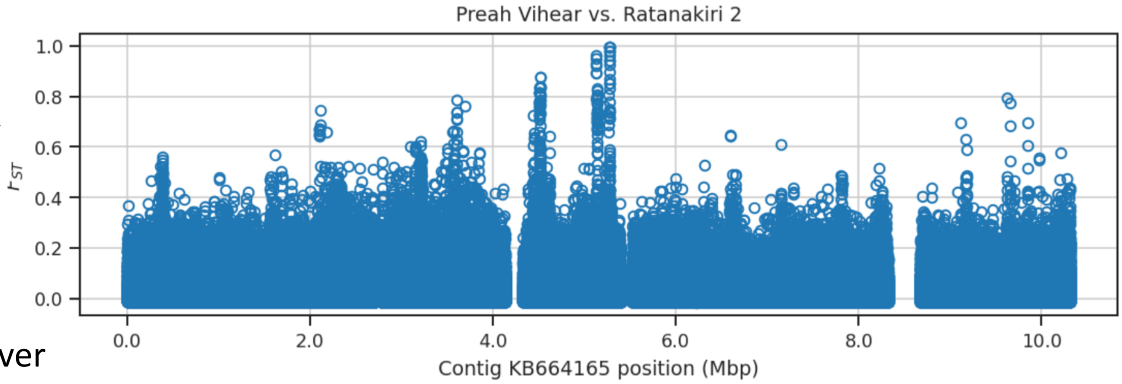

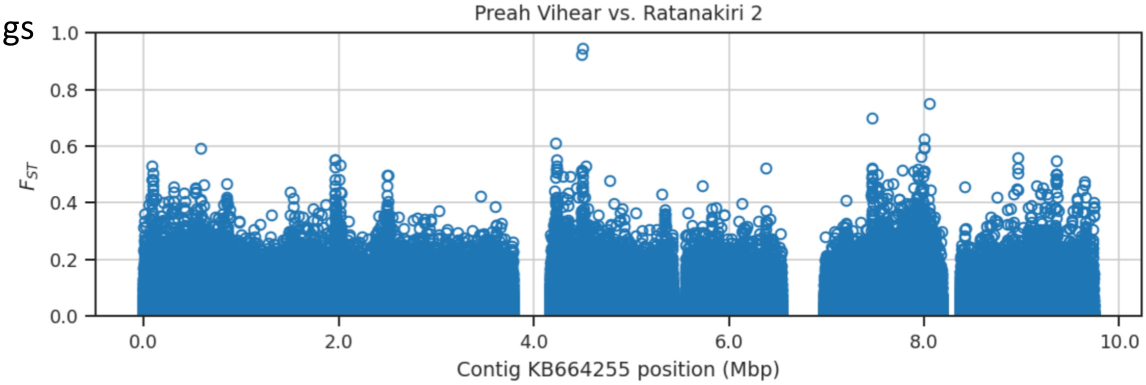

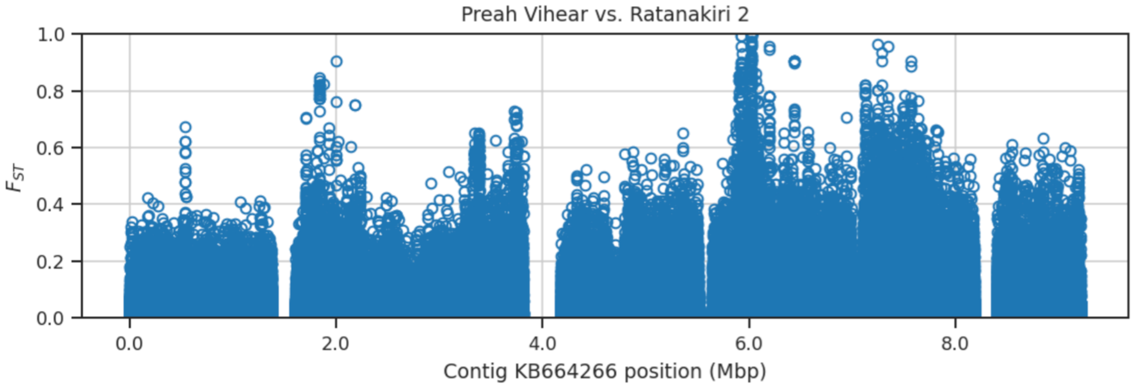

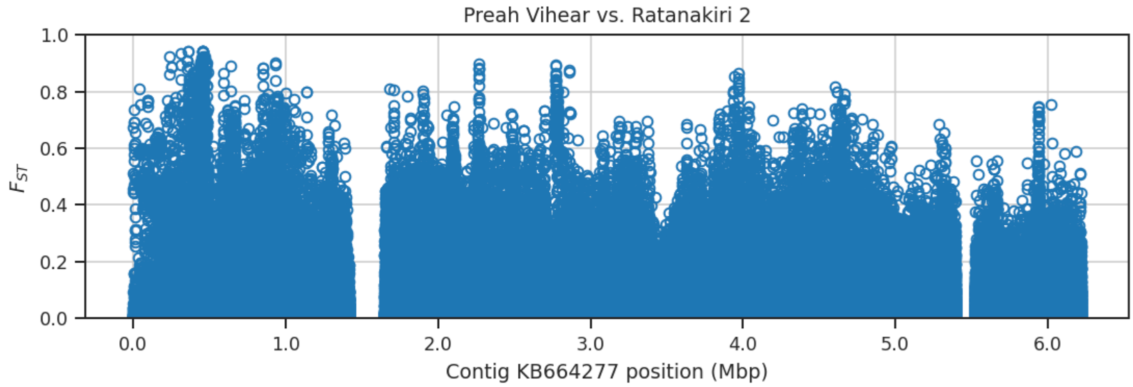

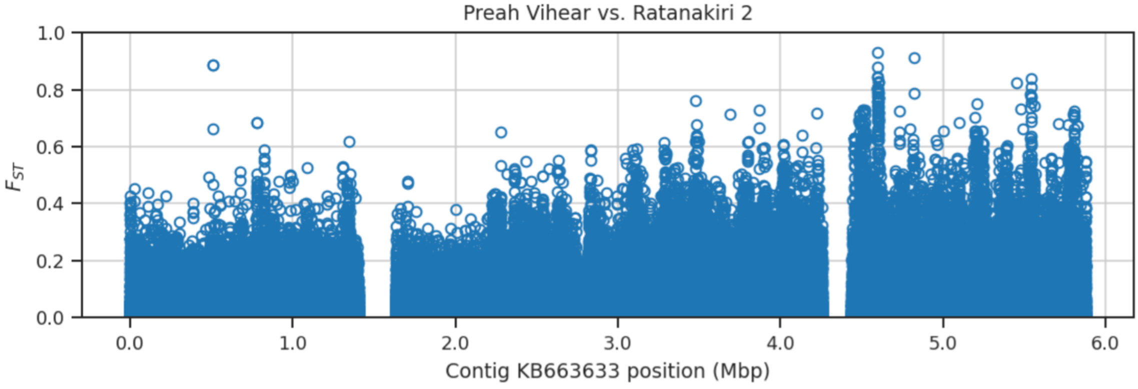

Supplementary Figure 8.3

PV vs. RK1

Raw Fst plots over the 10 largest AminM1 contigs

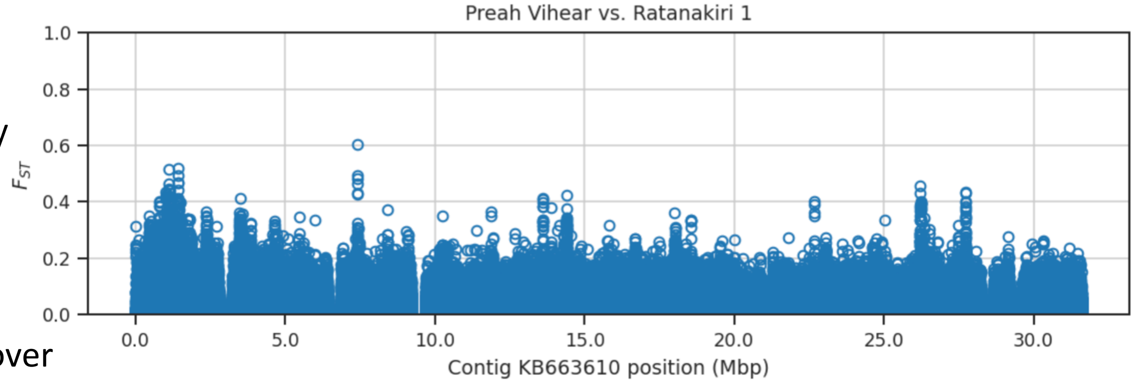

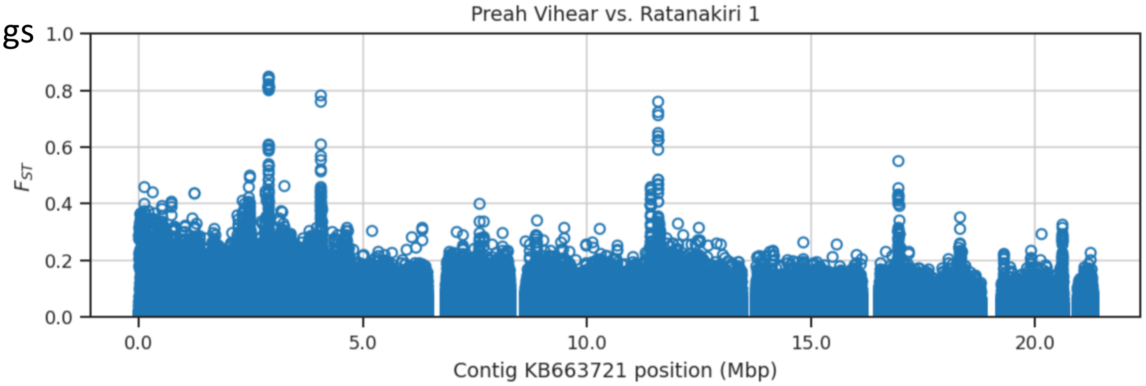

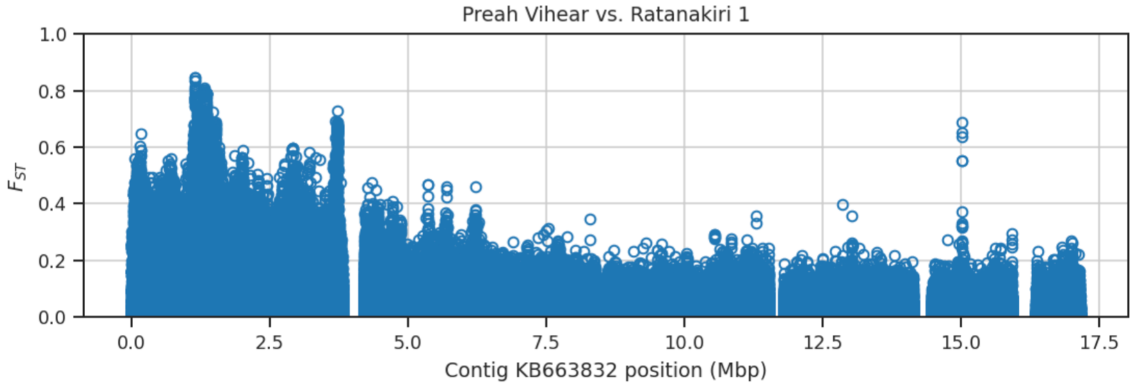

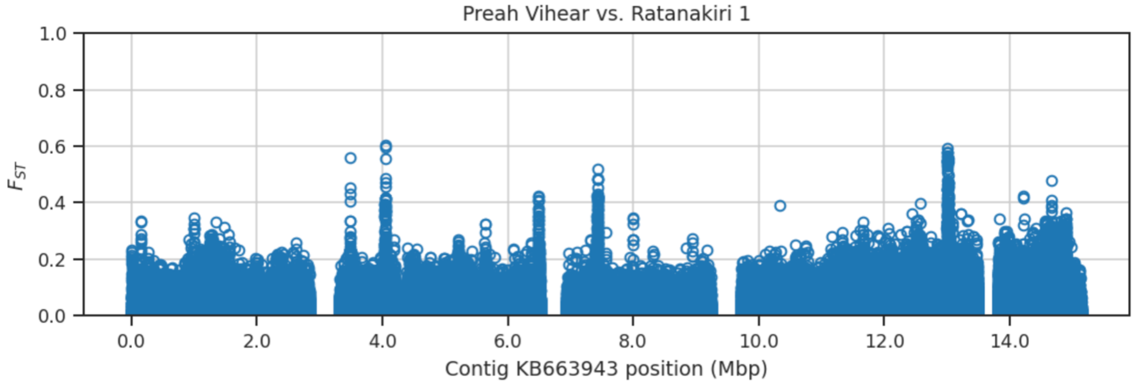

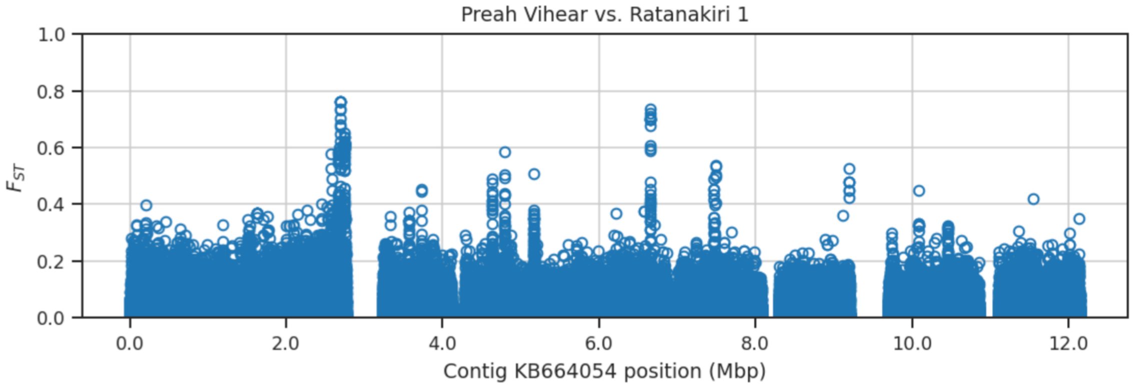

Supplementary Figure 8.3

PV vs. RK1

Raw Fst plots over the 10 largest AminM1 contigs

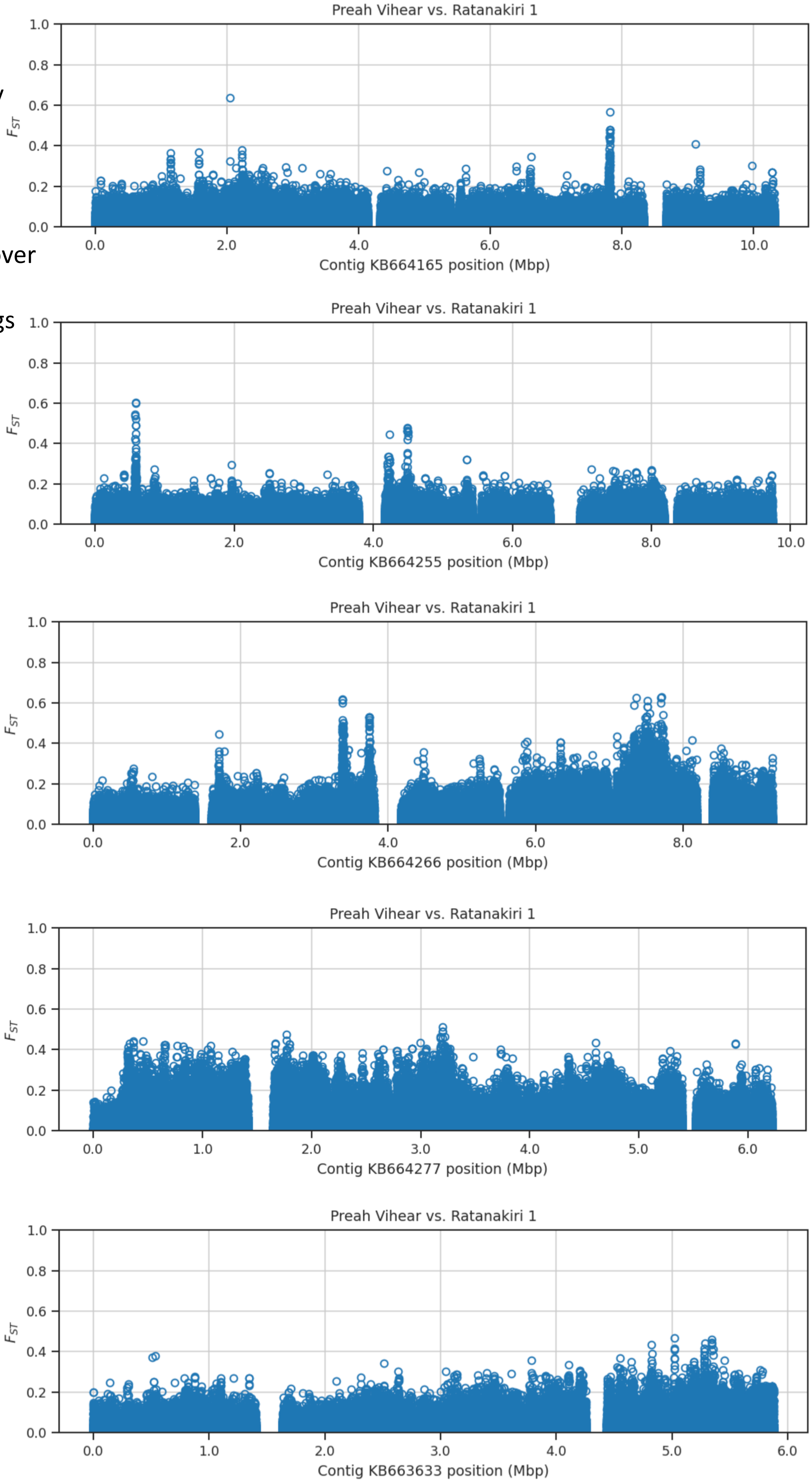

Supplementary Figure 8.4

TD vs. RK2

Raw Fst plots over the 10 largest AminM1 contigs

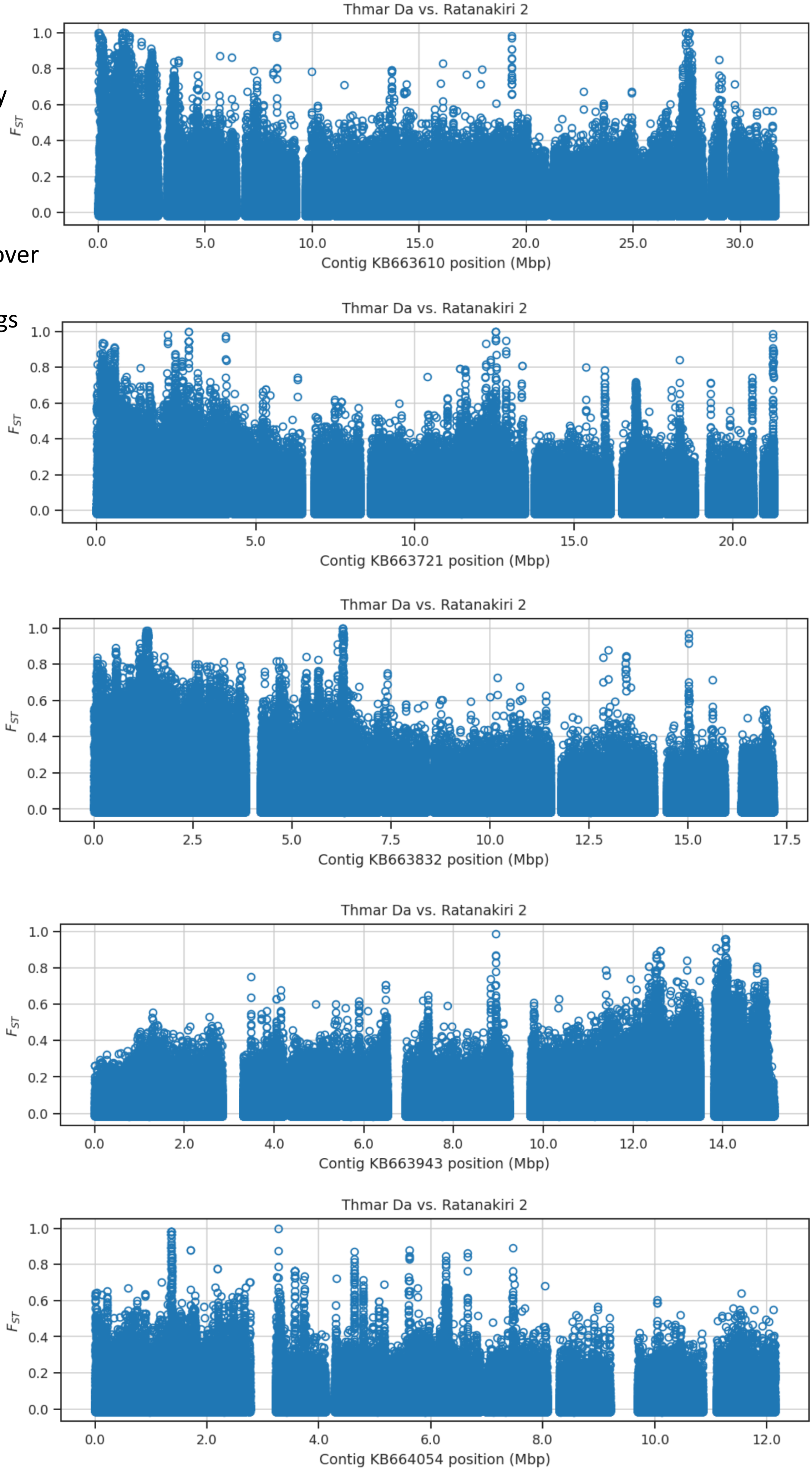

Supplementary Figure 8.4

TD vs. RK2

Raw Fst plots over the 10 largest AminM1 contigs

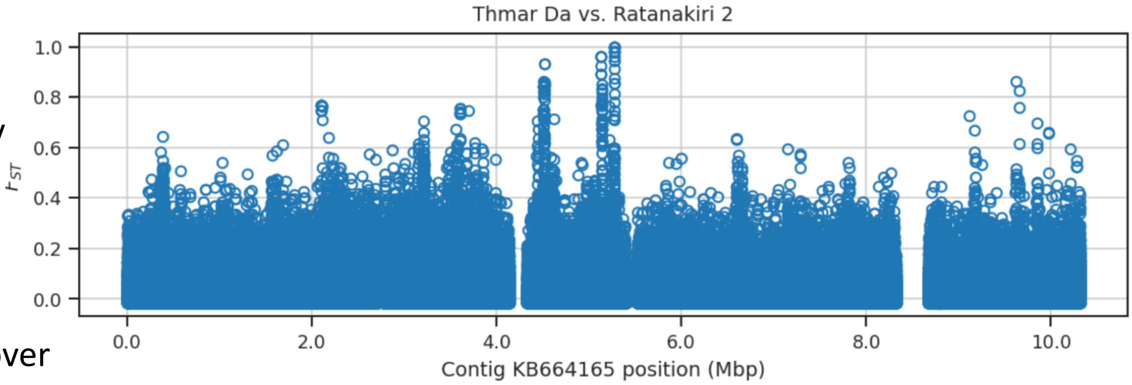

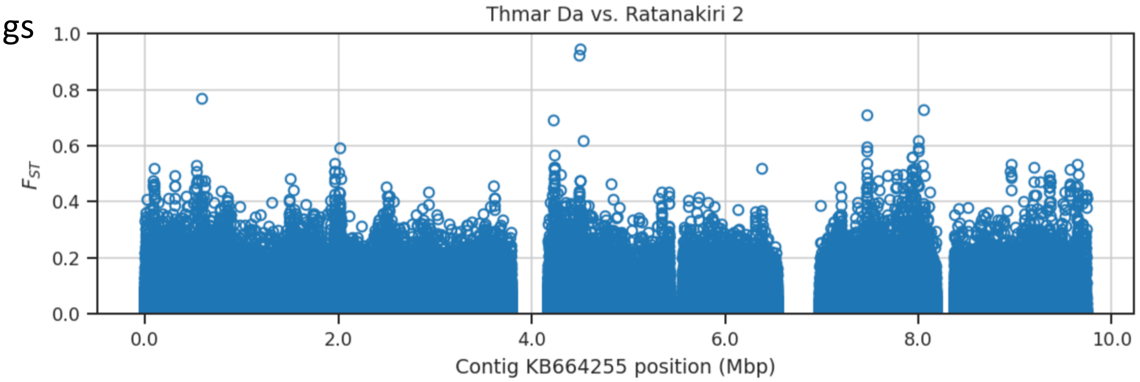

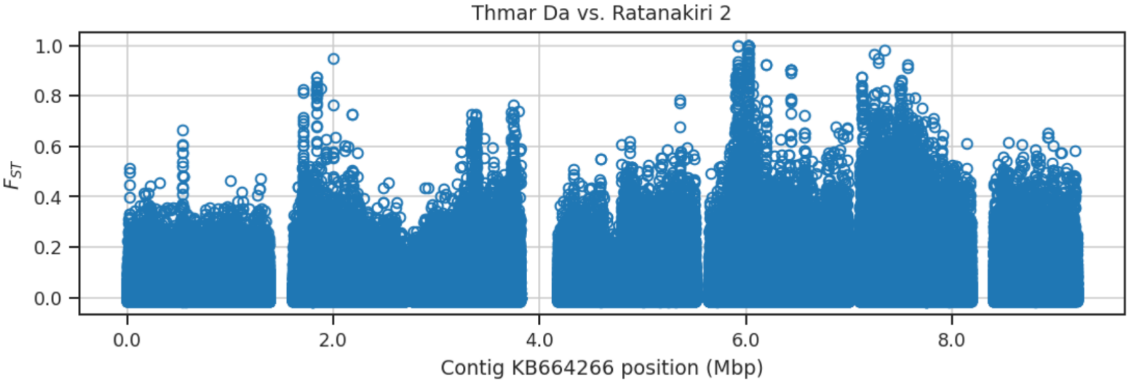

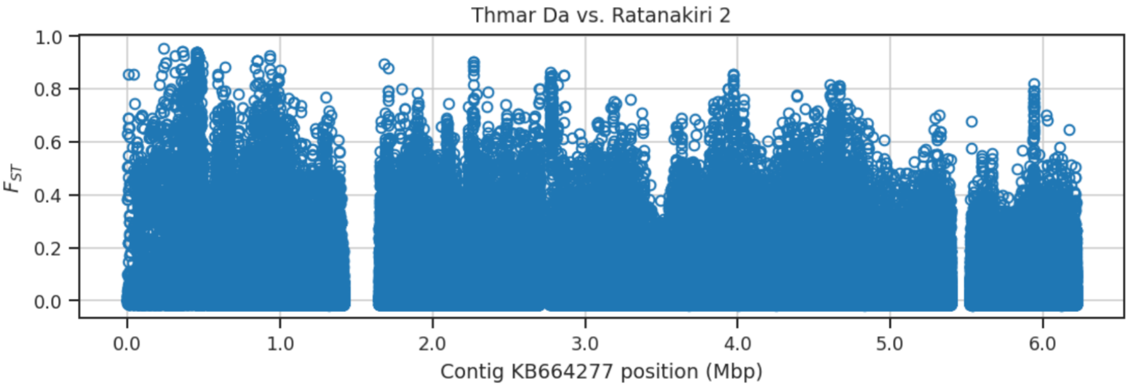

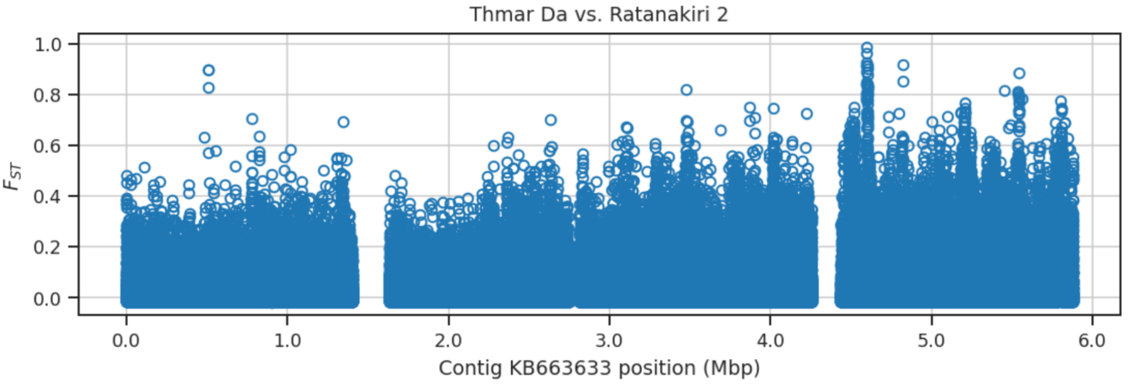

Supplementary Figure 8.5

TD vs. RK1

Raw Fst plots over the 10 largest AminM1 contigs

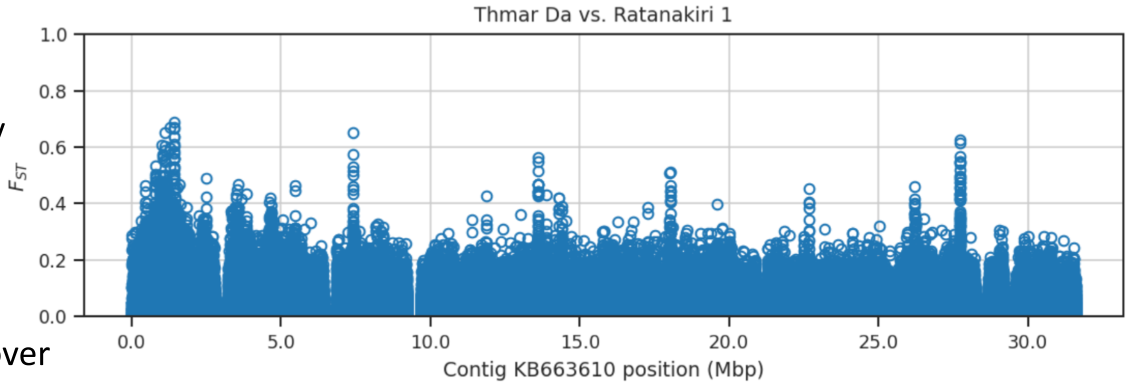

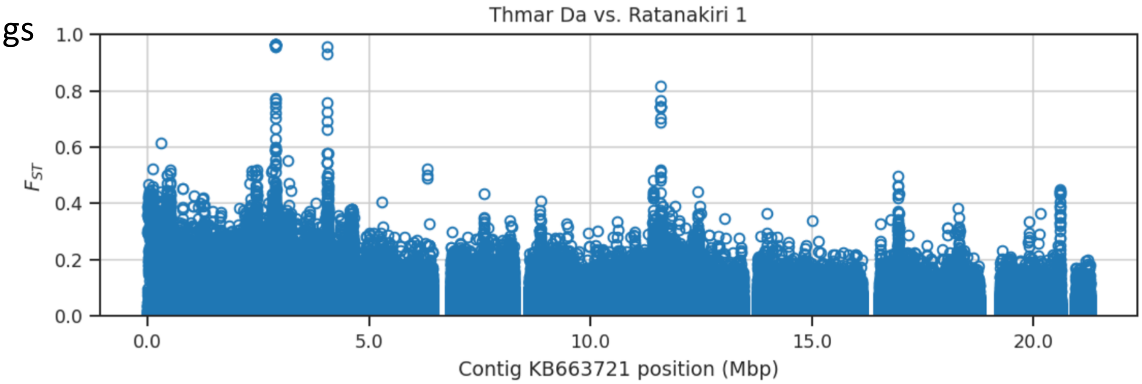

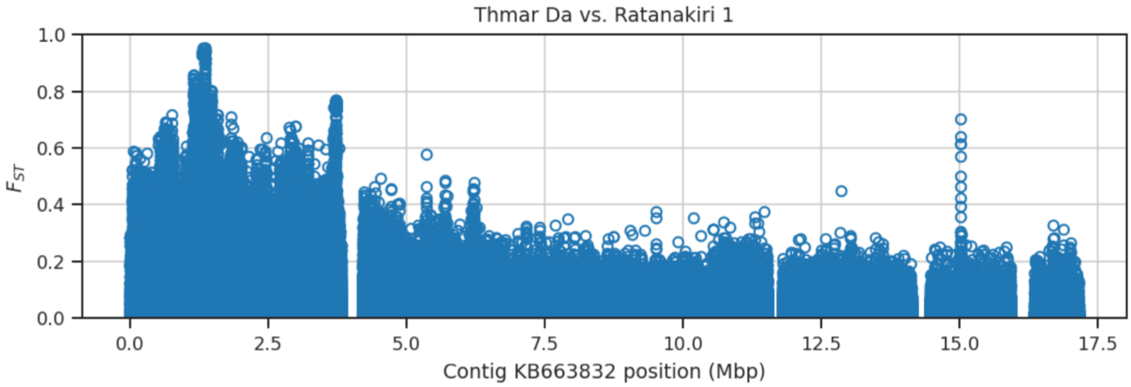

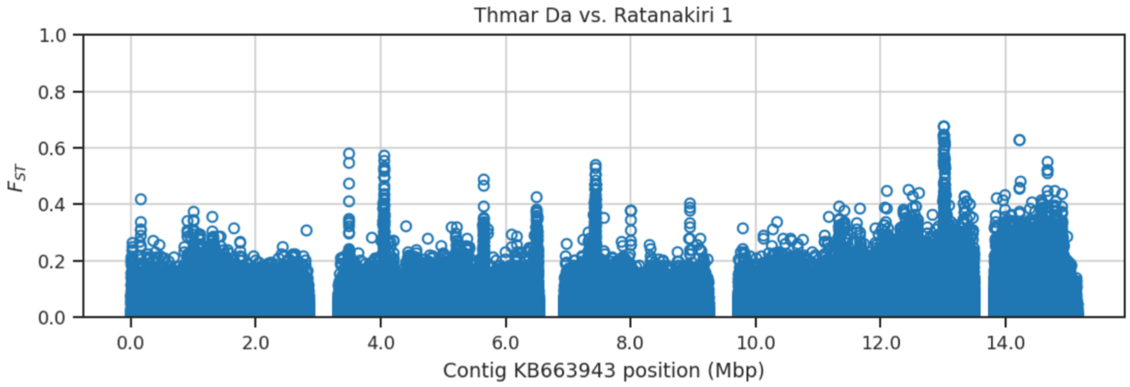

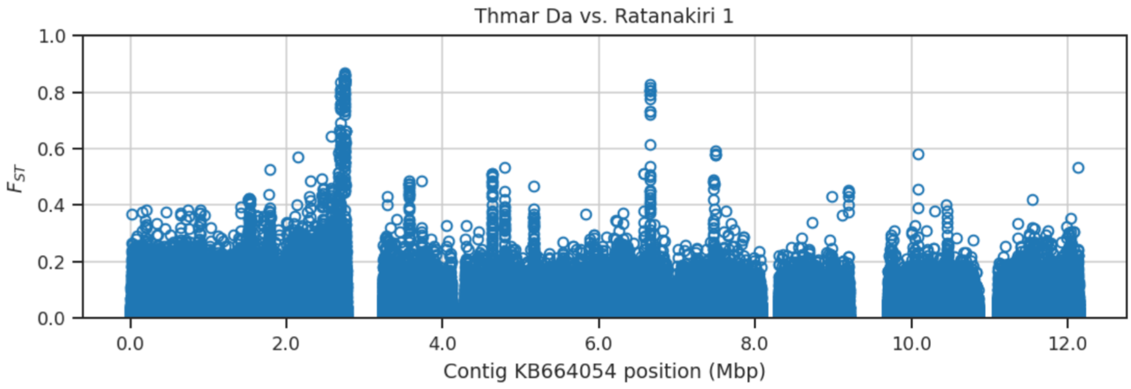

Supplementary Figure 8.5

TD vs. RK1

Raw Fst plots over the 10 largest AminM1 contigs

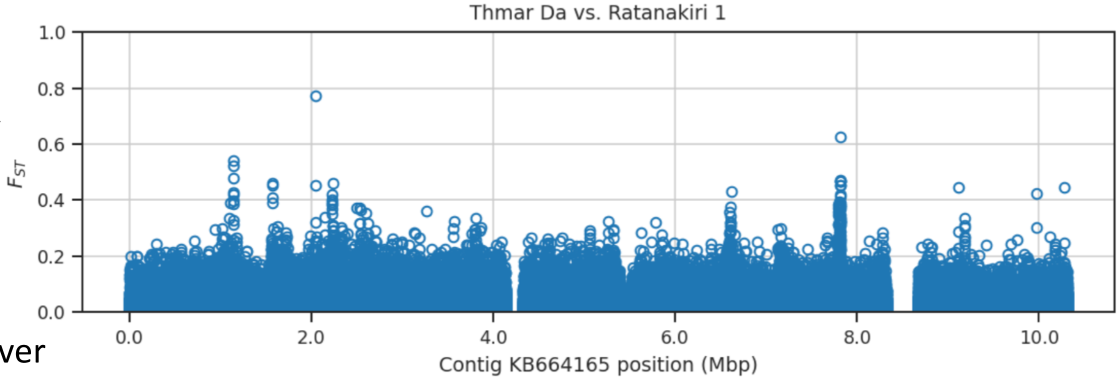

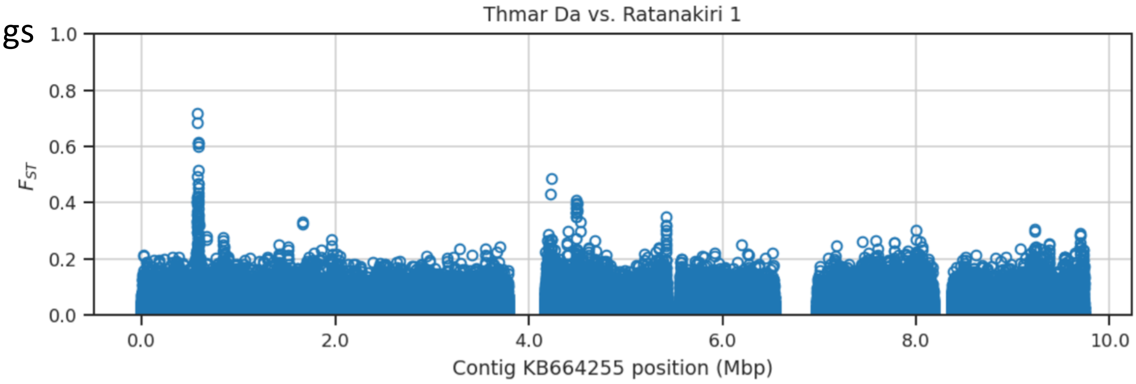

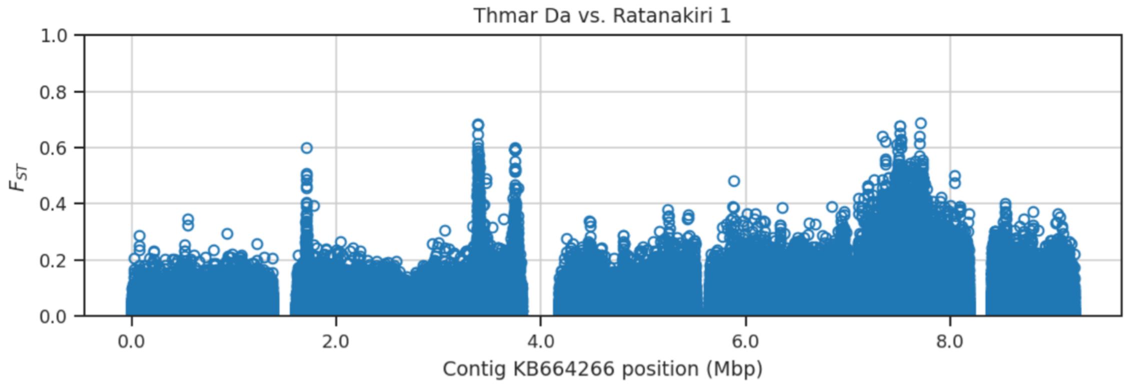

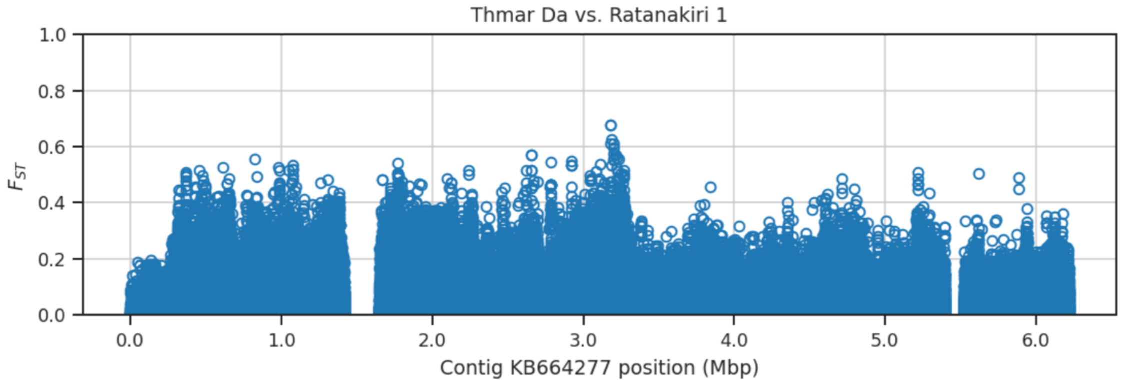

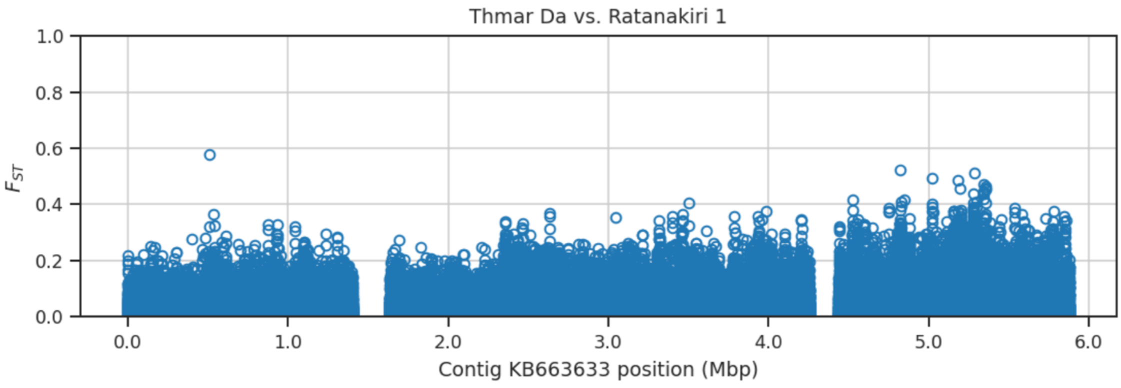

Supplementary Figure 8.6

TD vs. PV

Raw Fst plots over the 10 largest AminM1 contigs

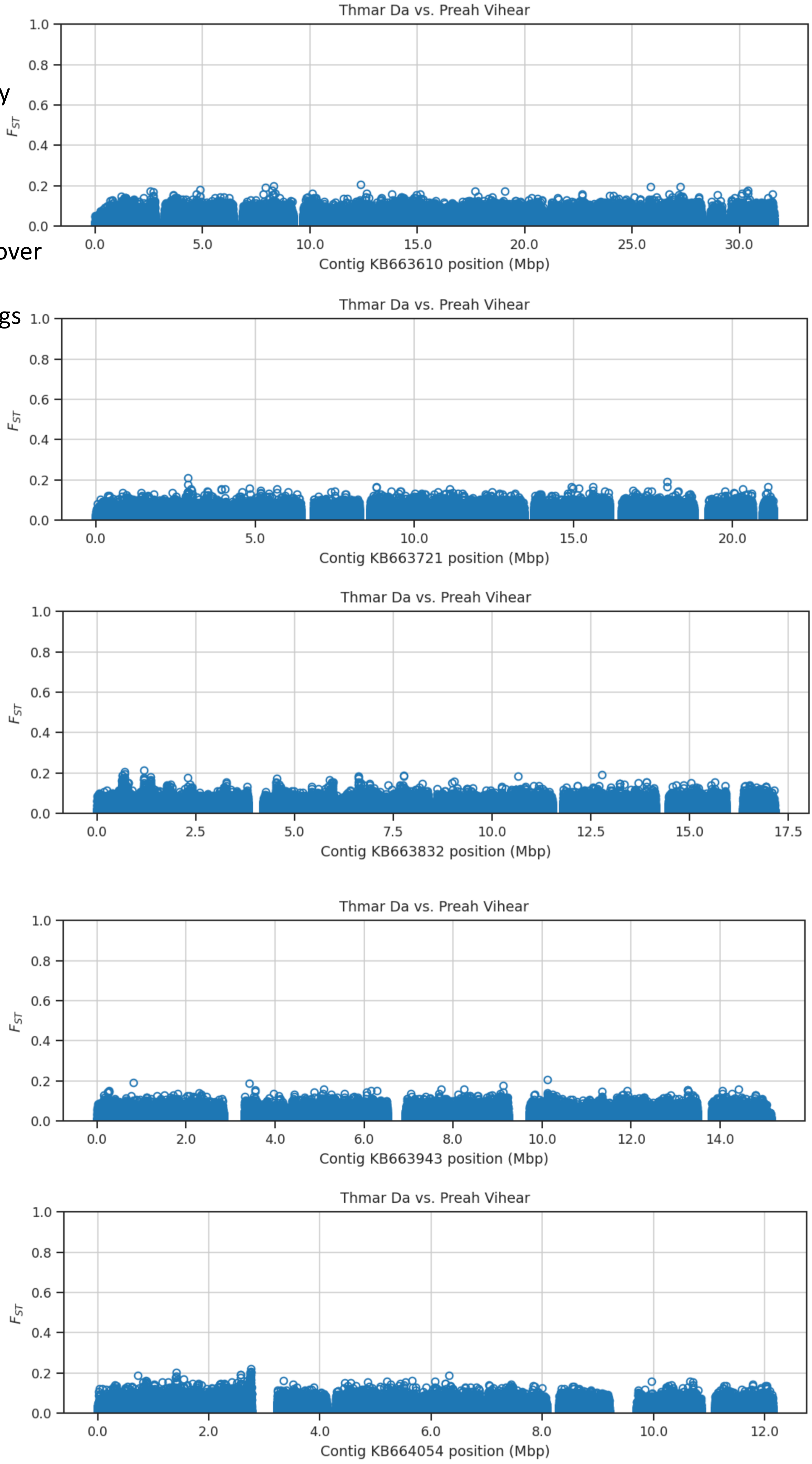

Supplementary Figure 8.6

TD vs. PV

Raw Fst plots over the 10 largest AminM1 contigs

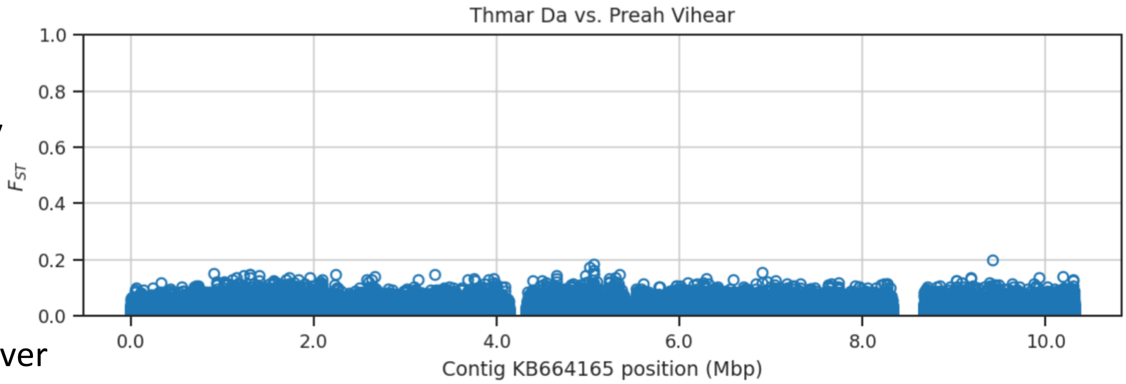

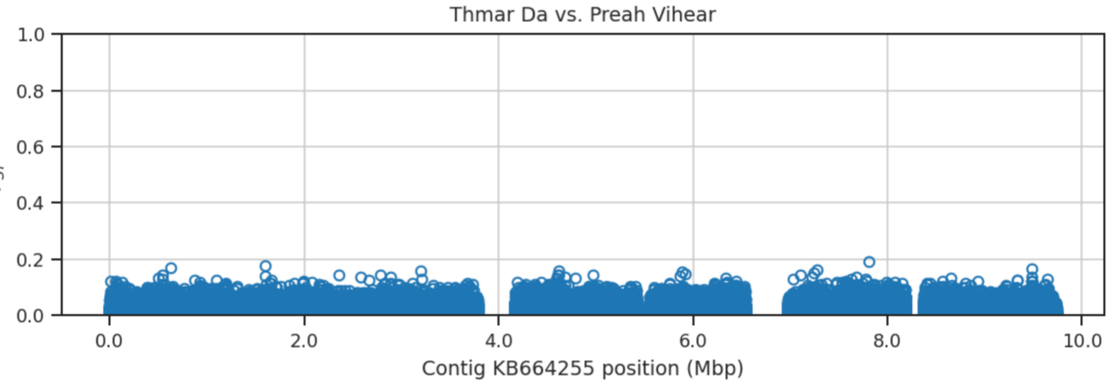

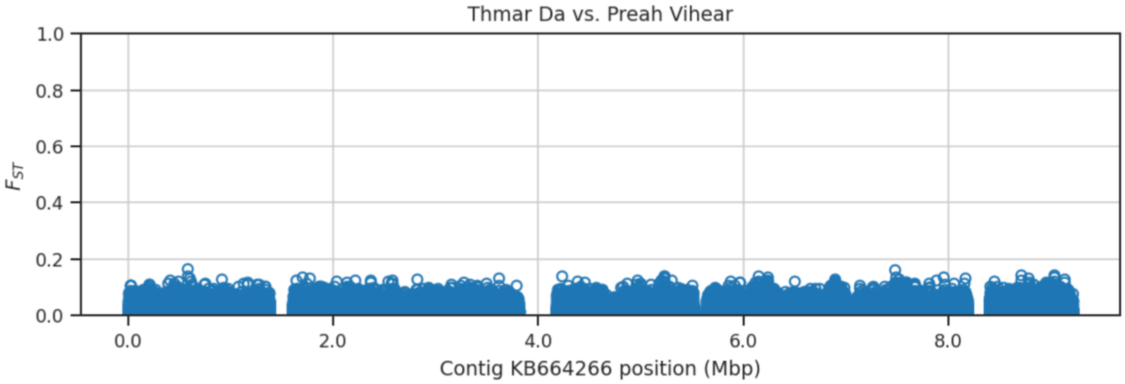

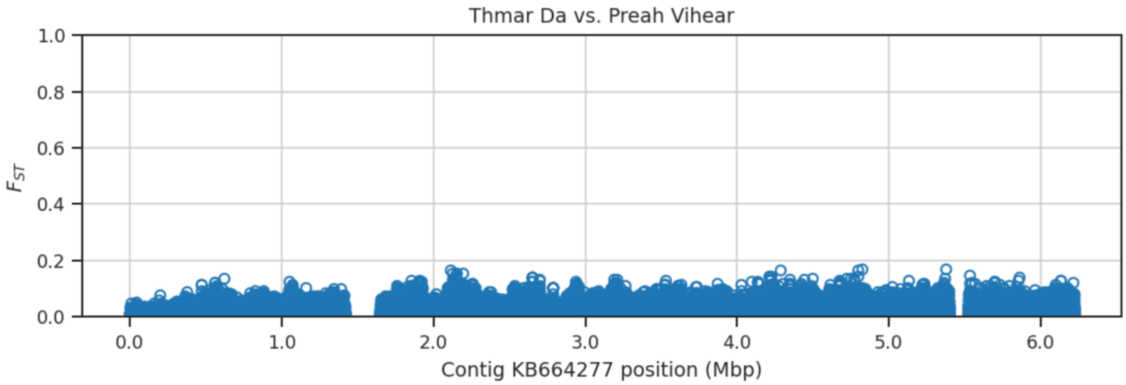

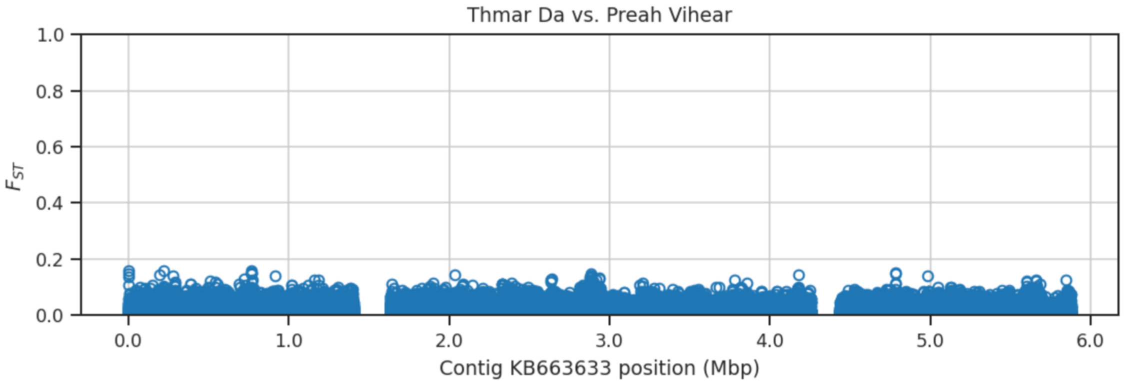

Supplement: Supplementary file 1 — Supplementary materials [file 42003_2022_4259_MOESM1_ESM.pdf]
