# Supplementary material for: Population genomics reveal distinct and diverging populations of An. minimus in Cambodia
Source: Commun Biol. 2022 Nov 28;5:1308. doi: 10.1038/s42003-022-04259-y (PMC9705317; doi:10.1038/s42003-022-04259-y)
Supplement: Supplementary file 2 — Description of Additional Supplementary Files [file 42003_2022_4259_MOESM2_ESM.pdf]

Description of Additional Supplementary Files

File name: Supplementary Data 1-5
Description:

Supplementary Data 1: Sample metadata. This table includes unique sample identifiers and collection metadata for each individual female mosquito included in this study.

Supplementary Data 2: IR SNP variants. SNP variants occurring in over 2% in any within known insecticide-resistance associated genes Ace1, Rdl, KDR, and GSTe2, population are reported here.

Supplementary Data 3: Population Fst. Pariwise average Fst calculations in 20Kb windows for the four populations over the five largest contigs are reported.

Supplementary Data 4: Min contig locations. Summary of largest 40 contigs used for diversity statistics and other calculations in this study, including which Anopheles genomic element and AgamP4 chromosome equivalent arm they lie on.

Supplementary Data 5: Sampling summary. A summary of timepoints and sites where individual An. minimus samples were collected.
