# Supplementary material for: Population genomics reveal distinct and diverging populations of An. minimus in Cambodia
Source: Commun Biol. 2022 Nov 28;5:1308. doi: 10.1038/s42003-022-04259-y (PMC9705317; doi:10.1038/s42003-022-04259-y)
Supplement: Supplementary file 4 — Reporting Summary [file 42003_2022_4259_MOESM4_ESM.pdf]

nature portfolio

Corresponding author(s): Brandyce St. Laurent

Last updated by author(s): Oct 28, 2022

Reporting Summary

Nature Portfolio wishes to improve the reproducibility of the work that we publish. This form provides structure for consistency and transparency in reporting. For further information on Nature Portfolio policies, see our Editorial Policies and the Editorial Policy Checklist.

Statistics

For all statistical analyses, confirm that the following items are present in the figure legend, table legend, main text, or Methods section.

n/a	Confirmed	
☐	☒	The exact sample size (*n*) for each experimental group/condition, given as a discrete number and unit of measurement
☐	☒	A statement on whether measurements were taken from distinct samples or whether the same sample was measured repeatedly
☐	☒	The statistical test(s) used AND whether they are one- or two-sided<br>*Only common tests should be described solely by name; describe more complex techniques in the Methods section.*
☒	☐	A description of all covariates tested
☐	☒	A description of any assumptions or corrections, such as tests of normality and adjustment for multiple comparisons
☐	☒	A full description of the statistical parameters including central tendency (e.g. means) or other basic estimates (e.g. regression coefficient) AND variation (e.g. standard deviation) or associated estimates of uncertainty (e.g. confidence intervals)
☒	☐	For null hypothesis testing, the test statistic (e.g. *F*, *t*, *r*) with confidence intervals, effect sizes, degrees of freedom and *P* value noted<br>*Give P values as exact values whenever suitable.*
☒	☐	For Bayesian analysis, information on the choice of priors and Markov chain Monte Carlo settings
☒	☐	For hierarchical and complex designs, identification of the appropriate level for tests and full reporting of outcomes
☒	☐	Estimates of effect sizes (e.g. Cohen's *d*, Pearson's *r*), indicating how they were calculated

Our web collection on statistics for biologists contains articles on many of the points above.

Software and code

Policy information about availability of computer code

Data collection	No software was used in data collection.
Data analysis	Whole genome sequence alignment was done using BWA version 0.7.15 using bwemem. We performed an analysis of population structure using ADMIXTURE version 1.3.0. PCA, and diversity statistics Pairwise Fst, nucleotide diversity, Tajima's D, and Watterson's Theta were run using scikit-allel version 1.3.3. and scikit-allel version 1.2.0.

For manuscripts utilizing custom algorithms or software that are central to the research but not yet described in published literature, software must be made available to editors and reviewers. We strongly encourage code deposition in a community repository (e.g. GitHub). See the Nature Portfolio guidelines for submitting code & software for further information.

Data

Policy information about availability of data

All manuscripts must include a data availability statement. This statement should provide the following information, where applicable:

- Accession codes, unique identifiers, or web links for publicly available datasets
- A description of any restrictions on data availability
- For clinical datasets or third party data, please ensure that the statement adheres to our policy

These genome and SNP data are available at https://www.malariagen.net/resource/35. These data are hosted in Google Cloud. For more information about downloading data, please see the data download guide at https://malariagen.github.io/vector-data/amin1/intro.html. For more information about accessing data in

the cloud, please see the cloud access guide at https://malariagen.github.io/vector-data/amin1/download.html.

These raw sequence data are publicly available on VectorBase:
https://vectorbase.org/vectorbase/app/record/dataset/DS_dc54305595

Human research participants

Policy information about studies involving human research participants and Sex and Gender in Research.

Reporting on sex and gender	N/A
Population characteristics	N/A
Recruitment	N/A
Ethics oversight	N/A

Note that full information on the approval of the study protocol must also be provided in the manuscript.

Field-specific reporting

Please select the one below that is the best fit for your research. If you are not sure, read the appropriate sections before making your selection.

☒ Life sciences ☐ Behavioural & social sciences ☐ Ecological, evolutionary & environmental sciences

For a reference copy of the document with all sections, see nature.com/documents/nr-reporting-summary-flat.pdf

Life sciences study design

All studies must disclose on these points even when the disclosure is negative.

Sample size	All samples that passed our quality control filters after whole genome sequencing were included in our analyses. In some statistical comparisons, we needed to downsample larger populations to the sample size of the smallest population being compared (n=28). This is described in the manuscript text. Previous statistical estimates of the population sample sizes needed for this type of population comparison as part of the Anopheles gambiae 1000 genomes project have indicated that a population sample size of 10 is sufficient.
Data exclusions	Samples that did not pass our sequence quality control filters were excluded. Ten samples that were clear individual outliers in our PCA analysis were excluded in our final population assignment for further comparison. Nine anomalous samples were also excluded in our final population assignments and analyses.
Replication	All data from this study and code for the analyses are available open-access. These analyses have been run on multiple computers/servers and can be re-run to replicate the results.
Randomization	No randomization was needed
Blinding	No blinding was neccessary

Reporting for specific materials, systems and methods

We require information from authors about some types of materials, experimental systems and methods used in many studies. Here, indicate whether each material, system or method listed is relevant to your study. If you are not sure if a list item applies to your research, read the appropriate section before selecting a response.

Materials & experimental systems

n/a	Involved in the study	
☒	☐	Antibodies
☒	☐	Eukaryotic cell lines
☒	☐	Palaeontology and archaeology
☒	☐	Animals and other organisms
☒	☐	Clinical data
☒	☐	Dual use research of concern

Methods

n/a	Involved in the study	
☒	☐	ChIP-seq
☒	☐	Flow cytometry
☒	☐	MRI-based neuroimaging
